# Checklist of terrestrial Parasitengona mites in Fennoscandia with new species- and distribution records (Acariformes: Prostigmata)

**DOI:** 10.3897/BDJ.7.e36094

**Published:** 2019-06-19

**Authors:** Jeanette Stålstedt, Joanna Łaydanowicz, Pekka T Lehtinen, Johannes Bergsten, Joanna Mąkol

**Affiliations:** 1 Department of Zoology, Swedish Museum of Natural History, Stockholm, Sweden Department of Zoology, Swedish Museum of Natural History Stockholm Sweden; 2 Department of Zoology, Stockholm University, Stockholm, Sweden Department of Zoology, Stockholm University Stockholm Sweden; 3 Department of Invertebrate Systematics and Ecology, Wrocław University of Environmental and Life Sciences, Wrocław, Poland Department of Invertebrate Systematics and Ecology, Wrocław University of Environmental and Life Sciences Wrocław Poland; 4 Zoological Museum, University of Turku, Turku, Finland Zoological Museum, University of Turku Turku Finland; 5 Department of Invertebrate Systematics and Ecology, Wroclaw University of Environmental and Life Sciences, Wroclaw, Poland Department of Invertebrate Systematics and Ecology, Wroclaw University of Environmental and Life Sciences Wroclaw Poland

**Keywords:** Distribution, metadata, GBIF, Norway, Sweden, Finland, chiggers, velvet mites, faunistics

## Abstract

The knowledge of terrestrial Parasitengona in Fennoscandia lies far behind that of their aquatic counterparts, the water mites (Hydrachnidia). Based on new inventories, we provide primary data and an annotated checklist of terrestrial Parasitengona in Fennoscandia including 107 species. Out of these, nineteen species are new findings for the region and five are species potentially new for science. Twenty-three species are new for Norway, fourteen for Finland and eleven for Sweden. The known recorded fauna today of terrestrial Parasitengona is 80 species for Norway, 54 for Sweden and 48 for Finland. Primary data include georeferenced locality data as well as collecting techniques and microhabitat to increase the knowledge on species' habitat requirements.

## Introduction

**Figure 1.**
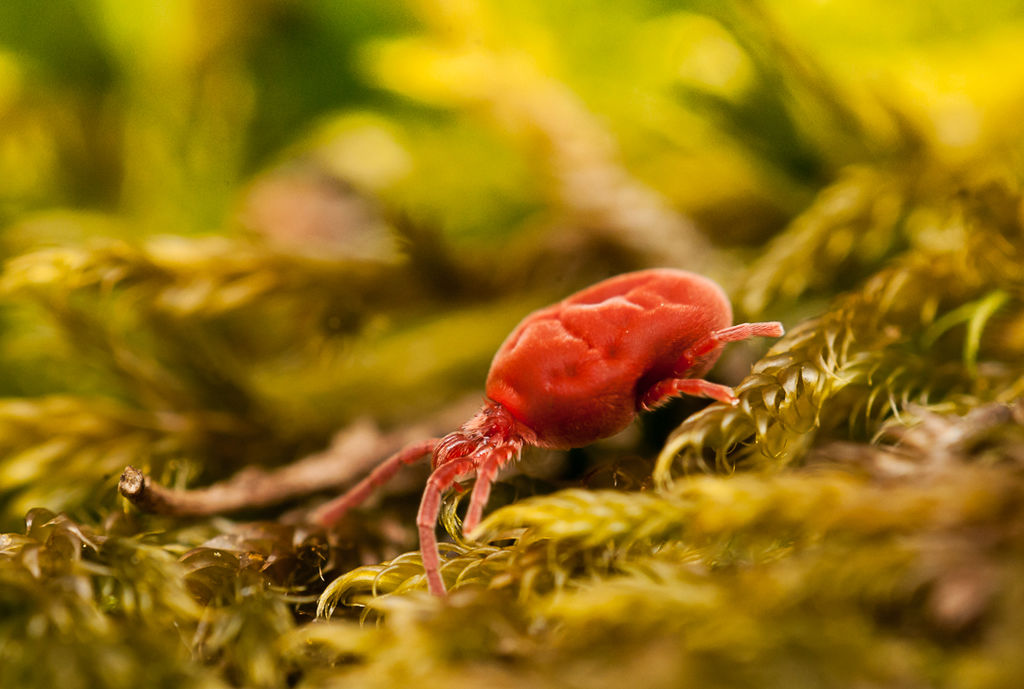
*Allothrombium* sp. Photo Anders Lindström.

**Figure 2.**
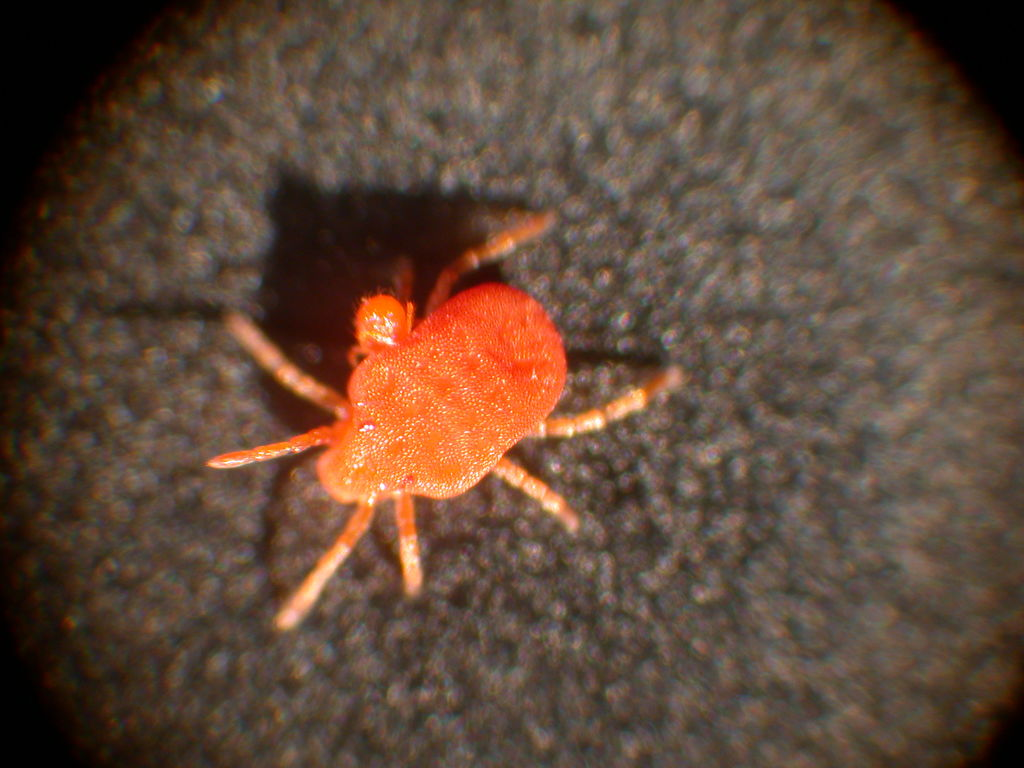
*Calyptostoma* sp. parasitised by larva of *Johnstoniana* sp. Photo Joanna Mąkol.

**Figure 3.**
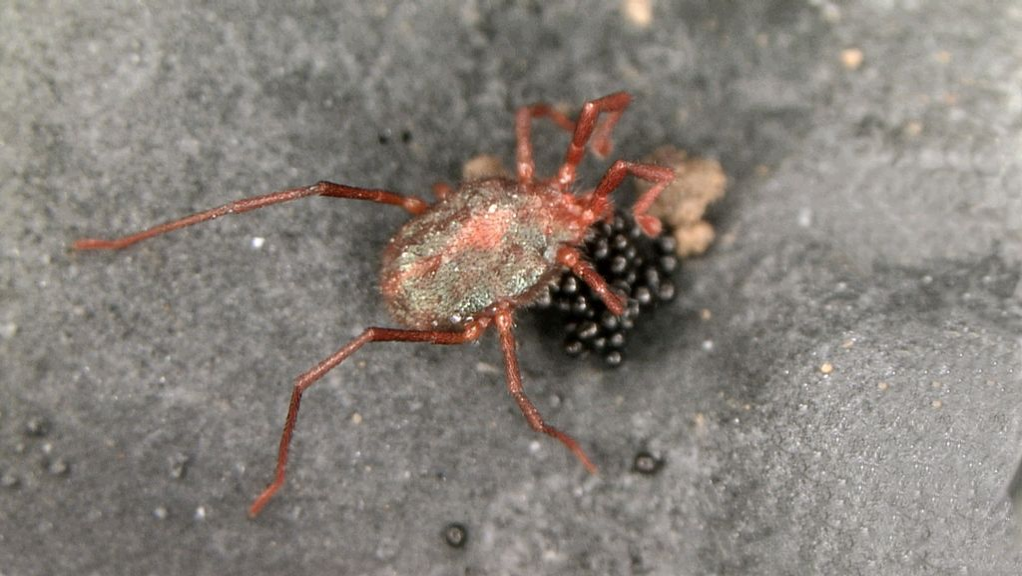
*Erythraeus regalis*. Photo Joanna Mąkol.

**Figure 4.**
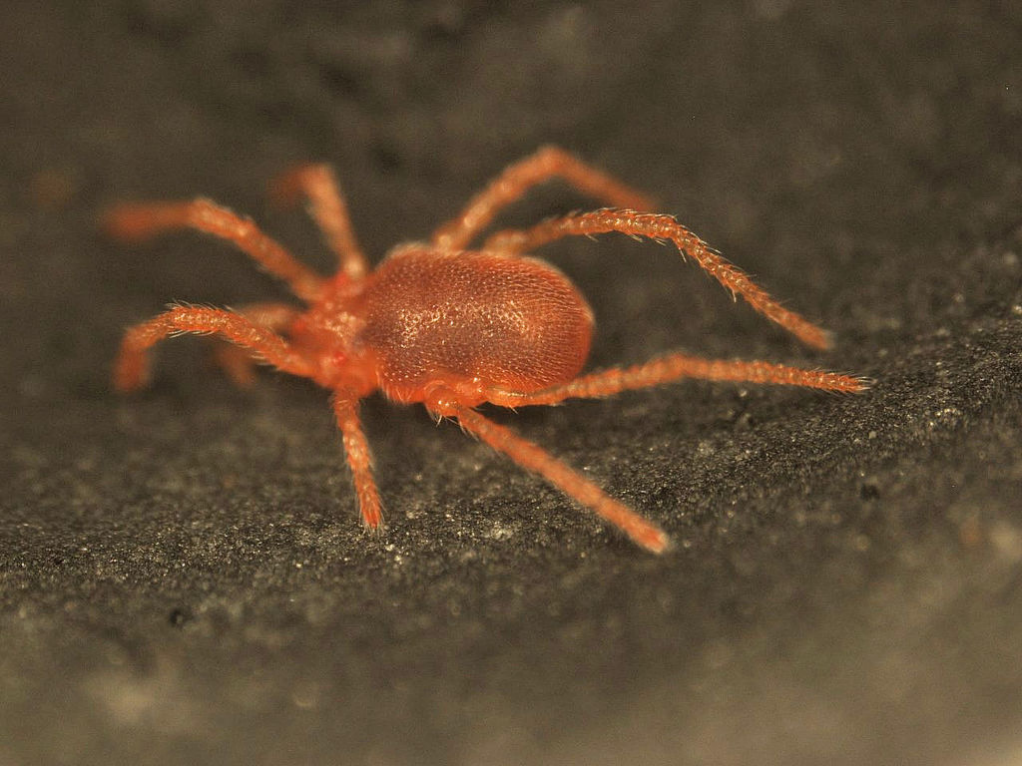
*Johnstoniana* sp. Photo Joanna Mąkol.

**Figure 5.**
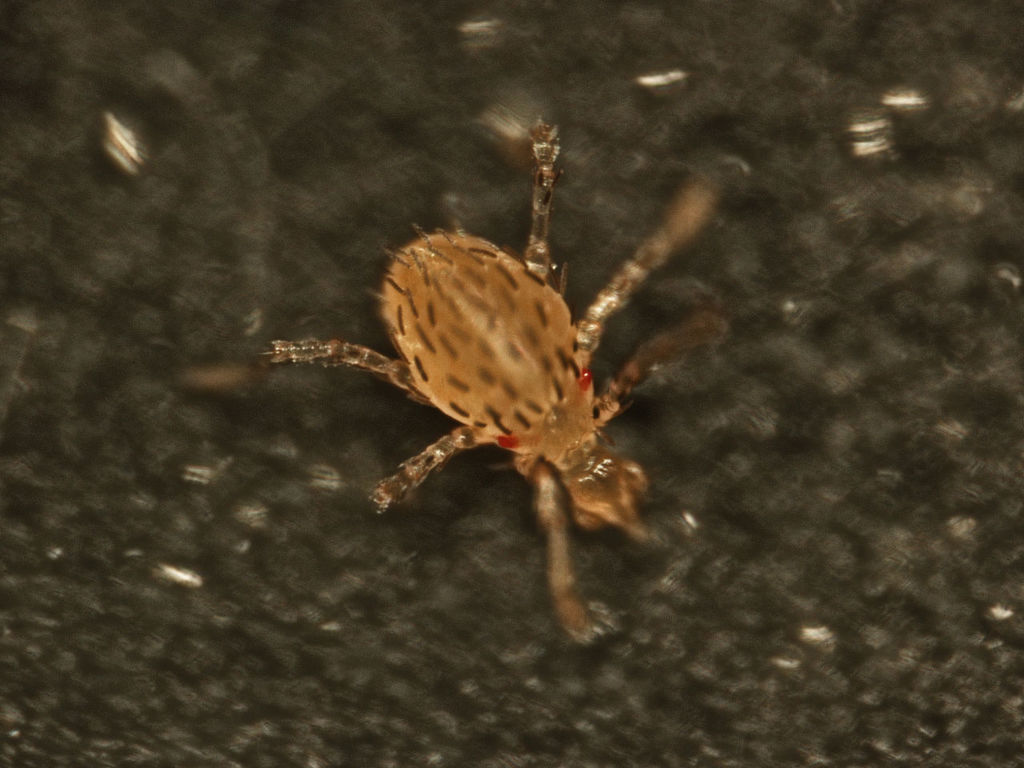
*Leptus* sp. Photo Joanna Mąkol.

**Figure 6.**
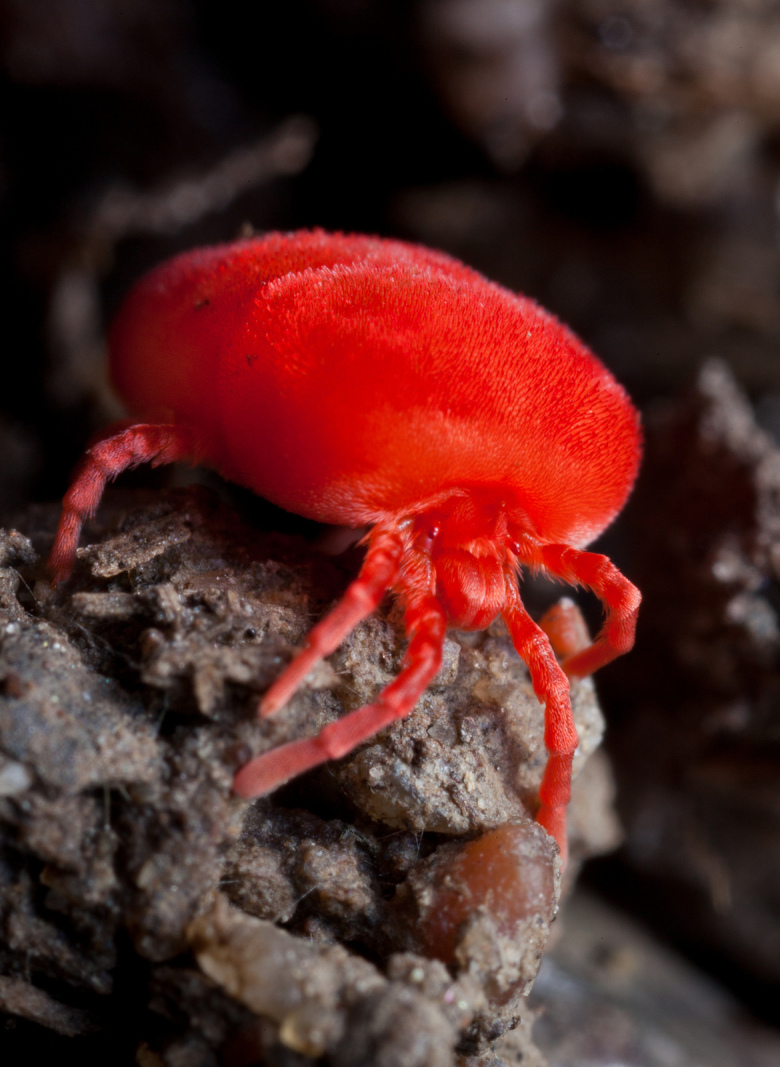
*Trombidium* sp. Photo John Hallmen.

**Figure 7.**
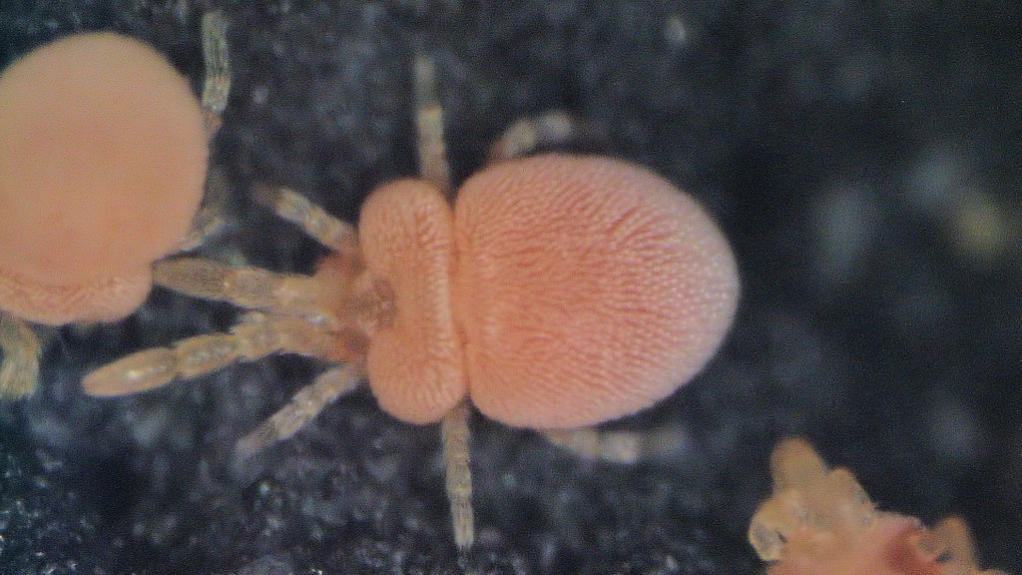
*Leptotrombidium russicum*. Photo Joanna Mąkol.

**Figure 8.**
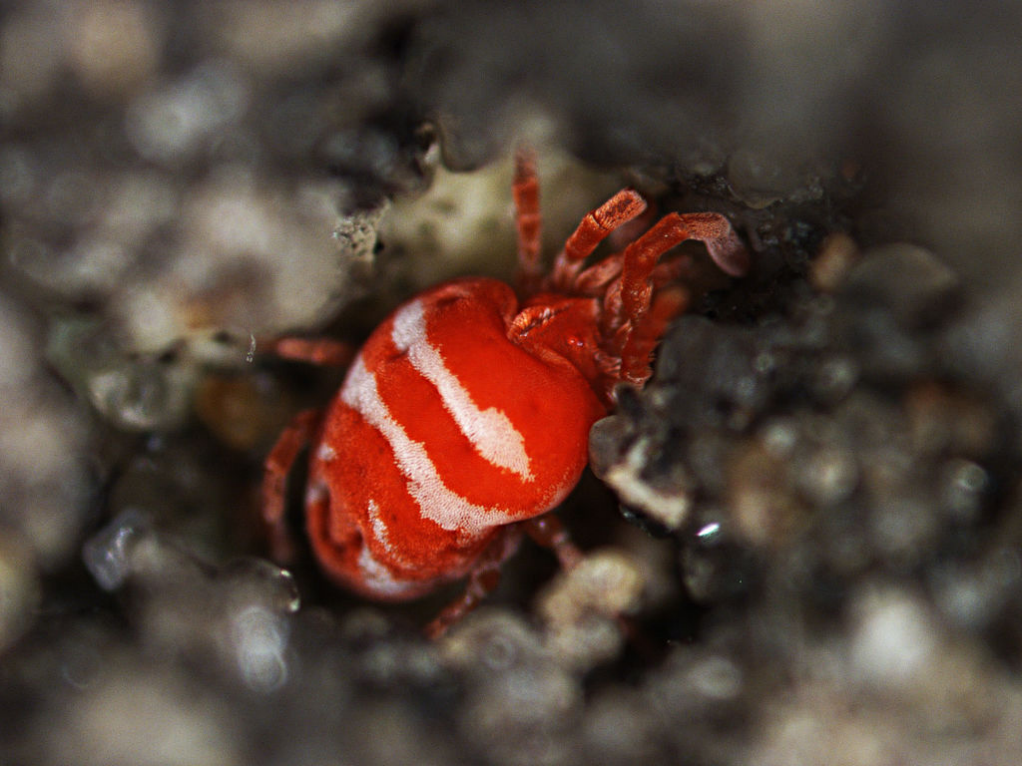
*Platytrombidium fasciatum*. Photo Magdalena Felska.

**Figure 9.**
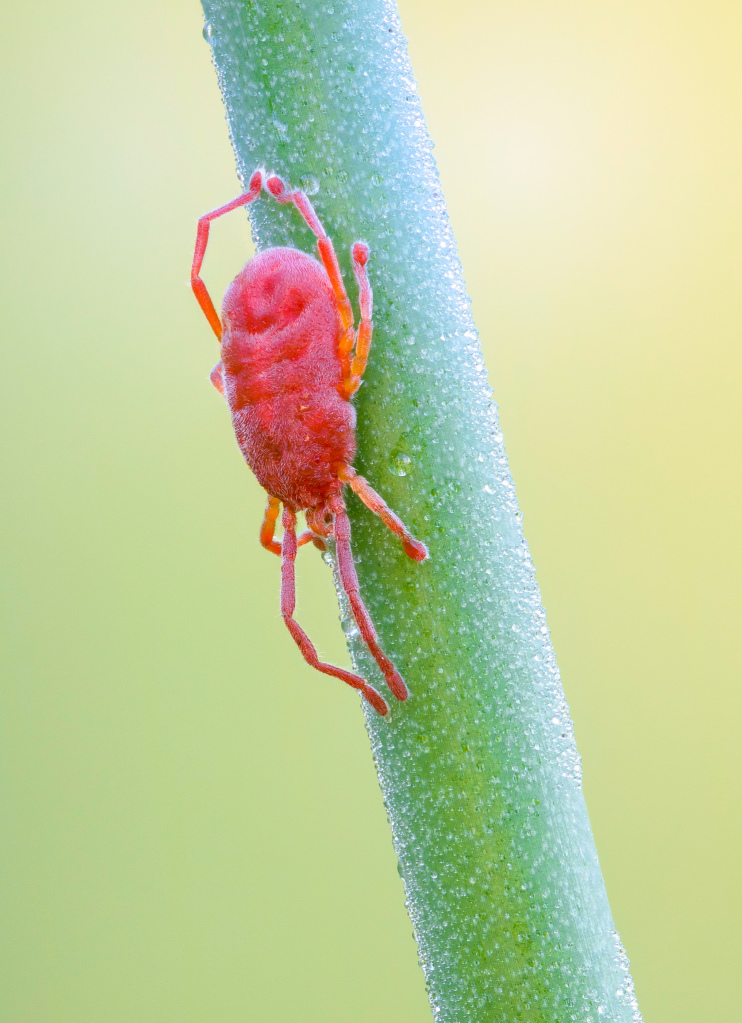
*Abrolophus* sp. Photo John Hallmen.

Parasitengona mites comprise two main ecological assemblages: the aquatic water mites (Hydrachnidia) and the terrestrial parasitengones (Trombidia; Figs 1, 2, 3, 4, 5, 6, 7, 8, 9). Together, the two groups form the monophyletic clade Parasitengona, characterised by their complex life cycle. Larvae of both aquatic and terrestrial lineages parasitise arthropods with few exceptions. The exceptions pertain to predatory or pollinivorous forms and the "chiggers", the vast majority of which have tetrapod vertebrates as hosts. The origin of water mites as either a sister group to, or being derived from within, terrestrial Parasitengona has been debated (Witte 1991, Welbourn 1991), but the latest evidence suggests a nested position of water mites (Dabert et al. 2016, Stålstedt 2017). There have been significant studies of water mites in Fennoscandia by Olov Lundblad, Sig Thor, Pauli Bagge and others (Thor 1897a, Thor 1897b, Thor 1899, Thor 1901, Lundblad 1927, Lundblad 1962, Lundblad 1968, Mehl 1979, Bagge and Bagge 2009, Stålstedt et al. 2013) and, at least in Sweden, the fauna is considered relatively well known. The terrestrial counterpart, Trombidia, however, has been largely ignored and the state of knowledge for the Fennoscandian fauna is very poor. However, in recent years the terrestrial parasitengones of Sogn og Fjordane in Norway (Mąkol and Gulvik 2002), as well as Finnish Erythraeoidea and Trombidiidae (Mąkol et al. 2006, Gabryś et al. 2009) have been studied. Several species known from larvae were recorded from Northern Europe by Haitlinger (2000) and Haitlinger (2008). In Sweden, there have been few studies in recent years. Gärdenfors et al. (2003) summarised the current state of knowledge of multicellular life in Sweden and reported twelve species of Trombidia. This is without doubt a vast underestimate as, for example, 142 species are known from Poland (Mąkol and Wohltmann 2012, Moniuszko and Mąkol 2014, Stålstedt et al. 2016, Mąkol and Korniluk 2017), which is perhaps the best studied country in Europe. Therefore, we here present an updated checklist for Fennoscandia, based on new inventories.

In the checklist, there are 107 species of terrestrial Parasitengona for Fennoscandia. Out of these, nineteen are new findings for the region and five are potential new species for science. In Norway, we present twenty species new for the country, plus three potentially undescribed species. In Sweden, there were nine species new for the country, plus two potentially undescribed species. In Finland, fourteen species were new findings for the country. This raises the known fauna to 80 species recorded for the Norwegian fauna, 54 for Sweden and 48 for Finland. In total, there are 499 records in the checklist, 383 of which are Norwegian.

## Materials and methods

Terrestrial Parasitengona mites were collected between 1966 and 2013 at 131, 23 and 43 different localities in Norway (NOR), Sweden (SWE) and Finland (FIN), respectively. Some localities were visited on multiple occasions (different dates) and some geographical localities (same coordinates) contain multiple collecting events (different microhabitats or methods). Six counties were visited in Norway: Akershus, Buskerud, Hordaland, Oppland, Sogn og Fjordane and Vestfold. In Sweden, sampling took place in eight counties: Jönköping, Kalmar, Skåne, Stockholm, Södermanland, Uppsala, Värmland and Västerbotten. In Finland, seven counties were visited: Central Finland, North Karelia, Northern Ostrobothnia, Northern Savonia, Ostrobothnia, Southwest Finland and Åland Islands. Collectors were Ingvild Austad (IA), Agata Białecka (AB), Jacek Długosz (JD), Magdalena Felska (MF), Maria E. Gulvik (MG), Kalervo Heikka (KH), Rasmus Hovmöller (RH), Ari Karhilahti (AKA), Andrzej Kaźmierski (AK), Małgorzata Kuty (MK), Seppo Koponen (SK), Joanna Łaydanowicz (JŁ), Pekka T. Lehtinen (PTL), Joanna Mąkol (JM), Genowefa Mielnicka (GM), Ritva Niemi (RN), Veikko Rinne (VR), Anna Seniczak (AS), Piotr Skubała (PS), Sabina Słomian (SS), Sławomir Smoliński (SSM), Jeanette Stålstedt (JS), Monika Witkowska (MW) and the Swedish Malaise Trap Project (SMTP). The larva (L) or active postlarva (PL) (i.e. deutonymph (DN) or adult (AD)) were collected with Tullgren funnels (T), sweeping net (C), litter sifting net (S), Malaise trap (M), pitfall trap (P) or directly (U). The information on instars, known for particular species ([L] and/or [PL]), is provided after each species name. When available, each reported specimen is associated with the microhabitat of the collecting circumstances to increase the knowledge of species' ecology and habitat requirements. Records are georeferenced with latitude and longitude coordinates in decimal degree format (WGS84) and altitude in metres, when available. Finnish collectors used a national uniform grid system (yhtenäiskoordinaatisto, YKJ) for mapping localities and this was converted into decimal degrees for the checklist. The locality records are downloadable as a csv file for easy creation of, for example, distribution maps with GIS tools. References to earlier records from Fennoscandia are provided in addition to new locality data. Taxa of uncertain identity (*Trombiium russatum* C.L. Koch, 1837, *Trombidium procerum* C.L. Koch, 1837, *Trombidium hortense* C.L. Koch, 1837, *Trombidium corrugatum* C.L. Koch, 1837, *Trombidium assiratum* C.L. Koch, 1837, *Trombidium erythrellum* C.L. Koch, 1837 and *Rhyncholophus paludicola* C.L. Koch, 1837), recorded by Andersén (1863) from Sweden, are not considered here due to their overall uncertain status followed by likely misidentifications. Each species is linked to the GBIF occurrence database. Under the distribution subheading, only the distribution in Fennoscandia is considered. Species, genus and family level taxonomy follows Mąkol and Wohltmann (2012) and Mąkol and Wohltmann (2013). The latter sources (Mąkol and Wohltmann 2012, Mąkol and Wohltmann 2013) also contain references to the original description of species, with the name in its original combination, author, date and page number of the description. Potential new species (or hitherto unknown instars) will be described elsewhere and are reported as "sp." here, with a note explaining the status. Species with only single occurrences in literature and without recent records in Fennoscandia are considered as questionable identifications.

## Checklists

### Terrestrial Parasitengona

#### Calyptostomatoidea

#### Calyptostomatidae

Oudemans, 1923

#### Calyptostoma velutinum

(Müller, 1776) [PL, L]

http://www.gbif.org/species/4539742

##### Materials

- **Type status:** Other material. **Occurrence:** recordNumber: 1 L; recordedBy: MG; **Location:** county: NOR-Hordaland; locality: In the vicinity of Stanghelle; verbatimElevation: 50; decimalLatitude: 60.5492; decimalLongitude: 5.7344; **Event:** samplingProtocol: T; eventDate: 24/06/2001; habitat: Soil, litter
- **Type status:** Other material. **Occurrence:** recordNumber: 2 L; recordedBy: MG; **Location:** county: NOR-Hordaland; locality: Blinde; verbatimElevation: 100; decimalLatitude: 60.4639; decimalLongitude: 5.3786; **Event:** samplingProtocol: T; eventDate: 04/07/2001; habitat: Moss, litter, soil
- **Type status:** Other material. **Occurrence:** recordNumber: 1 L; recordedBy: MG; **Location:** county: NOR-Hordaland; locality: Haukeland, in the vicinity of Bergen; verbatimElevation: 100; decimalLatitude: 60.3528; decimalLongitude: 5.4478; **Event:** samplingProtocol: T; eventDate: 05/07/2004; habitat: Soil, humus, litter
- **Type status:** Other material. **Occurrence:** recordNumber: 1 AD; recordedBy: MG, JŁ; **Location:** county: NOR-Hordaland; locality: Dale; verbatimElevation: 50; decimalLatitude: 60.5881; decimalLongitude: 5.8178; **Event:** samplingProtocol: T; eventDate: 22/08/2006; habitat: Soil, humus
- **Type status:** Other material. **Occurrence:** recordNumber: 1 L; recordedBy: MG, GM; **Location:** county: NOR-Sogn og Fjordane; locality: Opptun; verbatimElevation: 320; decimalLatitude: 61.5000; decimalLongitude: 7.7203; **Event:** samplingProtocol: T; eventDate: 10/08/2000; habitat: Moss, soil, herbaceous plants, at the base of *Salix caprea*
- **Type status:** Other material. **Occurrence:** recordNumber: 1 AD; recordedBy: MG; **Location:** county: NOR-Sogn og Fjordane; locality: Kaupanger; verbatimElevation: 200; decimalLatitude: 61.1942; decimalLongitude: 7.2192; **Event:** samplingProtocol: T; eventDate: 15/08/2000; habitat: Bark and rot from tree trunk
- **Type status:** Other material. **Occurrence:** recordNumber: 1 DN; recordedBy: MG; **Location:** county: NOR-Sogn og Fjordane; locality: In the vicinity of Sogndal; verbatimElevation: 180; decimalLatitude: 61.2042; decimalLongitude: 7.1983; **Event:** samplingProtocol: T; eventDate: 15/08/2000; habitat: Humic soil
- **Type status:** Other material. **Occurrence:** recordNumber: 1 DN; recordedBy: MG; **Location:** county: NOR-Sogn og Fjordane; locality: Mo, in the vicinity of Moskog; verbatimElevation: 100; decimalLatitude: 61.4364; decimalLongitude: 5.9856; **Event:** samplingProtocol: T; eventDate: 11/10/2000; habitat: Moss, litter, soil
- **Type status:** Other material. **Occurrence:** recordNumber: 1 DN; recordedBy: MG; **Location:** county: NOR-Sogn og Fjordane; locality: In the vicinity of Borgund; verbatimElevation: 400; decimalLatitude: 61.0533; decimalLongitude: 7.8125; **Event:** samplingProtocol: T; eventDate: 07/06/2001; habitat: Soil, litter
- **Type status:** Other material. **Occurrence:** recordNumber: 1 DN; recordedBy: MG; **Location:** county: NOR-Sogn og Fjordane; locality: Galdane; verbatimElevation: 300; decimalLatitude: 61.0611; decimalLongitude: 7.7278; **Event:** samplingProtocol: T; eventDate: 07/06/2001; habitat: Soil, moss, herbaceous plants
- **Type status:** Other material. **Occurrence:** recordNumber: 1 L; recordedBy: MG; **Location:** county: NOR-Sogn og Fjordane; locality: Lærdalsøyri; verbatimElevation: 100; decimalLatitude: 61.0972; decimalLongitude: 7.4911; **Event:** samplingProtocol: T; eventDate: 07/06/2001; habitat: Soil, litter
- **Type status:** Other material. **Occurrence:** recordNumber: 2 AD, 1 L; recordedBy: MG, GM; **Location:** county: NOR-Sogn og Fjordane; locality: Balestrand; verbatimElevation: 100; decimalLatitude: 61.2081; decimalLongitude: 6.5281; **Event:** samplingProtocol: T; eventDate: 31/07/2001; habitat: Soil at the base of decaying tree trunk, herbaceous plants
- **Type status:** Other material. **Occurrence:** recordNumber: 2 DN; recordedBy: MG, GM; **Location:** county: NOR-Sogn og Fjordane; locality: In the vicinity of Urnes; verbatimElevation: 50; decimalLatitude: 61.3072; decimalLongitude: 7.3422; **Event:** samplingProtocol: T; eventDate: 19/08/2001; habitat: Moss, soil
- **Type status:** Other material. **Occurrence:** recordNumber: 1 AD; recordedBy: MG; **Location:** county: NOR-Sogn og Fjordane; locality: In the vicinity of Nystølen; verbatimElevation: 725; decimalLatitude: 61.3442; decimalLongitude: 6.4664; **Event:** samplingProtocol: T; eventDate: 16/09/2001; habitat: Soil, moss, herbaceous plants
- **Type status:** Other material. **Occurrence:** recordNumber: 1 DN; recordedBy: MG; **Location:** county: NOR-Sogn og Fjordane; locality: In the vicinity of Bøyabreen; verbatimElevation: 175; decimalLatitude: 61.4817; decimalLongitude: 6.7406; **Event:** samplingProtocol: T; eventDate: 16/09/2001; habitat: Soil, moss
- **Type status:** Other material. **Occurrence:** recordNumber: 1 AD, 1 L; recordedBy: MG; **Location:** county: NOR-Sogn og Fjordane; locality: Feigum; verbatimElevation: 25; decimalLatitude: 61.3839; decimalLongitude: 7.4239; **Event:** samplingProtocol: T; eventDate: 20/07/2003; habitat: Moss by the creek
- **Type status:** Other material. **Occurrence:** recordNumber: 3 AD; recordedBy: MG, MK; **Location:** county: NOR-Sogn og Fjordane; locality: Sognefjellet, in the vicinity of Turtagrø; verbatimElevation: 1 200; decimalLatitude: 61.5208; decimalLongitude: 7.8258; **Event:** samplingProtocol: T; eventDate: 04/08/2003; habitat: Soil, moss, herbaceous plants
- **Type status:** Other material. **Occurrence:** recordNumber: 2 L; recordedBy: MG, AS; **Location:** county: NOR-Sogn og Fjordane; locality: Vikafjellet; verbatimElevation: 890; decimalLatitude: 60.9700; decimalLongitude: 6.5025; **Event:** samplingProtocol: T; eventDate: 05/07/2004; habitat: Moss by the creek
- **Type status:** Other material. **Occurrence:** recordNumber: 2 AD, 1 DN; recordedBy: MG, GM; **Location:** county: NOR-Sogn og Fjordane; locality: In the vicinity of Opptun; verbatimElevation: 600; decimalLatitude: 61.4978; decimalLongitude: 7.7492; **Event:** samplingProtocol: T; eventDate: 08/05/2005; habitat: Soil, litter, close to creek
- **Type status:** Other material. **Occurrence:** recordNumber: 1 AD; recordedBy: MG; **Location:** county: NOR-Sogn og Fjordane; locality: Between Opptun and Fortun; verbatimElevation: 600; decimalLatitude: 61.4978; decimalLongitude: 7.7492; **Event:** samplingProtocol: T; eventDate: 15/11/2005; habitat: Soil
- **Type status:** Other material. **Occurrence:** recordNumber: 2 AD, 28 L; recordedBy: MG; **Location:** county: NOR-Sogn og Fjordane; locality: Between Skjolden and Fortun; verbatimElevation: 25; decimalLatitude: 61.4878; decimalLongitude: 7.6594; **Event:** samplingProtocol: T; eventDate: 25/11/2005; habitat: Moss, soil
- **Type status:** Other material. **Occurrence:** recordNumber: 1 AD, 1 L; recordedBy: JŁ, MG; **Location:** county: NOR-Sogn og Fjordane; locality: In the vicinity of Opptun; verbatimElevation: 600; decimalLatitude: 61.4978; decimalLongitude: 7.7492; **Event:** samplingProtocol: U; eventDate: 01/07/2006; habitat: Soil, cow parsley, wood stitchwort, side of the road
- **Type status:** Other material. **Occurrence:** recordNumber: 1 L; recordedBy: JŁ, MG; **Location:** county: NOR-Sogn og Fjordane; locality: Opptun; verbatimElevation: 400; decimalLatitude: 61.4997; decimalLongitude: 7.7247; **Event:** samplingProtocol: T; eventDate: 07/08/2006; habitat: Moss and humic soil, by the creek
- **Type status:** Other material. **Occurrence:** recordNumber: 1 DN; recordedBy: MG; **Location:** county: NOR-Vestfold; locality: Between Larvik and Kvelde; verbatimElevation: 25; decimalLatitude: 59.1525; decimalLongitude: 10.0244; **Event:** samplingProtocol: T; eventDate: 03/10/2002; habitat: Soil, litter
- **Type status:** Other material. **Occurrence:** recordNumber: 1 AD; recordedBy: MG; **Location:** county: NOR-Vestfold; locality: Rien, in the vicinity of Odberg; verbatimElevation: 50; decimalLatitude: 59.2708; decimalLongitude: 9.9300; **Event:** samplingProtocol: T; eventDate: 03/10/2002; habitat: Soil, litter
- **Type status:** Other material. **Occurrence:** recordNumber: 2 PL; recordedBy: JS; **Location:** county: SWE-Kalmar; locality: Öland; verbatimElevation: 40; decimalLatitude: 56.6624; decimalLongitude: 16.5896; **Event:** samplingProtocol: S; eventDate: 12/08/2013; habitat: Detritus of leaves, sheep pasture with hazels near wetland
- **Type status:** Other material. **Occurrence:** recordNumber: 2 L; recordedBy: SMTP; **Location:** county: SWE-Uppsala; locality: Biskops-Arnö (=TrapID 8); verbatimElevation: 10; decimalLatitude: 59.6721; decimalLongitude: 17.5009; **Event:** samplingProtocol: M; eventDate: 10/09/2004 - 17/12/2004 (=Coll.ID 1563); habitat: Northern beach, elm grove
- **Type status:** Other material. **Occurrence:** recordNumber: 1 PL; recordedBy: JS, MF, RH; **Location:** county: SWE-Värmland; locality: Ransäter, Prästmyren; verbatimElevation: 115; decimalLatitude: 59.7883; decimalLongitude: 13.4348; **Event:** samplingProtocol: S; eventDate: 04/06/2013; habitat: Arable land, grass, fine sediment, near pine forest and smaller forest stream
- **Type status:** Other material. **Occurrence:** recordNumber: 1 PL; recordedBy: JS, MF, RH; **Location:** county: SWE-Värmland; locality: Ransäter, Ransberg Herrgård; verbatimElevation: 110; decimalLatitude: 59.7903; decimalLongitude: 13.4160; **Event:** samplingProtocol: U, S; eventDate: 05/06/2013; habitat: Moss, fern, mixed deciduous forest in stream ravine
- **Type status:** Other material. **Occurrence:** recordNumber: 4 PL; recordedBy: RH; **Location:** county: SWE-Västerbotten; locality: Near Stora Tjulträsk; verbatimElevation: 600; decimalLatitude: 65.9668; decimalLongitude: 16.0605; **Event:** samplingProtocol: U; eventDate: 19/06/2013; habitat: Birch forest, slope
- **Type status:** Other material. **Occurrence:** recordNumber: 1 PL; recordedBy: RH; **Location:** county: SWE-Västerbotten; locality: Stora Tjulträsk; verbatimElevation: 560; decimalLatitude: 65.9645; decimalLongitude: 16.0479; **Event:** samplingProtocol: P; eventDate: 21/06/2013; habitat: Damp area, birch, juniper, next to brook draining into lake
- **Type status:** Other material. **Occurrence:** recordNumber: 1 PL; recordedBy: PTL; **Location:** county: FIN-North Karelia; locality: Lieksa, Vuonisjärvi, Särkijoki; decimalLatitude: 63.2; decimalLongitude: 30.0; **Event:** eventDate: 14/08/1997; habitat: Lakeshore bush
- **Type status:** Other material. **Occurrence:** recordNumber: 2 PL; recordedBy: JM, PTL; **Location:** county: FIN-North Karelia; locality: Ilomantsi, Kuikkalampi, Särkkä; decimalLatitude: 62.8; decimalLongitude: 30.9; **Event:** eventDate: 15/08/1997; habitat: Lakeshore meadow, litter of aspen
- **Type status:** Other material. **Occurrence:** recordNumber: 6 PL; recordedBy: JM; **Location:** county: FIN-North Karelia; locality: Tohmajärvi, Kemie, Kirkkoniemi,; decimalLatitude: 62.8; decimalLongitude: 30.9; **Event:** eventDate: 15/08/1997; habitat: Deciduous forest with *Rhamnus* & alder
- **Type status:** Other material. **Occurrence:** recordNumber: 1 PL; recordedBy: PTL; **Location:** county: FIN-Northern Ostrobothnia; locality: Pyhäjärvi, Vesikoski; **Event:** eventDate: 05/07/1966; habitat: Rocky slope
- **Type status:** Other material. **Occurrence:** recordNumber: 1 PL; recordedBy: PTL; **Location:** county: FIN-Northern Ostrobothnia; locality: Kuusamo, Toranginjärvi; decimalLatitude: 66.2; decimalLongitude: 29.2; **Event:** eventDate: 11/07/1966; habitat: Bog
- **Type status:** Other material. **Occurrence:** recordNumber: 1 fem; recordedBy: PTL; **Location:** county: FIN-Northern Ostrobothnia; locality: Kuusamo, Liikasenvaara Sirkkapuro,; decimalLatitude: 66.2; decimalLongitude: 29.2; **Event:** eventDate: 18/06/1985; habitat: In moss and litter of brook valley
- **Type status:** Other material. **Occurrence:** recordNumber: 1 AD; recordedBy: PTL; **Location:** county: FIN-Northern Ostrobothnia; locality: Lumijoki, Saarenperä; decimalLatitude: 64.9; decimalLongitude: 24.9; **Event:** eventDate: 13/06/1987; habitat: Mixed forest, litter
- **Type status:** Other material. **Occurrence:** recordNumber: 1 PL; recordedBy: PTL; **Location:** county: FIN-Ostrobothnia; locality: Oravainen, Bodholm; decimalLatitude: 63.2; decimalLongitude: 22.2; **Event:** samplingProtocol: P; eventDate: 22/07/1972 - 07/10/1972; habitat: Shore meadow
- **Type status:** Other material. **Occurrence:** recordNumber: 1 PL; recordedBy: PTL; **Location:** county: FIN-Ostrobothnia; locality: Uusikaarlepyy, Jepua, Lapuanjoki; decimalLatitude: 63.4; decimalLongitude: 22.6; **Event:** samplingProtocol: P; eventDate: 22/07/1972 - 07/10/1972
- **Type status:** Other material. **Occurrence:** recordNumber: 1 AD, 1 DN; recordedBy: PTL; **Location:** county: FIN-Southwest Finland; locality: Parainen, Sydmo, Ippos; decimalLatitude: 60.2; decimalLongitude: 22.1; **Event:** eventDate: 13/06/1966
- **Type status:** Other material. **Occurrence:** recordNumber: 1 PL; recordedBy: PTL; **Location:** county: FIN-Southwest Finland; locality: Parainen, Sydmo, Ippos; decimalLatitude: 60.2; decimalLongitude: 22.1; **Event:** eventDate: 24/05/1967
- **Type status:** Other material. **Occurrence:** recordNumber: 2 PL; recordedBy: PTL; **Location:** county: FIN-Southwest Finland; locality: Korppoo, Björkö; decimalLatitude: 59.9; decimalLongitude: 21.6; **Event:** samplingProtocol: P; eventDate: 15/05/1968 - 16/07/1968; habitat: Maritime deciduous forest
- **Type status:** Other material. **Occurrence:** recordNumber: 1 PL; recordedBy: PTL; **Location:** county: FIN-Southwest Finland; locality: Korppoo, Jurmo; decimalLatitude: 59.8; decimalLongitude: 21.5; **Event:** samplingProtocol: P; eventDate: 16/05/1968 - 17/07/1968; habitat: High juniper stand far from the seashore
- **Type status:** Other material. **Occurrence:** recordNumber: 1 AD; recordedBy: PTL; **Location:** county: FIN-Southwest Finland; locality: Parainen, Kurckas; decimalLatitude: 60.2; decimalLongitude: 22.1; **Event:** samplingProtocol: P; eventDate: 17/05/1968 - 05/07/1968; habitat: Meadow in spruce forest
- **Type status:** Other material. **Occurrence:** recordNumber: 1 PL; recordedBy: PTL; **Location:** county: FIN-Southwest Finland; locality: Uusikaupunki, Lyökki, Ukonkari; decimalLatitude: 60.8; decimalLongitude: 21.1; **Event:** eventDate: 25/06/1968; habitat: Small bog
- **Type status:** Other material. **Occurrence:** recordNumber: 1 PL; recordedBy: PTL; **Location:** county: FIN-Southwest Finland; locality: Korppoo, Björkö; decimalLatitude: 59.9; decimalLongitude: 21.6; **Event:** samplingProtocol: P; eventDate: 16/07/1968 - 20/10/1968; habitat: Base of rock
- **Type status:** Other material. **Occurrence:** recordNumber: 2 PL; recordedBy: PTL; **Location:** county: FIN-Southwest Finland; locality: Parainen, Kopparö; **Event:** samplingProtocol: P; eventDate: 15/08/1968 - 09/10/1968; habitat: Shore meadow
- **Type status:** Other material. **Occurrence:** recordNumber: 1 DN, 1 PL; recordedBy: PTL; **Location:** county: FIN-Southwest Finland; locality: Korppoo, Jurmo; decimalLatitude: 59.8; decimalLongitude: 21.5; **Event:** samplingProtocol: P; eventDate: 29/06/1969 - 14/08/1969; habitat: Bog
- **Type status:** Other material. **Occurrence:** recordNumber: 1 AD; recordedBy: PTL; **Location:** county: FIN-Southwest Finland; locality: Korppoo, Lövskär, Hummelskär; decimalLatitude: 60.1; decimalLongitude: 21.2; **Event:** eventDate: 11/07/1982; habitat: Drifted *Fucus* mixed with decaying *Elymus*
- **Type status:** Other material. **Occurrence:** recordNumber: 1 PL; recordedBy: SK; **Location:** county: FIN-Southwest Finland; locality: Kaarina, Karpanmäki; decimalLatitude: 60.3; decimalLongitude: 22.3; **Event:** samplingProtocol: P; eventDate: 09/05/1989 - 13/06/1989
- **Type status:** Other material. **Occurrence:** recordNumber: 1 PL; recordedBy: RN, VR; **Location:** county: FIN-Southwest Finland; locality: Kaarina, Karpanmäki; decimalLatitude: 60.3; decimalLongitude: 22.3; **Event:** samplingProtocol: P; eventDate: 13/06/1989 - 15/07/1989; habitat: Oak forest
- **Type status:** Other material. **Occurrence:** recordNumber: 1 PL; recordedBy: RN, VR; **Location:** county: FIN-Southwest Finland; locality: Turku, Kylämäki, Vähä‑Rasi; decimalLatitude: 60.4; decimalLongitude: 22.3; **Event:** samplingProtocol: P; eventDate: 20/05/1990 - 28/08/1990; habitat: In cellar
- **Type status:** Other material. **Occurrence:** recordNumber: 3 DN, 5 PL; recordedBy: KH, PTL; **Location:** county: FIN-Southwest Finland; locality: Houtskari, Jungfruskär; decimalLatitude: 60.1; decimalLongitude: 21.1; **Event:** samplingProtocol: P; eventDate: 29/05/1990 - 23/07/1990; habitat: Ash grove
- **Type status:** Other material. **Occurrence:** recordNumber: 1 AD, 6 PL; recordedBy: PTL; **Location:** county: FIN-Southwest Finland; locality: Nauvo, Berghamn, Boskär; decimalLatitude: 59.9; decimalLongitude: 21.6; **Event:** eventDate: 30/05/1990; habitat: *Alnus* grove, fungus
- **Type status:** Other material. **Occurrence:** recordNumber: 5 PL; recordedBy: KH, PTL; **Location:** county: FIN-Southwest Finland; locality: Nauvo, Berghamn, Boskär; decimalLatitude: 59.9; decimalLongitude: 21.6; **Event:** samplingProtocol: P; eventDate: 30/05/1990 - 24/07/1990; habitat: Hazel slope and on the cliff
- **Type status:** Other material. **Occurrence:** recordNumber: 2 AD, 2 PL; recordedBy: KH, PTL, RN; **Location:** county: FIN-Southwest Finland; locality: Houtskari, Jungfruskär; decimalLatitude: 60.1; decimalLongitude: 21.1; **Event:** samplingProtocol: P; eventDate: 23/07/1990 - 12/09/1990; habitat: Sandy dry meadow
- **Type status:** Other material. **Occurrence:** recordNumber: 1 PL; recordedBy: PTL; **Location:** county: FIN-Southwest Finland; locality: Nauvo, Berghamn, Boskär; decimalLatitude: 59.9; decimalLongitude: 21.6; **Event:** samplingProtocol: P; eventDate: 24/07/1990 - 13/09/1990; habitat: Dry meadow
- **Type status:** Other material. **Occurrence:** recordNumber: 1 PL; recordedBy: PTL; **Location:** county: FIN-Southwest Finland; locality: Houtskari, Jungfruskär; decimalLatitude: 60.1; decimalLongitude: 21.1; **Event:** eventDate: 12/09/1990; habitat: Water avens in Alnus grove
- **Type status:** Other material. **Occurrence:** recordNumber: 1 AD, 1 PL; recordedBy: KH, PTL; **Location:** county: FIN-Southwest Finland; locality: Houtskari, Jungfruskär; decimalLatitude: 60.1; decimalLongitude: 21.1; **Event:** samplingProtocol: P; eventDate: 29/05/1991 - 23/07/1991; habitat: Ash grove with *Fraxinus* sp.
- **Type status:** Other material. **Occurrence:** recordNumber: 1 DN, 2 PL; recordedBy: KH, PTL; **Location:** county: FIN-Southwest Finland; locality: Houtskari, Jungfruskär; decimalLatitude: 60.1; decimalLongitude: 21.1; **Event:** samplingProtocol: P; eventDate: 29/05/1992 - 23/07/1992; habitat: Sandy meadow
- **Type status:** Other material. **Occurrence:** recordNumber: 1 PL; recordedBy: PTL; **Location:** county: FIN-Southwest Finland; locality: Nauvo, Sandö; decimalLatitude: 60.17; decimalLongitude: 22.09; **Event:** eventDate: 09/09/1992; habitat: Pondside mire with *Calla*, *Comarum*, *Carex* spp.
- **Type status:** Other material. **Occurrence:** recordNumber: 1 DN, 3 PL; recordedBy: KH, PTL; **Location:** county: FIN-Southwest Finland; locality: Houtskari, Jungfruskär; decimalLatitude: 60.1; decimalLongitude: 21.1; **Event:** samplingProtocol: P; eventDate: 29/05/1993 - 23/07/1993; habitat: Cultivated meadow
- **Type status:** Other material. **Occurrence:** recordNumber: 1 PL; recordedBy: PTL; **Location:** county: FIN-Southwest Finland; locality: Houtskari, Björkö; **Event:** eventDate: 03/06/1994; habitat: Decaying fir
- **Type status:** Other material. **Occurrence:** recordNumber: 2 PL, 1 L; recordedBy: PTL; **Location:** county: FIN-Southwest Finland; locality: Parainen, Lapplahti; decimalLatitude: 60.2; decimalLongitude: 21.9; **Event:** eventDate: 06/08/1996; habitat: Moist deciduous forest
- **Type status:** Other material. **Occurrence:** recordNumber: 1 PL; recordedBy: PTL; **Location:** county: FIN-Southwest Finland; locality: Nauvo, Krok Storra Gunkobb; decimalLatitude: 60.08; decimalLongitude: 21.71; **Event:** eventDate: 21/07/1997; habitat: At the base of *Cochlearia danica*
- **Type status:** Other material. **Occurrence:** recordNumber: 1 DN; recordedBy: JM; **Location:** county: FIN-Southwest Finland; locality: Sonkajärvi, Sukeva; decimalLatitude: 63.8; decimalLongitude: 27.4; **Event:** eventDate: 12/08/1997; habitat: Grove (*Salix* sp., *Alnus* sp., *Betula* sp., grass) on the river shore
- **Type status:** Other material. **Occurrence:** recordNumber: 1 PL; recordedBy: PTL; **Location:** county: FIN-Southwest Finland; locality: Nauvo, Sydänperä; decimalLatitude: 60.17; decimalLongitude: 21.72; **Event:** eventDate: 01/09/1997; habitat: Hazel bush close to seashore
- **Type status:** Other material. **Occurrence:** recordNumber: 1 PL; recordedBy: SK; **Location:** county: FIN-Southwest Finland; locality: Rymättylä, Alakylä, Isoluoto; decimalLatitude: 60.30; decimalLongitude: 21.97; **Event:** eventDate: 04/09/1997; habitat: Litter of alder and decaying reed on seashore
- **Type status:** Other material. **Occurrence:** recordNumber: 1 PL; recordedBy: JM; **Location:** county: FIN-Åland Islands; locality: Åland, Mariehamn, Svinö; decimalLatitude: 60.1; decimalLongitude: 19.8; **Event:** samplingProtocol: U; eventDate: 17/08/1997; habitat: Seashore and margin of deciduous forest

##### Distribution

Norway (Thor 1900a, Mąkol and Gulvik 2002), Sweden (Willmann 1943) and new for Finland.

##### Notes

This taxon may represent more than one species (Wohltmann et al. 1999). *Trombidium expalpe* Hermann, 1804 is a junior synonym (Oudemans 1929).

#### Erythraeoidea

#### Erythraeidae

Robineau-Desvoidy, 1828

#### Abrolophinae

Witte, 1995

#### Abrolophus artemisiae

(Schrank, 1803) [PL]

http://www.gbif.org/species/4539829

##### Materials

- **Type status:** Other material. **Occurrence:** recordNumber: 11 ♀, 11 ♂, 1 AD, 3 DN; recordedBy: MG; **Location:** county: NOR-Sogn og Fjordane; locality: Hafslo; verbatimElevation: 325; decimalLatitude: 61.3264; decimalLongitude: 7.2175; **Event:** samplingProtocol: T; eventDate: 15/09/2002; habitat: Soil, undergrowth
- **Type status:** Other material. **Occurrence:** recordNumber: 1 ♀, 9 DN; recordedBy: MG; **Location:** county: NOR-Sogn og Fjordane; locality: Hafslo; verbatimElevation: 325; decimalLatitude: 61.3264; decimalLongitude: 7.2175; **Event:** samplingProtocol: T; eventDate: 24/07/2003; habitat: Soil, undergrowth
- **Type status:** Other material. **Occurrence:** recordNumber: 2 ♂, 2 DN; recordedBy: MG; **Location:** county: NOR-Sogn og Fjordane; locality: Hafslo; verbatimElevation: 325; decimalLatitude: 61.3264; decimalLongitude: 7.2175; **Event:** samplingProtocol: T; eventDate: 08/08/2003; habitat: Soil, undergrowth
- **Type status:** Other material. **Occurrence:** recordNumber: 4 ♀; recordedBy: MG; **Location:** county: NOR-Sogn og Fjordane; locality: Hafslo; verbatimElevation: 325; decimalLatitude: 61.3264; decimalLongitude: 7.2175; **Event:** samplingProtocol: T; eventDate: 20/09/2003; habitat: Soil, undergrowth
- **Type status:** Other material. **Occurrence:** recordNumber: 2 ♀ 4 ♂; recordedBy: MG; **Location:** county: NOR-Sogn og Fjordane; locality: Hafslo; verbatimElevation: 325; decimalLatitude: 61.3264; decimalLongitude: 7.2175; **Event:** samplingProtocol: T; eventDate: 28/09/2003; habitat: Soil, undergrowth
- **Type status:** Other material. **Occurrence:** recordNumber: 2 ♀ 2 ♂, 2 DN; recordedBy: MG; **Location:** county: NOR-Sogn og Fjordane; locality: Between Skjolden and Luster; verbatimElevation: 25; decimalLatitude: 61.4719; decimalLongitude: 7.5461; **Event:** samplingProtocol: T; eventDate: 05/11/2005; habitat: Sand and clayey soil, moss

##### Distribution

Finland (Gabryś et al. 2009) and new for Norway.

#### Abrolophus bohdani

(Haitlinger, 2003) [L]

##### Materials

- **Type status:** Other material. **Occurrence:** recordNumber: 1 L; recordedBy: MG; **Location:** county: NOR-Hordaland; locality: Dale; verbatimElevation: 50; decimalLatitude: 60.5881; decimalLongitude: 5.8178; **Event:** samplingProtocol: T; eventDate: 25/06/2005; habitat: Litter, soil, humus
- **Type status:** Other material. **Occurrence:** recordNumber: 3 L; recordedBy: MG, SS; **Location:** county: NOR-Oppland; locality: Sognefjellet; verbatimElevation: 1 400; decimalLatitude: 61.5600; decimalLongitude: 7.9628; **Event:** samplingProtocol: T; eventDate: 17/05/2003; habitat: Moss and lichens on rocks
- **Type status:** Other material. **Occurrence:** recordNumber: 1 L; recordedBy: MG, PS, SS; **Location:** county: NOR-Sogn og Fjordane; locality: Rørvik; verbatimElevation: 500; decimalLatitude: 61.4047; decimalLongitude: 6.2178; **Event:** samplingProtocol: T; eventDate: 04/07/2002; habitat: Moss, lichens and humus on rocks

##### Distribution

New for Norway.

#### Abrolophus densipapillus

(Schweizer, 1951) [PL]

https://www.gbif.org/species/4539812

##### Distribution

Sweden (Sellnick 1958).

##### Notes

Only single record in literature (Sellnick 1958) and no recent occurrences since then. Identification questionable.

#### Abrolophus longicollis

(Oudemans, 1910) [L]

https://www.gbif.org/species/4539779

##### Materials

- **Type status:** Other material. **Occurrence:** recordNumber: 21 L; recordedBy: JM; **Location:** county: NOR-Sogn og Fjordane; locality: Atløy; verbatimElevation: 100; decimalLatitude: 61.3472; decimalLongitude: 4.9589; **Event:** samplingProtocol: T; eventDate: 22/05/2001; habitat: Grass tussocks on stony beach, humic soil, herbaceous plants, moss

##### Distribution

Sweden (Sellnick 1958) and new for Norway.

#### Abrolophus miniatus

(Hermann, 1804) [PL]

http://www.gbif.org/species/4539799

##### Materials

- **Type status:** Other material. **Occurrence:** recordNumber: 1 ♀; recordedBy: MG; **Location:** county: NOR-Buskerud; locality: Lerberg, in the vicinity of Hokksund; verbatimElevation: 100; decimalLatitude: 59.7772; decimalLongitude: 9.9244; **Event:** samplingProtocol: T; eventDate: 06/06/2001; habitat: Soil, litter
- **Type status:** Other material. **Occurrence:** recordNumber: 1 ♀; recordedBy: MG, AS; **Location:** county: NOR-Hordaland; locality: Evanger; verbatimElevation: 50; decimalLatitude: 60.6472; decimalLongitude: 6.1356; **Event:** samplingProtocol: T; eventDate: 05/07/2004; habitat: Arable area covered with weeds, grass, humus with litter and moss
- **Type status:** Other material. **Occurrence:** recordNumber: 1 DN; recordedBy: MG; **Location:** county: NOR-Hordaland; locality: In the vicinity of Bulken; verbatimElevation: 75; decimalLatitude: 60.6314; decimalLongitude: 6.2744; **Event:** samplingProtocol: T; eventDate: 25/06/2005; habitat: Moss, decomposed bark, humus, at the base of elm tree
- **Type status:** Other material. **Occurrence:** recordNumber: 1 DN; recordedBy: MG, SS; **Location:** county: NOR-Oppland; locality: Sognefjellet; verbatimElevation: 1 400; decimalLatitude: 61.5600; decimalLongitude: 7.9628; **Event:** samplingProtocol: T; eventDate: 17/05/2003; habitat: Lichens on rocks
- **Type status:** Other material. **Occurrence:** recordNumber: 2 DN; recordedBy: MG, SS; **Location:** county: NOR-Oppland; locality: In the vicinity of Bøvertun; verbatimElevation: 940; decimalLatitude: 61.6453; decimalLongitude: 8.1017; **Event:** samplingProtocol: T; eventDate: 17/05/2003; habitat: Pasture at mountain wall, humus with herbaceous plants
- **Type status:** Other material. **Occurrence:** recordNumber: 1 DN; recordedBy: MG, JM; **Location:** county: NOR-Sogn og Fjordane; locality: Hafslo; verbatimElevation: 300; decimalLatitude: 61.3314; decimalLongitude: 7.2144; **Event:** samplingProtocol: T; eventDate: 25/05/2001; habitat: Soil, humus, undergrowth
- **Type status:** Other material. **Occurrence:** recordNumber: 1 DN; recordedBy: MG; **Location:** county: NOR-Sogn og Fjordane; locality: In the vicinity of Skjolden; verbatimElevation: 50; decimalLatitude: 61.4903; decimalLongitude: 7.5736; **Event:** samplingProtocol: T; eventDate: 08/05/2002; habitat: Rock debris, litter and humus
- **Type status:** Other material. **Occurrence:** recordNumber: 2 DN; recordedBy: MG; **Location:** county: NOR-Sogn og Fjordane; locality: In the vicinity of Skjolden; verbatimElevation: 25; decimalLatitude: 61.4950; decimalLongitude: 7.5867; **Event:** samplingProtocol: T; eventDate: 23/03/2005; habitat: Humus at decomposed tree trunk
- **Type status:** Other material. **Occurrence:** recordNumber: 1 ♀; recordedBy: JŁ; **Location:** county: NOR-Sogn og Fjordane; locality: In the vicinity of Skjolden; verbatimElevation: 50; decimalLatitude: 61.4972; decimalLongitude: 7.6108; **Event:** samplingProtocol: U; eventDate: 06/07/2006; habitat: On rocks, under moss
- **Type status:** Other material. **Occurrence:** recordNumber: 4 PL; recordedBy: JS; **Location:** county: SWE-Stockholm; locality: Stockholm, Lappkärret; verbatimElevation: 20; decimalLatitude: 59.3678; decimalLongitude: 18.0694; **Event:** samplingProtocol: S; eventDate: 21/08/2013, 23/08/2013; habitat: On small hill near lake, oak, maple, aspen, litter

##### Distribution

Sweden (Trägårdh 1910, Sellnick 1958), Finland (Karppinen 1958, Gabryś et al. 2009) and new for Norway.

#### Abrolophus norvegicus

(Thor, 1900) [PL, L]

http://www.gbif.org/species/4539827

##### Distribution

Norway (Thor 1900a, Haitlinger 2000, Mąkol and Gulvik 2002), Sweden (Sellnick 1958) and Finland (Haitlinger 2000, Gabryś et al. 2009).

##### Notes

*Hauptmannia brevicollis* Oudemans, 1910 is a junior synonym (Łaydanowicz and Mąkol 2008).

#### Abrolophus nymindegabicus

Haitlinger, 2008 [L]

##### Distribution

Sweden (Haitlinger 2008).

#### Abrolophus quisquiliarus

(Hermann, 1804) [PL, L]

https://www.gbif.org/species/4539810

##### Materials

- **Type status:** Other material. **Occurrence:** recordNumber: 1 DN; recordedBy: MG; **Location:** county: NOR-Buskerud; locality: Noresund; verbatimElevation: 150; decimalLatitude: 60.1800; decimalLongitude: 9.6211; **Event:** samplingProtocol: T; eventDate: 06/06/2001; habitat: Litter
- **Type status:** Other material. **Occurrence:** recordNumber: 1 ♂; recordedBy: MG, JD, SSM; **Location:** county: NOR-Sogn og Fjordane; locality: Near Kjøsnesfjorden, in the vicinity of Kjøsnes; verbatimElevation: 250; decimalLatitude: 61.5494; decimalLongitude: 6.4789; **Event:** samplingProtocol: T; eventDate: 10/11/2000; habitat: Rot from decaying elm tree trunk
- **Type status:** Other material. **Occurrence:** recordNumber: 1 ♂; recordedBy: MG; **Location:** county: NOR-Sogn og Fjordane; locality: Grinde; verbatimElevation: 120; decimalLatitude: 61.1847; decimalLongitude: 6.7406; **Event:** samplingProtocol: T; eventDate: 13/11/2000; habitat: Rot from decaying elm trunk
- **Type status:** Other material. **Occurrence:** recordNumber: 1 DN; recordedBy: JM; **Location:** county: NOR-Sogn og Fjordane; locality: Atløy; verbatimElevation: 100; decimalLatitude: 61.3472; decimalLongitude: 4.9589; **Event:** samplingProtocol: T; eventDate: 22/05/2001; habitat: Grass tussocks on stony beach, humic soil with herbaceous plants and moss
- **Type status:** Other material. **Occurrence:** recordNumber: 1 DN; recordedBy: MG, GM; **Location:** county: NOR-Sogn og Fjordane; locality: Urnes; verbatimElevation: 100; decimalLatitude: 61.2978; decimalLongitude: 7.3242; **Event:** samplingProtocol: T; eventDate: 19/08/2001; habitat: Soil
- **Type status:** Other material. **Occurrence:** recordNumber: 1 DN; recordedBy: MG; **Location:** county: NOR-Sogn og Fjordane; locality: Kusslia, in the vicinity of Moskog; verbatimElevation: 50; decimalLatitude: 61.4347; decimalLongitude: 5.9583; **Event:** samplingProtocol: T; eventDate: 16/09/2001; habitat: On tree trunk of elm
- **Type status:** Other material. **Occurrence:** recordNumber: 1 DN; recordedBy: MG; **Location:** county: NOR-Sogn og Fjordane; locality: Grinde; verbatimElevation: 120; decimalLatitude: 61.1847; decimalLongitude: 6.7406; **Event:** samplingProtocol: T; eventDate: 16/10/2001; habitat: Soil and litter, at the base of tree trunk
- **Type status:** Other material. **Occurrence:** recordNumber: 1 ♀; recordedBy: MG; **Location:** county: NOR-Sogn og Fjordane; locality: Kusslia, in the vicinity of Moskog; verbatimElevation: 50; decimalLatitude: 61.4347; decimalLongitude: 5.9583; **Event:** samplingProtocol: T; eventDate: 18/10/2001; habitat: Soil, undergrowth
- **Type status:** Other material. **Occurrence:** recordNumber: 3 ♀; recordedBy: MG; **Location:** county: NOR-Sogn og Fjordane; locality: Hafslo; verbatimElevation: 325; decimalLatitude: 61.3264; decimalLongitude: 7.2175; **Event:** samplingProtocol: T; eventDate: 15/09/2002; habitat: Soil, undergrowth
- **Type status:** Other material. **Occurrence:** recordNumber: 1 DN; recordedBy: MG; **Location:** county: NOR-Sogn og Fjordane; locality: Hafslo; verbatimElevation: 325; decimalLatitude: 61.3264; decimalLongitude: 7.2175; **Event:** samplingProtocol: T; eventDate: 24/07/2003; habitat: Soil, undergrowth
- **Type status:** Other material. **Occurrence:** recordNumber: 2 ♀; recordedBy: MG; **Location:** county: NOR-Sogn og Fjordane; locality: Hafslo; verbatimElevation: 325; decimalLatitude: 61.3264; decimalLongitude: 7.2175; **Event:** samplingProtocol: T; eventDate: 08/08/2003; habitat: Soil, undergrowth
- **Type status:** Other material. **Occurrence:** recordNumber: 1 ♀; recordedBy: MG; **Location:** county: NOR-Sogn og Fjordane; locality: Hafslo; verbatimElevation: 325; decimalLatitude: 61.3264; decimalLongitude: 7.2175; **Event:** samplingProtocol: T; eventDate: 20/09/2003; habitat: Soil, undergrowth

##### Distribution

Sweden (Andersén 1863, Sellnick 1958), Finland (Gabryś et al. 2009) and new for Norway.

#### Abrolophus rhopalicus

(C.L. Koch, 1837) [PL]

https://www.gbif.org/species/4539839

##### Materials

- **Type status:** Other material. **Occurrence:** recordNumber: 1 ♂; recordedBy: MG; **Location:** county: NOR-Buskerud; locality: Hvila; verbatimElevation: 180; decimalLatitude: 59.7553; decimalLongitude: 9.5861; **Event:** samplingProtocol: T; eventDate: 03/10/2002; habitat: Moss, soil, humus
- **Type status:** Other material. **Occurrence:** recordNumber: 2 ♀, 1 DN; recordedBy: MG, AK; **Location:** county: NOR-Oppland; locality: Sognefjellet; verbatimElevation: 1 400; decimalLatitude: 61.5647; decimalLongitude: 7.9947; **Event:** samplingProtocol: T; eventDate: 22/06/2000; habitat: Thick layer of very moist humus with sand, moss on rocks, clayey–humic soil
- **Type status:** Other material. **Occurrence:** recordNumber: 4 DN; recordedBy: MG, PS, SS; **Location:** county: NOR-Sogn og Fjordane; locality: Hov, in the vicinity of Vik; verbatimElevation: 400; decimalLatitude: 61.3231; decimalLongitude: 6.2603; **Event:** samplingProtocol: T; eventDate: 04/07/2002; habitat: Litter, moss, humus
- **Type status:** Other material. **Occurrence:** recordNumber: 1 ♀; recordedBy: MG, PS, SS; **Location:** county: NOR-Sogn og Fjordane; locality: In the vicinity of Nystølen; verbatimElevation: 500; decimalLatitude: 61.3392; decimalLongitude: 6.3536; **Event:** samplingProtocol: T; eventDate: 04/07/2002; habitat: Forest edge, bark and moss from the upper part of moraine
- **Type status:** Other material. **Occurrence:** recordNumber: 1 ♀, 3 DN; recordedBy: MG, PS, SS; **Location:** county: NOR-Sogn og Fjordane; locality: Rørvik; verbatimElevation: 500; decimalLatitude: 61.4047; decimalLongitude: 6.2178; **Event:** samplingProtocol: T; eventDate: 04/07/2002; habitat: Litter, moss, lichens, humus
- **Type status:** Other material. **Occurrence:** recordNumber: 1 ♀, 5 DN; recordedBy: MG, MK; **Location:** county: NOR-Sogn og Fjordane; locality: Sognefjellet, in the vicinity of Turtagrø; verbatimElevation: 1 200; decimalLatitude: 61.5208; decimalLongitude: 7.8258; **Event:** samplingProtocol: T; eventDate: 04/08/2003; habitat: Moss, humus, vegetation, peat substratum

##### Distribution

Sweden (Andersén 1863) and new for Norway.

#### Abrolophus rubipes

(Berlese & Trouessart, 1889) [PL]

http://www.gbif.org/species/4539814

##### Distribution

Sweden (Sellnick 1949).

##### Notes

No recent occurrences since the record of Sellnick (1949). Identification questionable.

#### Abrolophus wratislaviensis

(Haitlinger, 1986) [L]

https://www.gbif.org/species/4539775

##### Distribution

Norway (Haitlinger 2000) and Sweden (Haitlinger 2008).

#### Abrolophus sp.

##### Materials

- **Type status:** Other material. **Occurrence:** recordNumber: 27 L; recordedBy: MG; **Location:** county: NOR-Sogn og Fjordane; locality: Between Skjolden and Luster; verbatimElevation: 100; decimalLatitude: 61.4642; decimalLongitude: 7.5303; **Event:** samplingProtocol: T; eventDate: 14/03/2001; habitat: Moss, soil
- **Type status:** Other material. **Occurrence:** recordNumber: 7 L; recordedBy: MG; **Location:** county: NOR-Sogn og Fjordane; locality: Råum, in the vicinity of Gaupne; verbatimElevation: 100; decimalLatitude: 61.3894; decimalLongitude: 7.3406; **Event:** samplingProtocol: T; eventDate: 11/04/2001; habitat: Moss and rot from decaying birch trunk
- **Type status:** Other material. **Occurrence:** recordNumber: 18 L; recordedBy: MG, MW; **Location:** county: NOR-Sogn og Fjordane; locality: In the vicinity of Skjolden; verbatimElevation: 75; decimalLatitude: 61.4994; decimalLongitude: 7.6097; **Event:** samplingProtocol: T; eventDate: 09/12/2006; habitat: Bark, moss, lichens on decaying birch stumps, decaying raspberry shrubs

##### Notes

Potential new species for science from Norway (or unknown instar of species already known from active postlarval forms).

#### Balaustiinae

Grandjean, 1947

#### Balaustium murorum

(Hermann, 1804) [PL, L]

http://www.gbif.org/species/4336780

##### Distribution

Sweden (Andersén 1863, Sellnick 1949) and Finland (Karppinen 1958, Gabryś et al. 2009).

##### Notes

*Rhyncholophus crocatus* C.L. Koch, 1837 is a junior synonym (Gabryś 2016).

#### Balaustium nikae

Haitlinger, 1996 [L]

http://www.gbif.org/species/4539958

##### Distribution

Sweden (Haitlinger 2008).

#### Balaustium sp.

##### Materials

- **Type status:** Other material. **Occurrence:** recordNumber: 2 AD; recordedBy: SMTP; **Location:** county: SWE-Södermanland; locality: Tullgarns näs, Rävsalaviken (=TrapID 30); verbatimElevation: 15; decimalLatitude: 58.9552; decimalLongitude: 17.6075; **Event:** samplingProtocol: M; eventDate: 03/07/2004 - 19/08/2004 (=Coll.ID 1055); habitat: Mixed forest next to pasture

##### Notes

Potential new species for science from Sweden. Morphologically near *Balaustium araneoides* (Berlese, 1910).

#### Callidosomatinae

Southcott, 1957

#### Charletonia cardinalis

(C.L. Koch, 1837) [PL, L]

http://www.gbif.org/species/4539871

##### Materials

- **Type status:** Other material. **Occurrence:** recordNumber: 1 ♀, 2 ♂; recordedBy: JŁ; **Location:** county: NOR-Sogn og Fjordane; locality: Skjolden; verbatimElevation: 25; decimalLatitude: 61.4911; decimalLongitude: 7.6042; **Event:** samplingProtocol: U; eventDate: 06/07/2006; habitat: Grassy slope by the river, boulder covered with moss
- **Type status:** Other material. **Occurrence:** recordNumber: 1 DN; recordedBy: JS, MF, RH; **Location:** county: SWE-Jönköping; locality: Shore of Lake Landsjön; verbatimElevation: 150; decimalLatitude: 57.8744; decimalLongitude: 14.2991; **Event:** samplingProtocol: U, S; eventDate: 12/06/2013; habitat: Shore with mixed deciduous trees, *Aegopodium*, common reed, moss, bare soil
- **Type status:** Other material. **Occurrence:** recordNumber: 1 L; recordedBy: SMTP; **Location:** county: SWE-Kalmar; locality: Öland (=TrapID 22); verbatimElevation: 40; decimalLatitude: 56.6167; decimalLongitude: 16.5076; **Event:** samplingProtocol: M; eventDate: 20/05/2005 - 01/06/2005 (=Coll.ID 1312); habitat: Meadow with bushes
- **Type status:** Other material. **Occurrence:** recordNumber: 1 L; recordedBy: JS; **Location:** county: SWE-Stockholm; locality: Norrtälje, St. Dylanda; verbatimElevation: 35; decimalLatitude: 59.7078; decimalLongitude: 18.2332; **Event:** samplingProtocol: U; eventDate: 18/06/2011; habitat: Exposed soil in patches, grass, litter, edge of forest and arable area

##### Distribution

Norway (Mąkol and Gulvik 2002), Sweden (Andersén 1863, Southcott 1991) and Finland (Karppinen 1958 [as *Sphaerolophus globiger* Berlese, 1885, misidentification verified in Gabryś et al. 2009], Haitlinger 2000, Gabryś et al. 2009).

##### Notes

*Rhyncholophus principalis* C. L. Koch, 1837 is a junior synonym (Gabryś 2016).

#### Charletonia globigera

(Berlese, 1885) [PL]

https://www.gbif.org/species/4539866

##### Distribution

Norway (Thor 1900a) and Sweden (Sellnick 1958).

#### Erythraeinae

Robineau-Desvoidy, 1828

#### Curteria episcopalis

(C.L. Koch, 1837) [PL, L]

https://www.gbif.org/species/4539985

##### Materials

- **Type status:** Other material. **Occurrence:** recordNumber: 1 AD; recordedBy: JS; **Location:** county: SWE-Stockholm; locality: Norrtälje, Håtö; verbatimElevation: 25; decimalLatitude: 59.7100; decimalLongitude: 18.8904; **Event:** samplingProtocol: U; eventDate: 24/06/2011; habitat: Open field, weekend cottage, rocks

##### Distribution

Sweden (Andersén 1863, Stålstedt et al. 2016) and Finland (Gabryś et al. 2009).

#### Erythraeus adpendiculatus

(Schrank, 1781) [PL]

https://www.gbif.org/species/9193106

##### Materials

- **Type status:** Other material. **Occurrence:** recordNumber: 4 L; recordedBy: SMTP; **Location:** county: SWE-Västerbotten; locality: Kulbäcksliden experimental forest (=TrapID 57); verbatimElevation: 295; decimalLatitude: 64.1545; decimalLongitude: 19.5932; **Event:** samplingProtocol: M; eventDate: 02/12/2003 - 18/06/2004 (=Coll.ID 751); habitat: 15 yr old spruce plantation, blueberry spruce forest

##### Distribution

Finland (Karppinen 1958, Gabryś et al. 2009) and new for Sweden.

#### Erythraeus cinereus

(Dugès, 1834) [PL, L]

http://www.gbif.org/species/6919397

##### Materials

- **Type status:** Other material. **Occurrence:** recordNumber: 2 DN; recordedBy: MG, GM; **Location:** county: NOR-Oppland; locality: Sognefjellet; verbatimElevation: 1 430; decimalLatitude: 61.5600; decimalLongitude: 7.9681; **Event:** samplingProtocol: T; eventDate: 02/09/2000; habitat: Soil, moss, vegetation
- **Type status:** Other material. **Occurrence:** recordNumber: 3 DN; recordedBy: MG, JŁ; **Location:** county: NOR-Oppland; locality: Sognefjellet; verbatimElevation: 1 300; decimalLatitude: 61.5600; decimalLongitude: 8.0014; **Event:** samplingProtocol: T; eventDate: 20/08/2006; habitat: Moss, litter
- **Type status:** Other material. **Occurrence:** recordNumber: 1 ♂; recordedBy: MG; **Location:** county: NOR-Sogn og Fjordane; locality: Near Kjøsnesfjorden, in the vicinity of jøsnes; verbatimElevation: 250; decimalLatitude: 61.5419; decimalLongitude: 6.5378; **Event:** samplingProtocol: T; eventDate: 11/10/2000; habitat: Rot from decaying trunk
- **Type status:** Other material. **Occurrence:** recordNumber: 1 DN; recordedBy: MG; **Location:** county: NOR-Sogn og Fjordane; locality: Kusslia, in the vicinity of Moskog; verbatimElevation: 50; decimalLatitude: 61.4347; decimalLongitude: 5.9583; **Event:** samplingProtocol: T; eventDate: 16/09/2001; habitat: Moss from decaying trunk
- **Type status:** Other material. **Occurrence:** recordNumber: 48 DN; recordedBy: MG; **Location:** county: NOR-Sogn og Fjordane; locality: Hafslo; verbatimElevation: 325; decimalLatitude: 61.3264; decimalLongitude: 7.2175; **Event:** samplingProtocol: T; eventDate: 15/09/2002; habitat: Soil, undergrowth
- **Type status:** Other material. **Occurrence:** recordNumber: 1 DN; recordedBy: MG; **Location:** county: NOR-Sogn og Fjordane; locality: Hafslo; verbatimElevation: 325; decimalLatitude: 61.3264; decimalLongitude: 7.2175; **Event:** samplingProtocol: T; eventDate: 24/07/2003; habitat: Soil, undergrowth
- **Type status:** Other material. **Occurrence:** recordNumber: 1 DN; recordedBy: MG; **Location:** county: NOR-Sogn og Fjordane; locality: Hafslo; verbatimElevation: 325; decimalLatitude: 61.3264; decimalLongitude: 7.2175; **Event:** samplingProtocol: T; eventDate: 20/09/2003; habitat: Soil, undergrowth
- **Type status:** Other material. **Occurrence:** recordNumber: 2 DN; recordedBy: MG; **Location:** county: NOR-Sogn og Fjordane; locality: Hafslo; verbatimElevation: 325; decimalLatitude: 61.3264; decimalLongitude: 7.2175; **Event:** samplingProtocol: T; eventDate: 28/09/2003; habitat: Soil, undergrowth
- **Type status:** Other material. **Occurrence:** recordNumber: 1 ♂; recordedBy: JŁ, MG; **Location:** county: NOR-Sogn og Fjordane; locality: Between Skjolden and Luster; verbatimElevation: 25; decimalLatitude: 61.4719; decimalLongitude: 7.5461; **Event:** samplingProtocol: U; eventDate: 01/07/2006; habitat: Forest edge with numerous ant-hills, moss covered rock debris
- **Type status:** Other material. **Occurrence:** recordNumber: 1 ♀, 1 DN; recordedBy: JŁ; **Location:** county: NOR-Sogn og Fjordane; locality: Skjolden; verbatimElevation: 25; decimalLatitude: 61.4911; decimalLongitude: 7.6042; **Event:** samplingProtocol: U, C; eventDate: 06/07/2006; habitat: Grassy slope by the river, boulder covered with moss
- **Type status:** Other material. **Occurrence:** recordNumber: 1 DN; recordedBy: JŁ; **Location:** county: NOR-Sogn og Fjordane; locality: In the vicinity of Skjolden; verbatimElevation: 50; decimalLatitude: 61.4972; decimalLongitude: 7.6108; **Event:** samplingProtocol: C (collecetd as larva); eventDate: 06/07/2006; habitat: On rocks, under moss
- **Type status:** Other material. **Occurrence:** recordNumber: 6 ♂; recordedBy: JŁ; **Location:** county: NOR-Sogn og Fjordane; locality: In the vicinity of Skjolden; verbatimElevation: 25; decimalLatitude: 61.5000; decimalLongitude: 7.6017; **Event:** samplingProtocol: U; eventDate: 04/09/2007; habitat: Slope near road and birch forest, relatively moist grass, moss, soil
- **Type status:** Other material. **Occurrence:** recordNumber: 3 DN; recordedBy: JŁ; **Location:** county: NOR-Sogn og Fjordane; locality: In the vicinity of Skjolden; verbatimElevation: 75; decimalLatitude: 61.4992; decimalLongitude: 7.6094; **Event:** samplingProtocol: U; eventDate: 17/09/2007; habitat: Slope near road, very moist grass and moss
- **Type status:** Other material. **Occurrence:** recordNumber: 1 DN; recordedBy: JŁ; **Location:** county: NOR-Sogn og Fjordane; locality: In the vicinity of Skjolden; verbatimElevation: 75; decimalLatitude: 61.4992; decimalLongitude: 7.6094; **Event:** samplingProtocol: U; eventDate: 05/10/2007; habitat: Slope near road, very moist grass and moss
- **Type status:** Other material. **Occurrence:** recordNumber: 5 ♀, 15 ♂, 8 DN; recordedBy: JŁ; **Location:** county: NOR-Sogn og Fjordane; locality: In the vicinity of Skjolden; verbatimElevation: 25; decimalLatitude: 61.5000; decimalLongitude: 7.6017; **Event:** samplingProtocol: U; eventDate: 05/10/2007; habitat: Slope near road and birch forest, relatively moist grass, moss, soil
- **Type status:** Other material. **Occurrence:** recordNumber: 50 AD, 10 DN; recordedBy: JS; **Location:** county: SWE-Stockholm; locality: Stockholm, Lappkärret; verbatimElevation: 20; decimalLatitude: 59.3678; decimalLongitude: 18.0694; **Event:** samplingProtocol: S; eventDate: 21/08/2013, 23/08/2013; habitat: On small hill near lake, oak, maple, aspen, litter

##### Distribution

Sweden (Haitlinger 2008, Stålstedt et al. 2016), Finland (Gabryś et al. 2009) and new for Norway.

##### Notes

*Erythraeus jowitae* Haitlinger, 1987 is a junior synonym (Stålstedt et al. 2016).

#### Erythraeus kresnensis

Beron, 1982 [L]

http://www.gbif.org/species/6919386

##### Materials

- **Type status:** Other material. **Occurrence:** recordNumber: 8 L; recordedBy: MG; **Location:** county: NOR-Sogn og Fjordane; locality: Hafslo; verbatimElevation: 325; decimalLatitude: 61.3264; decimalLongitude: 7.2175; **Event:** samplingProtocol: T; eventDate: 24/07/2003; habitat: Soil, undergrowth
- **Type status:** Other material. **Occurrence:** recordNumber: 1 L; recordedBy: MG; **Location:** county: NOR-Sogn og Fjordane; locality: Hafslo; verbatimElevation: 325; decimalLatitude: 61.3264; decimalLongitude: 7.2175; **Event:** samplingProtocol: T; eventDate: 08/08/2003; habitat: Soil, undergrowth

##### Distribution

New for Norway.

#### Erythraeus monikae

Haitlinger, 1987 [L]

http://www.gbif.org/species/6919400

##### Materials

- **Type status:** Other material. **Occurrence:** recordNumber: 1 L; recordedBy: SMTP; **Location:** county: SWE-Stockholm; locality: Svartlöga, Matkrok (=TrapID 26); verbatimElevation: 10; decimalLatitude: 59.5695; decimalLongitude: 19.05299; **Event:** samplingProtocol: M; eventDate: 15/07/2004 - 28/07/2004 (=Coll.ID 1681); habitat: Maritime deciduous wood, small island

##### Distribution

Finland (Haitlinger 2000) and new for Sweden.

#### Erythraeus nivalis

(Heer, 1845) [PL]

https://www.gbif.org/species/101078909

##### Distribution

Sweden (Trägårdh 1902).

##### Notes

*Rhyncholophus intermedius* Trägårdh, 1902 is a junior synonym (Gabryś 2016).

#### Erythraeus opilionoides

(C.L. Koch, 1837) [PL]

http://www.gbif.org/species/6919409

##### Distribution

Norway (Thor 1900a).

##### Notes

Only single record in literature (Thor 1900a) and no recent occurrences since then. Identification questionable.

#### Erythraeus phalangoides

(De Geer, 1778) [PL, L]

http://www.gbif.org/species/8382687

##### Materials

- **Type status:** Other material. **Occurrence:** recordNumber: 2 ♂; recordedBy: JŁ; **Location:** county: NOR-Sogn og Fjordane; locality: Skjolden; verbatimElevation: 25; decimalLatitude: 61.4911; decimalLongitude: 7.6042; **Event:** samplingProtocol: U; eventDate: 06/07/2006; habitat: Grassy slope by the river, boulder and roots covered with moss

##### Distribution

Norway (Oudemans 1927), Sweden (de Geer 1778, Andersén 1863, Trägårdh 1904, Sellnick 1949) and Finland (Karppinen 1958, Gabryś et al. 2009).

##### Notes

*Rhyncholophus arenicola* Andersén, 1863 and *Bochartia adrastus* Southcott, 1961 are junior synonyms (Gabryś 2016, Stålstedt et al. 2016).

#### Erythraeus regalis

(C.L. Koch, 1837) [PL, L]

http://www.gbif.org/species/6919388

##### Materials

- **Type status:** Other material. **Occurrence:** recordNumber: 1 L; recordedBy: MG, PS, SS; **Location:** county: NOR-Sogn og Fjordane; locality: Nystølen; verbatimElevation: 700; decimalLatitude: 61.3436; decimalLongitude: 6.4564; **Event:** samplingProtocol: T; eventDate: 04/07/2002; habitat: Litter, humus, moss
- **Type status:** Other material. **Occurrence:** recordNumber: 200 L; recordedBy: MG; **Location:** county: NOR-Sogn og Fjordane; locality: Hafslo; verbatimElevation: 325; decimalLatitude: 61.3264; decimalLongitude: 7.2175; **Event:** samplingProtocol: T; eventDate: 24/07/2003; habitat: Soil, undergrowth
- **Type status:** Other material. **Occurrence:** recordNumber: 2 L; recordedBy: MG; **Location:** county: NOR-Sogn og Fjordane; locality: Hafslo; verbatimElevation: 325; decimalLatitude: 61.3264; decimalLongitude: 7.2175; **Event:** samplingProtocol: T; eventDate: 08/08/2003; habitat: Soil, undergrowth
- **Type status:** Other material. **Occurrence:** recordNumber: 1 L; recordedBy: MG, JŁ; **Location:** county: NOR-Sogn og Fjordane; locality: Between Luster and Skjolden; verbatimElevation: 100; decimalLatitude: 61.4811; decimalLongitude: 7.5639; **Event:** samplingProtocol: C; eventDate: 01/07/2006; habitat: Tussock grass and polypody meadow, close to hazel forest, bushes by the stream
- **Type status:** Other material. **Occurrence:** recordNumber: 2 L; recordedBy: JŁ; **Location:** county: NOR-Sogn og Fjordane; locality: Skjolden; verbatimElevation: 25; decimalLatitude: 61.4911; decimalLongitude: 7.6042; **Event:** samplingProtocol: C; eventDate: 06/07/2006; habitat: Grassy slope by the river, boulder covered with moss
- **Type status:** Other material. **Occurrence:** recordNumber: 1 L; recordedBy: SMTP; **Location:** county: SWE-Stockholm; locality: Svartlöga, Matkrok (=TrapID 26); verbatimElevation: 11; decimalLatitude: 59.5695; decimalLongitude: 19.05300; **Event:** samplingProtocol: M; eventDate: 15/07/2004 - 28/07/2004 (=Coll.ID 1681); habitat: Maritime deciduous wood, small island

##### Distribution

Norway (Thor 1900a, Haitlinger 2000, Mąkol and Gulvik 2002), Sweden (Sellnick 1958, Sömermaa 1973, Stålstedt et al. 2016) and Finland (Karppinen 1958, Gabryś et al. 2009).

##### Notes

*Bochartia kuyperi* Oudemans, 1910 and *Erythraeus gertrudae* Haitlinger, 1987 are junior synonyms (Stålstedt et al. 2016).

#### Erythraeus rupestris

(L., 1758) [PL]

https://www.gbif.org/species/8807161

##### Distribution

Sweden (Linnaeus 1758, Andersén 1863) and Finland (Karppinen 1958, Gabryś et al. 2009).

##### Notes

*Rhyncholophus imperialis* C. L. Koch, 1837 is a junior synonym (Oudemans 1937).

#### Kamertonia polonica

Gabryś, 2000 [PL]

http://www.gbif.org/species/4539974

##### Distribution

Finland (Gabryś et al. 2009, Roland and Gabryś 2013).

#### Leptinae

Billberg, 1820

#### Leptus coccineus

(Scopoli, 1763) [L]

http://www.gbif.org/species/6919456

##### Distribution

Norway (Oudemans 1927).

##### Notes

Only single record in literature (Oudemans 1927) and no recent occurrences since then. Identification questionable.

#### Leptus laplandicus

Southcott, 1992 [L]

http://www.gbif.org/species/6919562

##### Distribution

Sweden (Southcott 1992).

##### Notes

Species described by Southcott (1992) from material collected by I. Trägårdh in 1907, in Sarek, Swedish Lappland.

#### Leptus longipilis

(Berlese, 1910) [PL]

http://www.gbif.org/species/6919538

##### Distribution

Finland (Gabryś et al. 2009).

#### Leptus mariae

Haitlinger, 1987 [L]

http://www.gbif.org/species/6919566

##### Materials

- **Type status:** Other material. **Occurrence:** recordNumber: 1 L; recordedBy: SMTP; **Location:** county: SWE-Skåne; locality: Stenshuvud national park, Krivarboden (=TrapID 39); verbatimElevation: 30; decimalLatitude: 55.6603; decimalLongitude: 14.2755; **Event:** samplingProtocol: M; eventDate: 25/07/2003 - 08/08/2003 (=Coll.ID 603); habitat: Broad-leaved deciduous forest
- **Type status:** Other material. **Occurrence:** recordNumber: 5 L; recordedBy: SMTP; **Location:** county: SWE-Södermanland; locality: Tullgarns näs, Rävsalaviken (=TrapID 30); verbatimElevation: 15; decimalLatitude: 58.9552; decimalLongitude: 17.6075; **Event:** samplingProtocol: M; eventDate: 03/07/2004 - 19/08/2004 (=Coll.ID 1055); habitat: Mixed forest next to pasture
- **Type status:** Other material. **Occurrence:** recordNumber: 1 L; recordedBy: SMTP; **Location:** county: SWE-Värmland; locality: Ransäter, Rudstorp (=TrapID 1002); verbatimElevation: 85; decimalLatitude: 59.7730; decimalLongitude: 13.4737; **Event:** samplingProtocol: M; eventDate: 07/07/2005 - 15/07/2005 (=Coll.ID 1375); habitat: Sandy railway embankment through pasture-land

##### Distribution

Norway (Haitlinger 2000) and Sweden (Haitlinger 2008).

#### Leptus molochinus

(C. L. Koch, 1837) [PL, L]

https://www.gbif.org/species/8912762

##### Materials

- **Type status:** Other material. **Occurrence:** recordNumber: 43 L; recordedBy: MG; **Location:** county: NOR-Sogn og Fjordane; locality: Hafslo; verbatimElevation: 325; decimalLatitude: 61.3264; decimalLongitude: 7.2175; **Event:** samplingProtocol: T; eventDate: 24/07/2003; habitat: Soil, undergrowth
- **Type status:** Other material. **Occurrence:** recordNumber: 1 ♀, 4 L; recordedBy: MG; **Location:** county: NOR-Sogn og Fjordane; locality: Hafslo; verbatimElevation: 325; decimalLatitude: 61.3264; decimalLongitude: 7.2175; **Event:** samplingProtocol: T; eventDate: 08/08/2003; habitat: Soil, undergrowth
- **Type status:** Other material. **Occurrence:** recordNumber: 1 DN; recordedBy: MG, MW; **Location:** county: NOR-Sogn og Fjordane; locality: In the vicinity of Skjolden; verbatimElevation: 100; decimalLatitude: 61.4964; decimalLongitude: 7.6106; **Event:** samplingProtocol: T; eventDate: 28/04/2004; habitat: Meadow with concrete walls, herbaceous plants, moss on stony bedrock
- **Type status:** Other material. **Occurrence:** recordNumber: 6 L; recordedBy: JŁ; **Location:** county: NOR-Sogn og Fjordane; locality: Skjolden; verbatimElevation: 25; decimalLatitude: 61.4911; decimalLongitude: 7.6042; **Event:** samplingProtocol: U (4 L), C (2 L); eventDate: 06/07/2006; habitat: Grassy slope by the river, boulder covered with moss
- **Type status:** Other material. **Occurrence:** recordNumber: 4 PL; recordedBy: JS; **Location:** county: SWE-Stockholm; locality: Stockholm, Lappkärret; verbatimElevation: 20; decimalLatitude: 59.3678; decimalLongitude: 18.0694; **Event:** samplingProtocol: S; eventDate: 21/08/2013, 23/08/2013; habitat: On small hill near lake, oak, maple, aspen, litter
- **Type status:** Other material. **Occurrence:** recordNumber: 2 L; recordedBy: SMTP; **Location:** county: SWE-Södermanland; locality: Trosa, Hunga Södergård (=TrapID 12); verbatimElevation: 20; decimalLatitude: 58.9207; decimalLongitude: 17.5212; **Event:** samplingProtocol: M; eventDate: 20/07/2003 - 02/08/2003 (=Coll.ID 175); habitat: Tall grass close to pile of manure behind stable
- **Type status:** Other material. **Occurrence:** recordNumber: 1 L; recordedBy: SMTP; **Location:** county: SWE-Södermanland; locality: Trosa, Hunga Södergård (=TrapID 12); verbatimElevation: 20; decimalLatitude: 58.9207; decimalLongitude: 17.5212; **Event:** samplingProtocol: M; eventDate: 25/08/2003 - 19/09/2003 (=Coll.ID 178); habitat: Tall grass close to pile of manure behind stable
- **Type status:** Other material. **Occurrence:** recordNumber: 1 L; recordedBy: SMTP; **Location:** county: SWE-Södermanland; locality: Tullgarns näs, Rävsalaviken (=TrapID 30); verbatimElevation: 15; decimalLatitude: 58.9552; decimalLongitude: 17.6075; **Event:** samplingProtocol: M; eventDate: 03/07/2004 - 19/08/2004 (=Coll.ID 1055); habitat: Mixed forest next to pasture
- **Type status:** Other material. **Occurrence:** recordNumber: 1 L; recordedBy: SMTP; **Location:** county: SWE-Västerbotten; locality: Kulbäcksängarna (=TrapID 54); verbatimElevation: 155; decimalLatitude: 64.1902; decimalLongitude: 19.6057; **Event:** samplingProtocol: M; eventDate: 05/08/2004 - 20/08/2004 (=Coll.ID 1254); habitat: Birch wood on fine alluvial sediments

##### Distribution

Norway (Mąkol and Gulvik 2002), Sweden (Andersén 1863, Oudemans 1912, Sellnick 1958) and Finland (Karppinen 1958, Gabryś et al. 2009).

##### Notes

*Erythraeus ignotus* Oudemans, 1903 is a junior synonym (Łaydanowicz and Mąkol 2008).

#### Leptus phalangii

(De Geer, 1778) [PL, L]

https://www.gbif.org/species/9042332

##### Materials

- **Type status:** Other material. **Occurrence:** recordNumber: 2 ♀; recordedBy: MG; **Location:** county: NOR-Akershus; locality: Østmarka; verbatimElevation: 250; decimalLatitude: 59.8425; decimalLongitude: 11.0338; **Event:** samplingProtocol: T; eventDate: 07/06/2002; habitat: Litter
- **Type status:** Other material. **Occurrence:** recordNumber: 1 DN; recordedBy: MG; **Location:** county: NOR-Buskerud; locality: Kløftefoss; verbatimElevation: 150; decimalLatitude: 60.0886; decimalLongitude: 9.8336; **Event:** samplingProtocol: T; eventDate: 26/08/2000; habitat: Litter
- **Type status:** Other material. **Occurrence:** recordNumber: 1 DN; recordedBy: MG; **Location:** county: NOR-Buskerud; locality: In the vicinity of Bromma; verbatimElevation: 150; decimalLatitude: 60.4883; decimalLongitude: 9.1839; **Event:** samplingProtocol: T; eventDate: 07/06/2001; habitat: Humus, litter, undergrowth
- **Type status:** Other material. **Occurrence:** recordNumber: 1 DN; recordedBy: MG; **Location:** county: NOR-Hordaland; locality: Between Bulken and Rekve; verbatimElevation: 60; decimalLatitude: 60.6275; decimalLongitude: 6.2961; **Event:** samplingProtocol: T; eventDate: 24/06/2001; habitat: Moss and rot from decaying birch tree trunk
- **Type status:** Other material. **Occurrence:** recordNumber: 1 ♀; recordedBy: MG; **Location:** county: NOR-Hordaland; locality: Sandviki; verbatimElevation: 50; decimalLatitude: 60.4567; decimalLongitude: 5.6786; **Event:** samplingProtocol: T; eventDate: 05/07/2004; habitat: Moss, humus
- **Type status:** Other material. **Occurrence:** recordNumber: 1 DN; recordedBy: MG; **Location:** county: NOR-Hordaland; locality: Dale; verbatimElevation: 50; decimalLatitude: 60.5881; decimalLongitude: 5.8178; **Event:** samplingProtocol: T; eventDate: 05/07/2004; habitat: Litter and humus on bedrock at the foot of mountains
- **Type status:** Other material. **Occurrence:** recordNumber: 1 DN; recordedBy: MG; **Location:** county: NOR-Sogn og Fjordane; locality: Kaupanger; verbatimElevation: 200; decimalLatitude: 61.1942; decimalLongitude: 7.2192; **Event:** samplingProtocol: T; eventDate: 15/08/2000; habitat: Rot from decaying tree trunk
- **Type status:** Other material. **Occurrence:** recordNumber: 1 DN; recordedBy: MG; **Location:** county: NOR-Sogn og Fjordane; locality: In the vicinity of Bøyabreen; verbatimElevation: 150; decimalLatitude: 61.4811; decimalLongitude: 6.7419; **Event:** samplingProtocol: T; eventDate: 11/10/2000; habitat: Litter
- **Type status:** Other material. **Occurrence:** recordNumber: 1 DN; recordedBy: MG; **Location:** county: NOR-Sogn og Fjordane; locality: Near Kjøsnesfjorden, in the vicinity of Kjøsnes; verbatimElevation: 250; decimalLatitude: 61.5419; decimalLongitude: 6.5378; **Event:** samplingProtocol: T; eventDate: 11/10/2000; habitat: Rot from the inside of decaying elm tree
- **Type status:** Other material. **Occurrence:** recordNumber: 4 DN; recordedBy: MG; **Location:** county: NOR-Sogn og Fjordane; locality: Near Kjøsnesfjorden, in the vicinity of Kjøsnes; verbatimElevation: 250; decimalLatitude: 61.5447; decimalLongitude: 6.5106; **Event:** samplingProtocol: T; eventDate: 11/10/2000; habitat: Litter
- **Type status:** Other material. **Occurrence:** recordNumber: 1 DN; recordedBy: MG, GM; **Location:** county: NOR-Sogn og Fjordane; locality: Ytredalen, in the vicinity of Vadheim; verbatimElevation: 100; decimalLatitude: 61.2336; decimalLongitude: 5.8225; **Event:** samplingProtocol: T; eventDate: 31/07/2001; habitat: Litter
- **Type status:** Other material. **Occurrence:** recordNumber: 1 DN; recordedBy: MG, GM; **Location:** county: NOR-Sogn og Fjordane; locality: Kroken; verbatimElevation: 50; decimalLatitude: 61.3403; decimalLongitude: 7.3967; **Event:** samplingProtocol: T; eventDate: 19/08/2001; habitat: Soil at the base of elm trunk
- **Type status:** Other material. **Occurrence:** recordNumber: 1 ♀; recordedBy: MG; **Location:** county: NOR-Sogn og Fjordane; locality: In the vicinity of Nystølen; verbatimElevation: 725; decimalLatitude: 61.3442; decimalLongitude: 6.4664; **Event:** samplingProtocol: T; eventDate: 16/09/2001; habitat: Soil, moss, undergrowth
- **Type status:** Other material. **Occurrence:** recordNumber: 1 ♀; recordedBy: MG; **Location:** county: NOR-Sogn og Fjordane; locality: In the vicinity of Nystølen; verbatimElevation: 725; decimalLatitude: 61.3442; decimalLongitude: 6.4664; **Event:** samplingProtocol: T; eventDate: 16/09/2001; habitat: Soil, moss, undergrowth
- **Type status:** Other material. **Occurrence:** recordNumber: 1 ♀; recordedBy: MG; **Location:** county: NOR-Sogn og Fjordane; locality: Kusslia, in the vicinity of Moskog; verbatimElevation: 50; decimalLatitude: 61.4347; decimalLongitude: 5.9583; **Event:** samplingProtocol: T; eventDate: 16/09/2001; habitat: Litter
- **Type status:** Other material. **Occurrence:** recordNumber: 3 DN; recordedBy: MG; **Location:** county: NOR-Sogn og Fjordane; locality: Vikafjellet; verbatimElevation: 980; decimalLatitude: 60.9964; decimalLongitude: 6.5461; **Event:** samplingProtocol: T; eventDate: 23/09/2001; habitat: Litter
- **Type status:** Other material. **Occurrence:** recordNumber: 1 ♀; recordedBy: MG; **Location:** county: NOR-Sogn og Fjordane; locality: In the vicinity of Skjolden; verbatimElevation: 50; decimalLatitude: 61.4903; decimalLongitude: 7.5736; **Event:** samplingProtocol: U; eventDate: 08/05/2002; habitat: Litter
- **Type status:** Other material. **Occurrence:** recordNumber: 1 DN; recordedBy: MG, GM; **Location:** county: NOR-Sogn og Fjordane; locality: Jostedalen, in the vicinity of Gaupne; verbatimElevation: 50; decimalLatitude: 61.4733; decimalLongitude: 7.2547; **Event:** samplingProtocol: T; eventDate: 15/05/2005; habitat: Rot from the inside of decaying alder tree
- **Type status:** Other material. **Occurrence:** recordNumber: 4 L; recordedBy: JŁ; **Location:** county: NOR-Sogn og Fjordane; locality: In the vicinity of Skjolden; verbatimElevation: 50; decimalLatitude: 61.4972; decimalLongitude: 7.6108; **Event:** samplingProtocol: U; eventDate: 10/07/2006; habitat: Slope near road, on rocks, under moss
- **Type status:** Other material. **Occurrence:** recordNumber: 1 DN; recordedBy: MG; **Location:** county: NOR-Sogn og Fjordane; locality: Skjolden; verbatimElevation: 25; decimalLatitude: 61.4914; decimalLongitude: 7.6050; **Event:** samplingProtocol: T; eventDate: 06/09/2006; habitat: Moss, soil, humus
- **Type status:** Other material. **Occurrence:** recordNumber: 2 DN; recordedBy: MG; **Location:** county: NOR-Sogn og Fjordane; locality: In the vicinity of Skei; verbatimElevation: 200; decimalLatitude: 61.6483; decimalLongitude: 6.5200; **Event:** samplingProtocol: T; eventDate: 22/09/2006; habitat: Soil beneath rock
- **Type status:** Other material. **Occurrence:** recordNumber: 1 DN; recordedBy: AB; **Location:** county: NOR-Sogn og Fjordane; locality: Austerdalsbreen; verbatimElevation: 375; decimalLatitude: 61.5758; decimalLongitude: 6.9886; **Event:** samplingProtocol: U (L); eventDate: 03/10/2007; habitat: Postglacial valley, on stones
- **Type status:** Other material. **Occurrence:** recordNumber: 1 DN; recordedBy: MG; **Location:** county: NOR-Vestfold; locality: Tanum; verbatimElevation: 100; decimalLatitude: 59.0250; decimalLongitude: 9.9722; **Event:** samplingProtocol: T; eventDate: 25/08/2000; habitat: Litter
- **Type status:** Other material. **Occurrence:** recordNumber: 2 ♀, 1 AD; recordedBy: MG; **Location:** county: NOR-Vestfold; locality: In the vicinity of Dolven; verbatimElevation: 80; decimalLatitude: 59.0089; decimalLongitude: 9.9003; **Event:** samplingProtocol: T; eventDate: 03/10/2002; habitat: Litter
- **Type status:** Other material. **Occurrence:** recordNumber: 1 ♀, 1 ♂, 2 DN; recordedBy: MG; **Location:** county: NOR-Vestfold; locality: Larvik, Bøkeskogen; verbatimElevation: 50; decimalLatitude: 59.0592; decimalLongitude: 10.0200; **Event:** samplingProtocol: T; eventDate: 03/10/2002; habitat: Litter
- **Type status:** Other material. **Occurrence:** recordNumber: 2 ♀, 1 DN; recordedBy: MG; **Location:** county: NOR-Vestfold; locality: Between Kvelde and Rimstad; verbatimElevation: 60; decimalLatitude: 59.2217; decimalLongitude: 9.9592; **Event:** samplingProtocol: T; eventDate: 03/10/2002; habitat: Soil
- **Type status:** Other material. **Occurrence:** recordNumber: 1 DN; recordedBy: MG; **Location:** county: NOR-Vestfold; locality: Rien, in the vicinity of Odberg; verbatimElevation: 50; decimalLatitude: 59.2708; decimalLongitude: 9.9300; **Event:** samplingProtocol: T; eventDate: 03/10/2002; habitat: Litter
- **Type status:** Other material. **Occurrence:** recordNumber: 1 L; recordedBy: SMTP; **Location:** county: SWE-Skåne; locality: Skäralid, northern Lierna (=TrapID 36); verbatimElevation: 125; decimalLatitude: 56.0264; decimalLongitude: 13.2230; **Event:** samplingProtocol: M; eventDate: 22/06/2005 - 07/07/2005 (=Coll.ID 1826); habitat: Beech forest with wavy hairgrass
- **Type status:** Other material. **Occurrence:** recordNumber: 2 L; recordedBy: SMTP; **Location:** county: SWE-Västerbotten; locality: Vindelfjällen nature reserve, Tjulträsklaspen (=TrapID 46); verbatimElevation: 600; decimalLatitude: 65.9668; decimalLongitude: 16.0605; **Event:** samplingProtocol: M; eventDate: 26/07/2004 - 15/08/2004 (=Coll.ID 1201); habitat: Alpine birch wood

##### Distribution

Norway (Buitendijk 1945, Haitlinger 2000, Mąkol and Gulvik 2002), Sweden (de Geer 1778) and Finland (Karppinen 1958, Gabryś et al. 2009).

##### Notes

*Rhyncholophus nemorum* C.L. Koch, 1836 and *Leptus beroni* Fain, 1991 are junior synonyms (Mąkol et al. 2011).

#### Leptus plumosus

(Thor, 1900) [PL]

https://www.gbif.org/species/8566707

##### Distribution

Norway (Thor 1900a).

##### Notes

Regarded by Beron (2008) as *species inquirenda*.

#### Leptus rubricatus

(C.L. Koch, 1837) [PL]

https://www.gbif.org/species/9182680

##### Distribution

Sweden (Andersén 1863) and Finland (Gabryś et al. 2009).

#### Leptus sigthori

(Oudemans, 1913) [PL]

http://www.gbif.org/species/8057633

##### Distribution

Sweden (Sellnick 1949).

##### Notes

Only single record in literature (Sellnick 1949) and no recent occurrences since then. Identification questionable.

#### Leptus slivovi

Beron, 1975 [L]

http://www.gbif.org/species/6919464

##### Distribution

Norway (Southcott 1992).

#### Leptus trimaculatus

(Rossi, 1794) [PL, L]

http://www.gbif.org/species/6919458

##### Materials

- **Type status:** Other material. **Occurrence:** recordNumber: 1 ♀, 8 AD, 32 DN; recordedBy: MG; **Location:** county: NOR-Sogn og Fjordane; locality: Hafslo; verbatimElevation: 325; decimalLatitude: 61.3264; decimalLongitude: 7.2175; **Event:** samplingProtocol: T; eventDate: 15/09/2002; habitat: Soil, undergrowth
- **Type status:** Other material. **Occurrence:** recordNumber: 1 DN, 1 L; recordedBy: MG; **Location:** county: NOR-Sogn og Fjordane; locality: In the vicinity of Kroken; verbatimElevation: 50; decimalLatitude: 61.3464; decimalLongitude: 7.3981; **Event:** samplingProtocol: T; eventDate: 20/07/2003; habitat: Soil, moss
- **Type status:** Other material. **Occurrence:** recordNumber: 5 ♀, 1 ♂, 9 AD, 8 DN, 22 L; recordedBy: MG; **Location:** county: NOR-Sogn og Fjordane; locality: Hafslo; verbatimElevation: 325; decimalLatitude: 61.3264; decimalLongitude: 7.2175; **Event:** samplingProtocol: T; eventDate: 24/07/2003; habitat: Soil, undergrowth
- **Type status:** Other material. **Occurrence:** recordNumber: 1 ♀, 2 ♂, 3 AD, 17 DN, 5 L,; recordedBy: MG; **Location:** county: NOR-Sogn og Fjordane; locality: Hafslo; verbatimElevation: 325; decimalLatitude: 61.3264; decimalLongitude: 7.2175; **Event:** samplingProtocol: T; eventDate: 08/08/2003; habitat: Soil, undergrowth
- **Type status:** Other material. **Occurrence:** recordNumber: 2 ♀, 2 ♂, 1 DN, 3 AD; recordedBy: MG; **Location:** county: NOR-Sogn og Fjordane; locality: Hafslo; verbatimElevation: 325; decimalLatitude: 61.3264; decimalLongitude: 7.2175; **Event:** samplingProtocol: T; eventDate: 20/09/2003; habitat: Soil, undergrowth
- **Type status:** Other material. **Occurrence:** recordNumber: 1 ♀,1 ♂, 6 DN, 2 AD; recordedBy: MG; **Location:** county: NOR-Sogn og Fjordane; locality: Hafslo; verbatimElevation: 325; decimalLatitude: 61.3264; decimalLongitude: 7.2175; **Event:** samplingProtocol: T; eventDate: 28/09/2003; habitat: Soil, undergrowth
- **Type status:** Other material. **Occurrence:** recordNumber: 2 ♀, 4 DN; recordedBy: MG; **Location:** county: NOR-Sogn og Fjordane; locality: Between Skjolden and Luster; verbatimElevation: 25; decimalLatitude: 61.4719; decimalLongitude: 7.5461; **Event:** samplingProtocol: T; eventDate: 05/11/2005; habitat: Forest edge, soil
- **Type status:** Other material. **Occurrence:** recordNumber: 1 L; recordedBy: SMTP; **Location:** county: SWE-Södermanland; locality: Tullgarns näs, Rävsalaviken (=TrapID 30); verbatimElevation: 15; decimalLatitude: 58.9552; decimalLongitude: 17.6075; **Event:** samplingProtocol: M; eventDate: 03/07/2004 - 19/08/2004 (=Coll.ID 1055); habitat: Mixed forest next to pasture

##### Distribution

Norway (Thor 1900a), Sweden (Andersén 1863, Haitlinger 2008) and Finland (Karppinen 1958, Gabryś et al. 2009).

#### Leptus vertex

(Kramer, 1886) [PL]

http://www.gbif.org/species/6919564

##### Materials

- **Type status:** Other material. **Occurrence:** recordNumber: 1 DN; recordedBy: MG; **Location:** county: NOR-Buskerud; locality: Uvdal, in the vicinity of Rødberg,; verbatimElevation: 500; decimalLatitude: 60.2656; decimalLongitude: 8.8014; **Event:** samplingProtocol: T; eventDate: 30/09/2002; habitat: Litter, lichens, humus
- **Type status:** Other material. **Occurrence:** recordNumber: 1 DN; recordedBy: MG, SS; **Location:** county: NOR-Oppland; locality: In the vicinity of Bøvertun; verbatimElevation: 940; decimalLatitude: 61.6453; decimalLongitude: 8.1017; **Event:** samplingProtocol: T; eventDate: 17/05/2003; habitat: Soil, undergrowth
- **Type status:** Other material. **Occurrence:** recordNumber: 1 ♀, 2 ♂; recordedBy: JŁ; **Location:** county: NOR-Sogn og Fjordane; locality: Skjolden; verbatimElevation: 25; decimalLatitude: 61.4911; decimalLongitude: 7.6042; **Event:** samplingProtocol: U; eventDate: 06/07/2006; habitat: Grassy slope by the river, boulder covered with moss

##### Distribution

Norway (Thor 1900a), Sweden (Trägårdh 1910) and Finland (Gabryś et al. 2009).

#### Smarididae

Vitzthum, 1929

#### Hirstiosomatinae

Southcott, 1946

#### Hirstiosoma ampulligera

(Berlese, 1887) [PL, L]

http://www.gbif.org/species/4539748

##### Distribution

Norway (Thor 1900a).

##### Notes

Only single record in literature (Thor 1900a) and no recent occurrences since then. Identification questionable.

#### Hirstiosoma latreillei

(Grandjean, 1947) [PL, L]

http://www.gbif.org/species/4539747

##### Materials

- **Type status:** Other material. **Occurrence:** recordNumber: 1 AD, 1 DN, 1 L; recordedBy: MG, GM; **Location:** county: NOR-Sogn og Fjordane; locality: Urnes; verbatimElevation: 100; decimalLatitude: 61.2978; decimalLongitude: 7.3242; **Event:** samplingProtocol: T; eventDate: 19/08/2001; habitat: Litter
- **Type status:** Other material. **Occurrence:** recordNumber: 1 AD; recordedBy: MG; **Location:** county: NOR-Sogn og Fjordane; locality: Hella; verbatimElevation: 100; decimalLatitude: 61.2083; decimalLongitude: 6.5975; **Event:** samplingProtocol: T; eventDate: 23/09/2001; habitat: Litter
- **Type status:** Other material. **Occurrence:** recordNumber: 1 AD; recordedBy: JŁ; **Location:** county: NOR-Sogn og Fjordane; locality: Skjolden; verbatimElevation: 25; decimalLatitude: 61.4911; decimalLongitude: 7.6042; **Event:** samplingProtocol: U; eventDate: 06/07/2006; habitat: By the river, near boulder covered with moss, ant-hill
- **Type status:** Other material. **Occurrence:** recordNumber: 1 L; recordedBy: MG, JŁ; **Location:** county: NOR-Sogn og Fjordane; locality: Selja; verbatimElevation: 25; decimalLatitude: 62.0475; decimalLongitude: 5.3133; **Event:** samplingProtocol: T; eventDate: 06/08/2006; habitat: Moss on rocks
- **Type status:** Other material. **Occurrence:** recordNumber: 1 DN; recordedBy: MG, MW; **Location:** county: NOR-Sogn og Fjordane; locality: In the vicinity of Skjolden; verbatimElevation: 75; decimalLatitude: 61.4994; decimalLongitude: 7.6097; **Event:** samplingProtocol: T; eventDate: 09/12/2006; habitat: Decomposed bark at the base of birch

##### Distribution

Finland (Gabryś et al. 2009) and new for Norway.

#### Trombidioidea

#### Johnstonianidae

Thor, 1935

#### Johnstonianinae

Thor, 1935

#### Centrotrombidium schneideri

Kramer, 1896 [PL, L]

http://www.gbif.org/species/4540947

##### Distribution

Norway (Oudemans 1927).

##### Notes

Only single record in literature (Oudemans 1927) and no recent occurrences since then.

#### Diplothrombium longipalpe

(Berlese, 1887) [PL, L]

http://www.gbif.org/species/4540943

##### Materials

- **Type status:** Other material. **Occurrence:** recordNumber: 1 PL; recordedBy: PTL; **Location:** county: FIN-Southwest Finland; locality: Korppoo, Jurmo; decimalLatitude: 59.8; decimalLongitude: 21.5; **Event:** samplingProtocol: P; eventDate: 29/06/1969 - 14/08/1969; habitat: bog
- **Type status:** Other material. **Occurrence:** recordNumber: 1 PL; recordedBy: PTL; **Location:** county: FIN-Southwest Finland; locality: Parainen, Lapplahti, Kalastajakopinranta; decimalLatitude: 60.2; decimalLongitude: 21.9; **Event:** samplingProtocol: P; eventDate: 06/07/1966 - 13/10/1966; habitat: moist spruce forest with ferns
- **Type status:** Other material. **Occurrence:** recordNumber: 1 PL; recordedBy: PS. Kännö; **Location:** county: FIN-Southwest Finland; locality: Tarvasjoki, Eurankylä; decimalLatitude: 60.6; decimalLongitude: 22.7; **Event:** samplingProtocol: P; eventDate: 13/07/1969 - 03/08/1969; habitat: bog
- **Type status:** Other material. **Occurrence:** recordNumber: 1 ♀, 1 AD; recordedBy: MG; **Location:** county: NOR-Buskerud; locality: Gulsvik; verbatimElevation: 150; decimalLatitude: 60.3811; decimalLongitude: 9.6133; **Event:** samplingProtocol: T; eventDate: 07/06/2001; habitat: Litter, soil

##### Distribution

Norway (Wohltmann et al. 2004) and new for Finland.

#### Diplothrombium rackae

Wohltmann, Mąkol & Gabryś, 2004 [L]

##### Materials

- **Type status:** Other material. **Occurrence:** recordNumber: 5 L; recordedBy: MG; **Location:** county: NOR-Buskerud; locality: Gulsvik; verbatimElevation: 150; decimalLatitude: 60.3811; decimalLongitude: 9.6133; **Event:** samplingProtocol: T; eventDate: 07/06/2001; habitat: Litter, soil

##### Distribution

New for Norway.

#### Diplothrombium zbigniewi

Haitlinger, 2001 [L]

http://www.gbif.org/species/4540937

##### Materials

- **Type status:** Other material. **Occurrence:** recordNumber: 1 L; recordedBy: MG; **Location:** county: NOR-Akershus; locality: Østmarka; verbatimElevation: 250; decimalLatitude: 59.8425; decimalLongitude: 11.0338; **Event:** samplingProtocol: T; eventDate: 07/06/2002; habitat: Moss, litter, soil from small peatbog
- **Type status:** Other material. **Occurrence:** recordNumber: 1 L; recordedBy: MG; **Location:** county: NOR-Buskerud; locality: Near Hostvet, at the level of Lande; verbatimElevation: 80; decimalLatitude: 59.6083; decimalLongitude: 9.7425; **Event:** samplingProtocol: T; eventDate: 30/09/2002; habitat: Soil, moss, litter
- **Type status:** Other material. **Occurrence:** recordNumber: 3 L; recordedBy: SMTP; **Location:** county: SWE-Kalmar; locality: Bäckebo, Grytsjön nature reserve (=TrapID 1001); verbatimElevation: 80; decimalLatitude: 56.9314; decimalLongitude: 16.0855; **Event:** samplingProtocol: M; eventDate: 18/05/2006 - 15/06/2006 (=Coll.ID 1728); habitat: Old moist haymaking meadow in forest edge

##### Distribution

New for Norway and Sweden.

#### Johnstoniana errans

(Johnston, 1852) [PL, L]

http://www.gbif.org/species/4540955

##### Distribution

New for Sweden.

##### Notes

Unpublished record in material collected by Lohmander on Öland (no additional data) at the Swedish Museum of Natural History, Stockholm.

#### Johnstoniana eximia

(Berlese, 1910) [PL, L]

https://www.gbif.org/species/4540964

##### Materials

- **Type status:** Other material. **Occurrence:** recordNumber: 1 L; recordedBy: MG; **Location:** county: NOR-Akershus; locality: By the river Leira, in the vicinity of Lillestrøm; verbatimElevation: 100; decimalLatitude: 59.9642; decimalLongitude: 11.0880; **Event:** samplingProtocol: T; eventDate: 06/06/2002; habitat: By the river, litter, humus
- **Type status:** Other material. **Occurrence:** recordNumber: 7 L; recordedBy: MG; **Location:** county: NOR-Buskerud; locality: Morud; verbatimElevation: 200; decimalLatitude: 60.0603; decimalLongitude: 9.8594; **Event:** samplingProtocol: T; eventDate: 06/06/2001; habitat: Soil, litter
- **Type status:** Other material. **Occurrence:** recordNumber: 1 ♂; recordedBy: MG; **Location:** county: NOR-Vestfold; locality: In the vicinity of Nalum; verbatimElevation: 50; decimalLatitude: 58.9894; decimalLongitude: 9.9853; **Event:** samplingProtocol: T; eventDate: 03/10/2002; habitat: Soil, litter

##### Distribution

New for Norway.

#### Johnstoniana parva

Wendt, Wohltmann, Eggers & Otto, 1994 [PL, L]

http://www.gbif.org/species/4540960

##### Materials

- **Type status:** Other material. **Occurrence:** recordNumber: 6 L; recordedBy: MG; **Location:** county: NOR-Buskerud; locality: Gulsvik; verbatimElevation: 150; decimalLatitude: 60.3811; decimalLongitude: 9.6133; **Event:** samplingProtocol: T; eventDate: 07/06/2001; habitat: Litter, soil

##### Distribution

New for Norway.

#### Johnstoniana rapax

Wendt & Eggers, 1994 [PL, L]

http://www.gbif.org/species/4540967

##### Materials

- **Type status:** Other material. **Occurrence:** recordNumber: 1 ♀; recordedBy: PTL; **Location:** county: FIN-Central Finland; locality: Jyväskylä commune, Palokka; decimalLatitude: 62.3; decimalLongitude: 25.6; **Event:** eventDate: 11/08/1997; habitat: Alvajärvi lakeshore bush
- **Type status:** Other material. **Occurrence:** recordNumber: 1 ♀; recordedBy: PTL; **Location:** county: FIN-Northern Savonia; locality: Sonkajärvi, Sukeva Kalakoski; decimalLatitude: 63.8; decimalLongitude: 27.4; **Event:** eventDate: 07/08/1982; habitat: Stony shore of rapids with *Filipendula* and grass
- **Type status:** Other material. **Occurrence:** recordNumber: 1 ♀; recordedBy: PTL; **Location:** county: FIN-Southwest Finland; locality: Korppoo, Jurmo; decimalLatitude: 59.8; decimalLongitude: 21.5; **Event:** samplingProtocol: P; eventDate: 17/07/1968 - 12/10/1968; habitat: Juniper stand
- **Type status:** Other material. **Occurrence:** recordNumber: 2 ♀, 1 ad; recordedBy: PTL; **Location:** county: FIN-Southwest Finland; locality: Parainen, Mustfinnö Ippos; decimalLatitude: 60.2; decimalLongitude: 22.1; **Event:** samplingProtocol: P; eventDate: 26/08/1969 - 18/10/1969; habitat: Deciduous forest on a lake shore
- **Type status:** Other material. **Occurrence:** recordNumber: 2 ♂; recordedBy: PTL; **Location:** county: FIN-Southwest Finland; locality: Parainen, Lapplahti, Kalastajakopinranta; decimalLatitude: 60.2; decimalLongitude: 21.9; **Event:** eventDate: 18/07/1982; habitat: Bottom of wet common reed stand on seashore
- **Type status:** Other material. **Occurrence:** recordNumber: 2 AD, 1 DN; recordedBy: PTL; **Location:** county: FIN-Southwest Finland; locality: Parainen, Lapplahti; decimalLatitude: 60.29; decimalLongitude: 22.09; **Event:** samplingProtocol: P; eventDate: 06/1996 - 08/1996; habitat: Moist deciduous forest

##### Distribution

Finland (Wohltmann et al. 2004).

#### Microtrombidiidae

Thor, 1935

#### Eutrombidiinae

Thor, 1935

#### Eutrombidium frigidum

Berlese, 1910 [PL]

https://www.gbif.org/species/4540722

##### Distribution

Norway (Berlese 1910).

##### Notes

Only single record in literature (Berlese 1910) and no recent occurrences since then. Identification questionable.

#### Eutrombidium trigonum

(Hermann, 1804) [PL, L]

http://www.gbif.org/species/4540723

##### Distribution

Norway (Thor 1900b).

##### Notes

Only single record in literature (Thor 1900) and no recent occurrences since then. Identification questionable.

#### Microtrombidiinae

Thor, 1935

#### Atractothrombium fusicomum

(Berlese, 1910) [PL, L]

https://www.gbif.org/species/4540640

##### Materials

- **Type status:** Other material. **Occurrence:** recordNumber: 1 ♀; recordedBy: PTL; **Location:** county: FIN-Southwest Finland; locality: Nauvo, Sandö, W‑shore; decimalLatitude: 60.1; decimalLongitude: 22.0; **Event:** eventDate: 01/06/1992; habitat: Decaying reed

##### Distribution

New for Finland.

#### Atractothrombium sylvaticum

(C.L. Koch, 1835) [PL, L]

https://www.gbif.org/species/4540644

##### Materials

- **Type status:** Other material. **Occurrence:** recordNumber: 4 L; recordedBy: MG; **Location:** county: NOR-Hordaland; locality: Stalheimskleiva; verbatimElevation: 125; decimalLatitude: 60.8431; decimalLongitude: 6.7111; **Event:** samplingProtocol: T; eventDate: 12/08/2001; habitat: Litter
- **Type status:** Other material. **Occurrence:** recordNumber: 1 DN; recordedBy: JM; **Location:** county: NOR-Sogn og Fjordane; locality: Atløy; verbatimElevation: 100; decimalLatitude: 61.3472; decimalLongitude: 4.9589; **Event:** samplingProtocol: T; eventDate: 22/05/2001; habitat: Grass tussocks on stony beach, humic soil with herbaceous plants and moss
- **Type status:** Other material. **Occurrence:** recordNumber: 5 L; recordedBy: MG, GM; **Location:** county: NOR-Sogn og Fjordane; locality: Urnes; verbatimElevation: 100; decimalLatitude: 61.2978; decimalLongitude: 7.3242; **Event:** samplingProtocol: T; eventDate: 19/08/2001; habitat: Litter
- **Type status:** Other material. **Occurrence:** recordNumber: 1 DN; recordedBy: MG; **Location:** county: NOR-Sogn og Fjordane; locality: Kvål, in the vicinity of Grimeland; verbatimElevation: 150; decimalLatitude: 61.5122; decimalLongitude: 5.9925; **Event:** samplingProtocol: T; eventDate: 22/09/2006; habitat: Riverside with alder trees, from the inside of decaying tree trunk

##### Distribution

Norway (Berlese 1910, Mąkol and Gulvik 2002) and Sweden (Andersén 1863).

##### Notes

Microtrombidium (Enemothrombium) simulans Berlese, 1910 is a junior synonym (Gabryś et al. 2005).

#### Atractothrombium tectocervix

(Oudemans, 1903) [L]

https://www.gbif.org/species/4540645

##### Distribution

Norway (Oudemans 1912) and Sweden (Oudemans 1912, Sellnick 1958).

##### Notes

Species originally assigned to *Hydrachna* by Oudemans (1903). No recent records. Identification questionable.

#### Atractothrombium sp.

##### Materials

- **Type status:** Other material. **Occurrence:** recordNumber: 1 L; recordedBy: SMTP; **Location:** county: SWE-Värmland; locality: Ransäter, Rudstorp (=TrapID 1002); verbatimElevation: 85; decimalLatitude: 59.7730; decimalLongitude: 13.4737; **Event:** samplingProtocol: M; eventDate: 07/07/2005 - 15/07/2005 (=Coll.ID 1375); habitat: Sandy railway embankment through pasture-land

##### Notes

Potential new species for science from Sweden. Morphologically near *Atractothrombium sylvaticum* (C.L. Koch, 1835).

#### Camerotrombidium pexatum

(C.L. Koch, 1837) [PL, L]

http://www.gbif.org/species/4540682

##### Distribution

Norway (Thor 1900b, Berlese 1910, Mąkol and Gulvik 2002).

##### Notes

Microtrombium (Enemothrombium) calicigerum Berlese, 1910 is a junior synonym (Hull 1918).

#### Camerotrombidium rasum

(Berlese, 1910) [PL, L]

http://www.gbif.org/species/4540676

##### Materials

- **Type status:** Other material. **Occurrence:** recordNumber: 1 DN; recordedBy: MG, JŁ; **Location:** county: NOR-Hordaland; locality: Between Fossmarki and Stanghelle; verbatimElevation: 75; decimalLatitude: 60.5308; decimalLongitude: 5.7247; **Event:** samplingProtocol: T; eventDate: 22/08/2006; habitat: Humus
- **Type status:** Other material. **Occurrence:** recordNumber: 1 AD; recordedBy: MG; **Location:** county: NOR-Sogn og Fjordane; locality: In the vicinity of Eikefjord; verbatimElevation: 100; decimalLatitude: 61.5972; decimalLongitude: 5.4336; **Event:** samplingProtocol: T; eventDate: 13/08/2000; habitat: Litter
- **Type status:** Other material. **Occurrence:** recordNumber: 1 AD; recordedBy: MG; **Location:** county: NOR-Sogn og Fjordane; locality: Luster; verbatimElevation: 25; decimalLatitude: 61.4458; decimalLongitude: 7.4661; **Event:** samplingProtocol: T; eventDate: 11/04/2001; habitat: Soil, litter, undergrowth
- **Type status:** Other material. **Occurrence:** recordNumber: 1 AD; recordedBy: MG; **Location:** county: NOR-Sogn og Fjordane; locality: Kusslia, in the vicinity of Moskog; verbatimElevation: 50; decimalLatitude: 61.4347; decimalLongitude: 5.9583; **Event:** samplingProtocol: T; eventDate: 16/09/2001; habitat: Soil, undergrowth
- **Type status:** Other material. **Occurrence:** recordNumber: 2 AD, 2 DN; recordedBy: MG; **Location:** county: NOR-Sogn og Fjordane; locality: Between Opptun and Fortun; verbatimElevation: 600; decimalLatitude: 61.4978; decimalLongitude: 7.7492; **Event:** samplingProtocol: T; eventDate: 15/11/2005; habitat: Soil, moss, animal faeces
- **Type status:** Other material. **Occurrence:** recordNumber: 2 AD; recordedBy: IA; **Location:** county: NOR-Sogn og Fjordane; locality: Selja; verbatimElevation: 50; decimalLatitude: 62.0475; decimalLongitude: 5.3133; **Event:** samplingProtocol: T; eventDate: 03/06/2006; habitat: Soil, humus, moss
- **Type status:** Other material. **Occurrence:** recordNumber: 2 PL; recordedBy: PTL; **Location:** county: FIN-Southwest Finland; locality: Parainen, Sydmo, Ippos; decimalLatitude: 60.2; decimalLongitude: 22.1; **Event:** eventDate: 06/09/1966; habitat: In mire
- **Type status:** Other material. **Occurrence:** recordNumber: 1 AD; recordedBy: PTL; **Location:** county: FIN-Southwest Finland; locality: Parainen, Mustfinno; decimalLatitude: 60.2; decimalLongitude: 22.1; **Event:** eventDate: 10/10/1966

##### Distribution

Norway (Mąkol and Gulvik 2002) and new for Finland.

#### Camerotrombidium sigthori

Thor & Willmann, 1947 [PL]

http://www.gbif.org/species/4540681

##### Distribution

Norway (Thor 1900b).

##### Notes

*Camerotrombidium sigthori* Thor & Willmann, 1947 is a replacement name for *Ottonia purpurea* s. Thor (1900b). Only single record in literature (Thor 1900b) and no recent occurrences since then. Identification questionable.

#### Camerotrombidium vesiculosum

(Thor, 1900) [PL]

http://www.gbif.org/species/4540674

##### Distribution

Norway (Thor 1900b).

##### Notes

Only single record in literature (Thor 1900, as *Ottonia vesiculosa*) and no recent occurrences since then. Identification questionable.

#### Campylothrombium clavatum

(George, 1909) [PL, L]

http://www.gbif.org/species/4540621

##### Materials

- **Type status:** Other material. **Occurrence:** recordNumber: 1 AD; recordedBy: MG; **Location:** county: NOR-Akershus; locality: Østmarka; verbatimElevation: 250; decimalLatitude: 59.8425; decimalLongitude: 11.0338; **Event:** samplingProtocol: T; eventDate: 07/06/2002; habitat: Soil, litter
- **Type status:** Other material. **Occurrence:** recordNumber: 1 L; recordedBy: MG; **Location:** county: NOR-Buskerud; locality: Lerberg, in the vicinity of Hokksund; verbatimElevation: 100; decimalLatitude: 59.7772; decimalLongitude: 9.9244; **Event:** samplingProtocol: T; eventDate: 06/06/2001; habitat: Soil, litter
- **Type status:** Other material. **Occurrence:** recordNumber: 14 L; recordedBy: MG; **Location:** county: NOR-Buskerud; locality: Vikersund; verbatimElevation: 100; decimalLatitude: 59.9675; decimalLongitude: 9.9847; **Event:** samplingProtocol: T; eventDate: 06/06/2001; habitat: Decaying wood from tree trunk, litter and soil from trunk surroundings
- **Type status:** Other material. **Occurrence:** recordNumber: 1 ♀, 3 AD, 1 DN; recordedBy: MG; **Location:** county: NOR-Buskerud; locality: Morud; verbatimElevation: 200; decimalLatitude: 60.0603; decimalLongitude: 9.8594; **Event:** samplingProtocol: T; eventDate: 06/06/2001; habitat: Soil, litter
- **Type status:** Other material. **Occurrence:** recordNumber: 1 AD; recordedBy: MG; **Location:** county: NOR-Buskerud; locality: Gulsvik; verbatimElevation: 150; decimalLatitude: 60.3811; decimalLongitude: 9.6133; **Event:** samplingProtocol: T; eventDate: 07/06/2001; habitat: Soil, litter
- **Type status:** Other material. **Occurrence:** recordNumber: 1 AD; recordedBy: MG; **Location:** county: NOR-Hordaland; locality: In the vicinity of Stanghelle; verbatimElevation: 50; decimalLatitude: 60.5492; decimalLongitude: 5.7344; **Event:** samplingProtocol: T; eventDate: 24/06/2001; habitat: Soil, litter
- **Type status:** Other material. **Occurrence:** recordNumber: 1 DN; recordedBy: MG; **Location:** county: NOR-Vestfold; locality: Larvik, Bøkeskogen; verbatimElevation: 50; decimalLatitude: 59.0592; decimalLongitude: 10.0200; **Event:** samplingProtocol: T; eventDate: 03/10/2002; habitat: Soil, litter
- **Type status:** Other material. **Occurrence:** recordNumber: 1 L; recordedBy: SMTP; **Location:** county: SWE-Kalmar; locality: Öland (=TrapID 22); verbatimElevation: 40; decimalLatitude: 56.6167; decimalLongitude: 16.5076; **Event:** samplingProtocol: M; eventDate: 15/06/2005 - 29/06/2005 (=Coll.ID 1314); habitat: Meadow with bushes
- **Type status:** Other material. **Occurrence:** recordNumber: 2 L; recordedBy: SMTP; **Location:** county: SWE-Uppsala; locality: Biskops-Arnö (=TrapID 8); verbatimElevation: 10; decimalLatitude: 59.6721; decimalLongitude: 17.5009; **Event:** samplingProtocol: M; eventDate: 20/06/2005 - 18/07/2005 (=Coll.ID 1602); habitat: Northern beach, elm grove
- **Type status:** Other material. **Occurrence:** recordNumber: 1 DN; recordedBy: PTL; **Location:** county: FIN-North Karelia; locality: Lieksa, Koli, Ipattivaara; decimalLatitude: 63.1; decimalLongitude: 29.8; **Event:** eventDate: 01/06/1983; habitat: In litter of mixed forest (aspen, pine & spruce)
- **Type status:** Other material. **Occurrence:** recordNumber: 1 PL; recordedBy: JM; **Location:** county: FIN-North Karelia; locality: Ilomantsi, Kuikkalampi, Särkkä; decimalLatitude: 62.8; decimalLongitude: 30.9; **Event:** samplingProtocol: U; eventDate: 15/08/1997; habitat: Lakeshore bush
- **Type status:** Other material. **Occurrence:** recordNumber: 1 AD; recordedBy: PTL; **Location:** county: FIN-Southwest Finland; locality: Parainen, Mustfinno; decimalLatitude: 60.2; decimalLongitude: 22.1; **Event:** eventDate: 07/06/1966
- **Type status:** Other material. **Occurrence:** recordNumber: 1 DN; recordedBy: PTL; **Location:** county: FIN-Southwest Finland; locality: Parainen, Sydmo, Ippos; decimalLatitude: 60.2; decimalLongitude: 22.1; **Event:** eventDate: 13/06/1966
- **Type status:** Other material. **Occurrence:** recordNumber: 2 ♀; recordedBy: PTL; **Location:** county: FIN-Southwest Finland; locality: Parainen, Sydmo, Ippos; decimalLatitude: 60.2; decimalLongitude: 22.1; **Event:** eventDate: 14/06/1966
- **Type status:** Other material. **Occurrence:** recordNumber: 1 ♀, 1 PL; recordedBy: PTL; **Location:** county: FIN-Southwest Finland; locality: Parainen, Mustfinno; decimalLatitude: 60.2; decimalLongitude: 21.9; **Event:** eventDate: 10/10/1966
- **Type status:** Other material. **Occurrence:** recordNumber: 1 ♀, 1 ♂, 3 AD, 4 PL; recordedBy: PTL; **Location:** county: FIN-Southwest Finland; locality: Parainen, Lapplahti; decimalLatitude: 60.29; decimalLongitude: 22.09; **Event:** samplingProtocol: P; eventDate: 06/1967 - 08/1967; habitat: Moist deciduous forest
- **Type status:** Other material. **Occurrence:** recordNumber: 1 AD; recordedBy: PTL; **Location:** county: FIN-Southwest Finland; locality: Parainen, Kurckas; decimalLatitude: 60.2; decimalLongitude: 22.1; **Event:** samplingProtocol: P; eventDate: 17/05/1968 - 05/07/1968; habitat: Meadow in spruce forest
- **Type status:** Other material. **Occurrence:** recordNumber: 1 ♂, 8 AD; recordedBy: SK; **Location:** county: FIN-Southwest Finland; locality: Kaarina, Karpanmäki; decimalLatitude: 60.3; decimalLongitude: 22.3; **Event:** samplingProtocol: P; eventDate: 09/05/1989 - 13/06/1989; habitat: Oak forest
- **Type status:** Other material. **Occurrence:** recordNumber: 1 AD; recordedBy: SK; **Location:** county: FIN-Southwest Finland; locality: Kaarina, Kuusisto; decimalLatitude: 60.3; decimalLongitude: 22.3; **Event:** samplingProtocol: P; eventDate: 15/07/1989 - 23/09/1989; habitat: Oak forest

##### Distribution

New for Norway, Sweden and Finland.

#### Campylothrombium striaticeps

(Oudemans, 1904) [L]

##### Distribution

Sweden (Sellnick 1958).

##### Notes

Only single record in literature (Sellnick 1958) and no recent occurrences since then. Identification questionable.

#### Dactylothrombium pulcherrimum

(Haller, 1882) [PL, L]

http://www.gbif.org/species/4540615

##### Materials

- **Type status:** Other material. **Occurrence:** recordNumber: 1 AD; recordedBy: MG; **Location:** county: NOR-Akershus; locality: By the river Leira, in the vicinity of Lillestrøm; verbatimElevation: 100; decimalLatitude: 59.9642; decimalLongitude: 11.0880; **Event:** samplingProtocol: T; eventDate: 06/06/2002; habitat: By the river, litter, humus
- **Type status:** Other material. **Occurrence:** recordNumber: 2 AD, 1 ♀; recordedBy: MG; **Location:** county: NOR-Akershus; locality: Østmarka; verbatimElevation: 250; decimalLatitude: 59.8425; decimalLongitude: 11.0338; **Event:** samplingProtocol: T; eventDate: 07/06/2002; habitat: Litter, soil, rot from the inside of decaying tree trunk
- **Type status:** Other material. **Occurrence:** recordNumber: 1 AD; recordedBy: MG; **Location:** county: NOR-Buskerud; locality: Between Lindelia and Sønsteby, shore of lake Krøderen; verbatimElevation: 150; decimalLatitude: 60.3392; decimalLongitude: 9.6489; **Event:** samplingProtocol: T; eventDate: 26/08/2000; habitat: Shore of lake, moss, litter, soil
- **Type status:** Other material. **Occurrence:** recordNumber: 2 ♀, 1 AD, 2 DN; recordedBy: MG; **Location:** county: NOR-Buskerud; locality: Vikersund; verbatimElevation: 100; decimalLatitude: 59.9675; decimalLongitude: 9.9847; **Event:** samplingProtocol: T; eventDate: 06/06/2001; habitat: Rot from tree trunk, litter and soil from trunk surroundings
- **Type status:** Other material. **Occurrence:** recordNumber: 1 ♀, 10 AD, 2 L; recordedBy: MG; **Location:** county: NOR-Buskerud; locality: Morud; verbatimElevation: 200; decimalLatitude: 60.0603; decimalLongitude: 9.8594; **Event:** samplingProtocol: T; eventDate: 06/06/2001; habitat: Soil, litter
- **Type status:** Other material. **Occurrence:** recordNumber: 3 AD, 1 DN; recordedBy: MG; **Location:** county: NOR-Buskerud; locality: Gulsvik; verbatimElevation: 150; decimalLatitude: 60.3811; decimalLongitude: 9.6133; **Event:** samplingProtocol: T; eventDate: 07/06/2001; habitat: Litter, soil
- **Type status:** Other material. **Occurrence:** recordNumber: 1 AD; recordedBy: MG; **Location:** county: NOR-Buskerud; locality: Hjelmen; verbatimElevation: 600; decimalLatitude: 60.8081; decimalLongitude: 8.6772; **Event:** samplingProtocol: T; eventDate: 07/06/2001; habitat: Litter, soil
- **Type status:** Other material. **Occurrence:** recordNumber: 1 ♀, 3 DN; recordedBy: MG; **Location:** county: NOR-Hordaland; locality: In the vicinity of Stanghelle; verbatimElevation: 50; decimalLatitude: 60.5492; decimalLongitude: 5.7344; **Event:** samplingProtocol: T; eventDate: 24/06/2001; habitat: Litter, soil
- **Type status:** Other material. **Occurrence:** recordNumber: 3 AD, 2 L; recordedBy: MG; **Location:** county: NOR-Hordaland; locality: Between Bulken and Rekve; verbatimElevation: 60; decimalLatitude: 60.6275; decimalLongitude: 6.2961; **Event:** samplingProtocol: T; eventDate: 24/06/2001; habitat: Moss and humus at the base of tree trunk
- **Type status:** Other material. **Occurrence:** recordNumber: 3♀, 1 L; recordedBy: MG; **Location:** county: NOR-Hordaland; locality: In the vicinity of Kolstallen and Bergen; verbatimElevation: 50; decimalLatitude: 60.4411; decimalLongitude: 5.4511; **Event:** samplingProtocol: T; eventDate: 04/07/2001; habitat: Soil, undergrowth, rot from decaying tree trunk
- **Type status:** Other material. **Occurrence:** recordNumber: 1 DN; recordedBy: MG; **Location:** county: NOR-Hordaland; locality: Blinde; verbatimElevation: 100; decimalLatitude: 60.4639; decimalLongitude: 5.3786; **Event:** samplingProtocol: T; eventDate: 04/07/2001; habitat: Moss, litter, soil
- **Type status:** Other material. **Occurrence:** recordNumber: 2 DN, 1 ♀; recordedBy: MG; **Location:** county: NOR-Hordaland; locality: Haukeland, in the vicinity of Bergen; verbatimElevation: 100; decimalLatitude: 60.3528; decimalLongitude: 5.4478; **Event:** samplingProtocol: T; eventDate: 05/07/2004; habitat: Soil, humus, litter
- **Type status:** Other material. **Occurrence:** recordNumber: 2 DN; recordedBy: MG; **Location:** county: NOR-Hordaland; locality: Dale; verbatimElevation: 50; decimalLatitude: 60.5881; decimalLongitude: 5.8178; **Event:** samplingProtocol: T; eventDate: 5/07/2004; habitat: Litter, humus
- **Type status:** Other material. **Occurrence:** recordNumber: 1 ♀; recordedBy: MG; **Location:** county: NOR-Hordaland; locality: Dale; verbatimElevation: 50; decimalLatitude: 60.5881; decimalLongitude: 5.8178; **Event:** samplingProtocol: T; eventDate: 25/06/2005; habitat: Litter, humus
- **Type status:** Other material. **Occurrence:** recordNumber: 3 AD, 1 DN, 7 L,; recordedBy: MG; **Location:** county: NOR-Hordaland; locality: Hola; verbatimElevation: 570; decimalLatitude: 60.9019; decimalLongitude: 6.4836; **Event:** samplingProtocol: T; eventDate: 25/06/2005; habitat: Litter, moss
- **Type status:** Other material. **Occurrence:** recordNumber: 1 ♀, 2 AD; recordedBy: MG; **Location:** county: NOR-Sogn og Fjordane; locality: Kaupanger; verbatimElevation: 200; decimalLatitude: 61.1942; decimalLongitude: 7.2192; **Event:** samplingProtocol: T; eventDate: 15/08/2000; habitat: Rot from tree trunk
- **Type status:** Other material. **Occurrence:** recordNumber: 2 AD; recordedBy: MG; **Location:** county: NOR-Sogn og Fjordane; locality: In the vicinity of Sogndal; verbatimElevation: 180; decimalLatitude: 61.2042; decimalLongitude: 7.1983; **Event:** samplingProtocol: T; eventDate: 15/08/2000; habitat: Soil, humus
- **Type status:** Other material. **Occurrence:** recordNumber: 1 DN; recordedBy: MG; **Location:** county: NOR-Sogn og Fjordane; locality: Mo, in the vicinity of Moskog; verbatimElevation: 100; decimalLatitude: 61.4364; decimalLongitude: 5.9856; **Event:** samplingProtocol: T; eventDate: 11/10/2000; habitat: Moss, litter, soil
- **Type status:** Other material. **Occurrence:** recordNumber: 1 AD; recordedBy: MG; **Location:** county: NOR-Sogn og Fjordane; locality: Førde; verbatimElevation: 100; decimalLatitude: 61.4550; decimalLongitude: 5.8581; **Event:** samplingProtocol: T; eventDate: 11/10/2000; habitat: Moss, soil, undergrowth, beneath rock wall
- **Type status:** Other material. **Occurrence:** recordNumber: 3 AD, 1 DN; recordedBy: MG; **Location:** county: NOR-Sogn og Fjordane; locality: In the vicinity of Bøyabreen; verbatimElevation: 150; decimalLatitude: 61.4811; decimalLongitude: 6.7419; **Event:** samplingProtocol: T; eventDate: 11/10/2000; habitat: Moss, litter, soil
- **Type status:** Other material. **Occurrence:** recordNumber: 1 AD; recordedBy: MG; **Location:** county: NOR-Sogn og Fjordane; locality: Near Kjøsnesfjorden, in the vicinity of Kjøsnes; verbatimElevation: 250; decimalLatitude: 61.5447; decimalLongitude: 6.5106; **Event:** samplingProtocol: T; eventDate: 11/10/2000; habitat: Moss, litter, soil
- **Type status:** Other material. **Occurrence:** recordNumber: 1 L; recordedBy: JM; **Location:** county: NOR-Sogn og Fjordane; locality: Atløy; verbatimElevation: 100; decimalLatitude: 61.3472; decimalLongitude: 4.9589; **Event:** samplingProtocol: T; eventDate: 22/05/2001; habitat: Grass tussocks on stony beach, humus, undergrowth, moss
- **Type status:** Other material. **Occurrence:** recordNumber: 2 AD, 1 DN; recordedBy: MG, JM; **Location:** county: NOR-Sogn og Fjordane; locality: Hafslo; verbatimElevation: 300; decimalLatitude: 61.3314; decimalLongitude: 7.2144; **Event:** samplingProtocol: T; eventDate: 25/05/2001; habitat: Moss, litter, soil
- **Type status:** Other material. **Occurrence:** recordNumber: 1 DN; recordedBy: MG; **Location:** county: NOR-Sogn og Fjordane; locality: Osen; verbatimElevation: 1 120; decimalLatitude: 61.0078; decimalLongitude: 8.1094; **Event:** samplingProtocol: T; eventDate: 07/06/2001; habitat: Moss, litter, soil
- **Type status:** Other material. **Occurrence:** recordNumber: 1 AD; recordedBy: MG; **Location:** county: NOR-Sogn og Fjordane; locality: Hønjum, in the vicinity of Bjørkum; verbatimElevation: 100; decimalLatitude: 61.0583; decimalLongitude: 7.6617; **Event:** samplingProtocol: T; eventDate: 07/06/2001; habitat: Moss, soil
- **Type status:** Other material. **Occurrence:** recordNumber: 2 AD, 19 DN, 1 L; recordedBy: MG; **Location:** county: NOR-Sogn og Fjordane; locality: Lærdalsøyri; verbatimElevation: 100; decimalLatitude: 61.0972; decimalLongitude: 7.4911; **Event:** samplingProtocol: T; eventDate: 07/06/2001; habitat: Litter, soil
- **Type status:** Other material. **Occurrence:** recordNumber: 1 DN; recordedBy: MG, GM; **Location:** county: NOR-Sogn og Fjordane; locality: Balestrand; verbatimElevation: 100; decimalLatitude: 61.2081; decimalLongitude: 6.5281; **Event:** samplingProtocol: T; eventDate: 31/07/2001; habitat: Moss at the base of decaying alder trunk
- **Type status:** Other material. **Occurrence:** recordNumber: 1 DN; recordedBy: MG, GM; **Location:** county: NOR-Sogn og Fjordane; locality: In the vicinity of Urnes; verbatimElevation: 50; decimalLatitude: 61.3114; decimalLongitude: 7.3556; **Event:** samplingProtocol: T; eventDate: 19/08/2001; habitat: Moss, soil, humus
- **Type status:** Other material. **Occurrence:** recordNumber: 1 DN; recordedBy: MG; **Location:** county: NOR-Sogn og Fjordane; locality: In the vicinity of Bøyabreen; verbatimElevation: 175; decimalLatitude: 61.4817; decimalLongitude: 6.7406; **Event:** samplingProtocol: T; eventDate: 16/09/2001; habitat: Soil, undergrowth
- **Type status:** Other material. **Occurrence:** recordNumber: 1 DN; recordedBy: MG; **Location:** county: NOR-Sogn og Fjordane; locality: Vikafjellet; verbatimElevation: 980; decimalLatitude: 60.9964; decimalLongitude: 6.5461; **Event:** samplingProtocol: T; eventDate: 23/09/2001; habitat: Soil, humus, moss
- **Type status:** Other material. **Occurrence:** recordNumber: 3 AD; recordedBy: MG; **Location:** county: NOR-Sogn og Fjordane; locality: Vikafjellet; verbatimElevation: 950; decimalLatitude: 60.9917; decimalLongitude: 6.5403; **Event:** samplingProtocol: T; eventDate: 23/09/2001; habitat: Moss, soil, undergrowth
- **Type status:** Other material. **Occurrence:** recordNumber: 2 AD, 23 L; recordedBy: MG; **Location:** county: NOR-Sogn og Fjordane; locality: In the vicinity of Skjolden; verbatimElevation: 50; decimalLatitude: 61.4903; decimalLongitude: 7.5736; **Event:** samplingProtocol: T; eventDate: 08/05/2002; habitat: Litter, soil, moss, rot
- **Type status:** Other material. **Occurrence:** recordNumber: 1 ♀, 1 AD; recordedBy: MG; **Location:** county: NOR-Sogn og Fjordane; locality: In the vicinity of Opptun; verbatimElevation: 600; decimalLatitude: 61.4978; decimalLongitude: 7.7492; **Event:** samplingProtocol: T; eventDate: 10/05/2002; habitat: Rot, litter, soil
- **Type status:** Other material. **Occurrence:** recordNumber: 1 AD; recordedBy: MG; **Location:** county: NOR-Sogn og Fjordane; locality: Hafslo; verbatimElevation: 325; decimalLatitude: 61.3264; decimalLongitude: 7.2175; **Event:** samplingProtocol: T; eventDate: 15/09/2002; habitat: Soil, undergrowth
- **Type status:** Other material. **Occurrence:** recordNumber: 1 AD; recordedBy: MG, GM; **Location:** county: NOR-Sogn og Fjordane; locality: Mørkri, in the vicinity of Skjolden; verbatimElevation: 150; decimalLatitude: 61.5567; decimalLongitude: 7.6208; **Event:** samplingProtocol: T; eventDate: 20/04/2003; habitat: Soil, humus
- **Type status:** Other material. **Occurrence:** recordNumber: 2 DN; recordedBy: MG, MK; **Location:** county: NOR-Sogn og Fjordane; locality: Hafslovatnet, in the vicinity of Hafslo; verbatimElevation: 200; decimalLatitude: 61.2917; decimalLongitude: 7.1647; **Event:** samplingProtocol: T; eventDate: 04/08/2003; habitat: Near lake, moss, soil
- **Type status:** Other material. **Occurrence:** recordNumber: 3 L,; recordedBy: MG, GM; **Location:** county: NOR-Sogn og Fjordane; locality: In the vicinity of Opptun; verbatimElevation: 600; decimalLatitude: 61.4978; decimalLongitude: 7.7492; **Event:** samplingProtocol: T; eventDate: 08/05/2005; habitat: Soil, litter
- **Type status:** Other material. **Occurrence:** recordNumber: 7 AD, 29 L; recordedBy: MG, GM; **Location:** county: NOR-Sogn og Fjordane; locality: Jostedalen, in the vicinity of Gaupne; verbatimElevation: 50; decimalLatitude: 61.4733; decimalLongitude: 7.2547; **Event:** samplingProtocol: T; eventDate: 15/05/2005; habitat: Soil, humus at the base of alder tree
- **Type status:** Other material. **Occurrence:** recordNumber: 5 AD, 1 DN, 81 L; recordedBy: MG, GM; **Location:** county: NOR-Sogn og Fjordane; locality: Jostedalen, in the vicinity of Gaupne; verbatimElevation: 50; decimalLatitude: 61.4917; decimalLongitude: 7.2681; **Event:** samplingProtocol: T; eventDate: 15/05/2005; habitat: Rot from alder tree trunk, humus
- **Type status:** Other material. **Occurrence:** recordNumber: 1 DN; recordedBy: MG, GM; **Location:** county: NOR-Sogn og Fjordane; locality: Jostedalen, in the vicinity of Gaupne; verbatimElevation: 100; decimalLatitude: 61.5039; decimalLongitude: 7.2750; **Event:** samplingProtocol: T; eventDate: 15/05/2005; habitat: Rot, moss
- **Type status:** Other material. **Occurrence:** recordNumber: 3 AD; recordedBy: MG; **Location:** county: NOR-Sogn og Fjordane; locality: Skjolden; verbatimElevation: 25; decimalLatitude: 61.4914; decimalLongitude: 7.6000; **Event:** samplingProtocol: T; eventDate: 05/11/2005; habitat: Soil, litter
- **Type status:** Other material. **Occurrence:** recordNumber: 1 DN; recordedBy: MG; **Location:** county: NOR-Sogn og Fjordane; locality: Between Opptun and Fortun; verbatimElevation: 600; decimalLatitude: 61.4978; decimalLongitude: 7.7492; **Event:** samplingProtocol: T; eventDate: 15/11/2005; habitat: Soil, litter
- **Type status:** Other material. **Occurrence:** recordNumber: 4 AD; recordedBy: MG; **Location:** county: NOR-Sogn og Fjordane; locality: Between Skjolden and Fortun; verbatimElevation: 25; decimalLatitude: 61.4878; decimalLongitude: 7.6594; **Event:** samplingProtocol: T; eventDate: 25/11/2005; habitat: Moss and soil
- **Type status:** Other material. **Occurrence:** recordNumber: 2 AD, 1 DN; recordedBy: MG; **Location:** county: NOR-Sogn og Fjordane; locality: Leirdal, in the vicinity of Gaupne; verbatimElevation: 75; decimalLatitude: 61.4628; decimalLongitude: 7.2497; **Event:** samplingProtocol: T; eventDate: 25/12/2006; habitat: Decomposed wood, humus
- **Type status:** Other material. **Occurrence:** recordNumber: 1 ♀, 1 AD; recordedBy: MG; **Location:** county: NOR-Vestfold; locality: In the vicinity of Nalum; verbatimElevation: 50; decimalLatitude: 58.9894; decimalLongitude: 9.9853; **Event:** samplingProtocol: T; eventDate: 03/10/2002; habitat: Rot from the inside of decaying tree trunk
- **Type status:** Other material. **Occurrence:** recordNumber: 3 AD; recordedBy: MG; **Location:** county: NOR-Vestfold; locality: Larvik, Bøkeskogen; verbatimElevation: 50; decimalLatitude: 59.0592; decimalLongitude: 10.0200; **Event:** samplingProtocol: T; eventDate: 03/10/2002; habitat: Litter, soil
- **Type status:** Other material. **Occurrence:** recordNumber: 2 ♀, 2 AD; recordedBy: MG; **Location:** county: NOR-Vestfold; locality: Between Larvik and Kvelde; verbatimElevation: 25; decimalLatitude: 59.1525; decimalLongitude: 10.0244; **Event:** samplingProtocol: T; eventDate: 03/10/2002; habitat: Litter, soil
- **Type status:** Other material. **Occurrence:** recordNumber: 1 ♀; recordedBy: MG; **Location:** county: NOR-Vestfold; locality: Between Kvelde and Rimstad; verbatimElevation: 60; decimalLatitude: 59.2217; decimalLongitude: 9.9592; **Event:** samplingProtocol: T; eventDate: 03/10/2002; habitat: Litter
- **Type status:** Other material. **Occurrence:** recordNumber: 2 ♀, 4 AD; recordedBy: MG; **Location:** county: NOR-Vestfold; locality: Rien, in the vicinity of Odberg; verbatimElevation: 50; decimalLatitude: 59.2708; decimalLongitude: 9.9300; **Event:** samplingProtocol: T; eventDate: 03/10/2002; habitat: Litter, soil, decaying wood from tree trunk

##### Distribution

Norway (Mąkol and Gulvik 2002).

#### Dactylothrombium sp.

##### Materials

- **Type status:** Other material. **Occurrence:** recordNumber: 10 L; recordedBy: MG, AK; **Location:** county: NOR-Sogn og Fjordane; locality: Sognefjellet; verbatimElevation: 1 300; decimalLatitude: 61.5478; decimalLongitude: 7.8697; **Event:** samplingProtocol: T; eventDate: 22/06/2000; habitat: Soil, undergrowth

##### Notes

Potential new species for science from Norway.

#### Dimorphothrombium geographicum

(Berlese, 1910) [PL]

http://www.gbif.org/species/4540592

##### Distribution

Norway (Berlese 1910).

##### Notes

Only single record in literature (Berlese 1910) and no recent occurrences since then. Identification questionable.

#### Dimorphothrombium plancum

(C.L. Koch, 1837) [PL]

http://www.gbif.org/species/4540584

##### Distribution

Norway (Thor 1900b).

##### Notes

Only single record in literature (Thor 1900b, as *Ottonia planca*) and no recent occurrences since then. Identification questionable.

#### Echinothrombium rhodinum

(C. L. Koch, 1837) [PL, L]

http://www.gbif.org/species/4540597

##### Materials

- **Type status:** Other material. **Occurrence:** recordNumber: 1 L; recordedBy: SMTP; **Location:** county: SWE-Uppsala; locality: Biskops-Arnö (=TrapID 8); verbatimElevation: 10; decimalLatitude: 59.6721; decimalLongitude: 17.5009; **Event:** samplingProtocol: M; eventDate: 20/06/2005 - 18/07/2005 (=Coll.ID 1602); habitat: Northern beach, elm grove
- **Type status:** Other material. **Occurrence:** recordNumber: 1 ♀, 3 DN; recordedBy: JM; **Location:** county: FIN-North Karelia; locality: Tohmajärvi, Kemie, Kirkkoniemi; decimalLatitude: 62.8; decimalLongitude: 30.9; **Event:** samplingProtocol: U; eventDate: 15/08/1997; habitat: Deciduous forest with *Rhamnus* & alder
- **Type status:** Other material. **Occurrence:** recordNumber: 6 PL; recordedBy: PTL; **Location:** county: FIN-Northern Ostrobothnia; locality: Oulunsalo, Varjakka; decimalLatitude: 65.0; decimalLongitude: 25.3; **Event:** eventDate: 13/06/1987; habitat: Seashore meadow
- **Type status:** Other material. **Occurrence:** recordNumber: 1 DN; recordedBy: PTL; **Location:** county: FIN-Southwest Finland; locality: Korppo, Österskär, Brännkläpp; decimalLatitude: 59.9; decimalLongitude: 21.1; **Event:** eventDate: 21/06/1994; habitat: Litter of maritime deciduous forest
- **Type status:** Other material. **Occurrence:** recordNumber: 1 ♀; recordedBy: PTL; **Location:** county: FIN-Southwest Finland; locality: Rymättylä, Alakylä, Isoluoto; decimalLatitude: 60.30; decimalLongitude: 21.97; **Event:** eventDate: 04/09/1997; habitat: Litter of alder and decaying reed on seashore

##### Distribution

New for Sweden and Finland.

#### Echinothrombium spinosum

(Canestrini, 1885) [PL, L]

http://www.gbif.org/species/4540599

##### Materials

- **Type status:** Other material. **Occurrence:** recordNumber: 2 ♀; recordedBy: MG; **Location:** county: NOR-Akershus; locality: By the river Leira, in the vicinity of Lillestrøm; verbatimElevation: 100; decimalLatitude: 59.9658; decimalLongitude: 11.0947; **Event:** samplingProtocol: T; eventDate: 06/06/2002; habitat: By the river, soil
- **Type status:** Other material. **Occurrence:** recordNumber: 20 L; recordedBy: MG; **Location:** county: NOR-Buskerud; locality: Lerberg, in the vicinity of Hokksund; verbatimElevation: 100; decimalLatitude: 59.7772; decimalLongitude: 9.9244; **Event:** samplingProtocol: T; eventDate: 06/06/2001; habitat: Litter, soil
- **Type status:** Other material. **Occurrence:** recordNumber: 12 L; recordedBy: MG; **Location:** county: NOR-Buskerud; locality: Vikersund; verbatimElevation: 100; decimalLatitude: 59.9675; decimalLongitude: 9.9847; **Event:** samplingProtocol: T; eventDate: 06/06/2001; habitat: Decaying wood from tree trunk, litter and soil from trunk surroundings
- **Type status:** Other material. **Occurrence:** recordNumber: 1 ♀; recordedBy: MG; **Location:** county: NOR-Hordaland; locality: In the vicinity of Bulken; verbatimElevation: 75; decimalLatitude: 60.6314; decimalLongitude: 6.2744; **Event:** samplingProtocol: T; eventDate: 25/06/2005; habitat: Moss and rot at decaying trunk
- **Type status:** Other material. **Occurrence:** recordNumber: 1 AD; recordedBy: MG; **Location:** county: NOR-Sogn og Fjordane; locality: In the vicinity of Eikefjord; verbatimElevation: 100; decimalLatitude: 61.5972; decimalLongitude: 5.4336; **Event:** samplingProtocol: T; eventDate: 13/08/2000; habitat: Litter
- **Type status:** Other material. **Occurrence:** recordNumber: 1 DN; recordedBy: MG, GM; **Location:** county: NOR-Sogn og Fjordane; locality: Jostedalen, in the vicinity of Gaupne; verbatimElevation: 50; decimalLatitude: 61.4733; decimalLongitude: 7.2547; **Event:** samplingProtocol: T; eventDate: 20/09/2000; habitat: Rot of pine tree
- **Type status:** Other material. **Occurrence:** recordNumber: 1 ♀, 1 DN; recordedBy: MG; **Location:** county: NOR-Sogn og Fjordane; locality: Mo, in the vicinity of Moskog; verbatimElevation: 100; decimalLatitude: 61.4364; decimalLongitude: 5.9856; **Event:** samplingProtocol: T; eventDate: 11/10/2000; habitat: Moss and rot from tree trunk
- **Type status:** Other material. **Occurrence:** recordNumber: 1 DN; recordedBy: MG; **Location:** county: NOR-Sogn og Fjordane; locality: Near Kjøsnesfjorden, in the vicinity of Kjøsnes; verbatimElevation: 250; decimalLatitude: 61.5447; decimalLongitude: 6.5106; **Event:** samplingProtocol: T; eventDate: 11/10/2000; habitat: Litter
- **Type status:** Other material. **Occurrence:** recordNumber: 1 DN; recordedBy: MG; **Location:** county: NOR-Sogn og Fjordane; locality: Anda; verbatimElevation: 100; decimalLatitude: 61.8461; decimalLongitude: 6.0817; **Event:** samplingProtocol: T; eventDate: 02/02/2001; habitat: Rot from decaying birch tree trunk
- **Type status:** Other material. **Occurrence:** recordNumber: 1 AD; recordedBy: MG, GM; **Location:** county: NOR-Sogn og Fjordane; locality: Ytredalen, in the vicinity of Vadheim; verbatimElevation: 100; decimalLatitude: 61.2336; decimalLongitude: 5.8225; **Event:** samplingProtocol: T; eventDate: 31/07/2001; habitat: Litter
- **Type status:** Other material. **Occurrence:** recordNumber: 2 DN; recordedBy: MG; **Location:** county: NOR-Sogn og Fjordane; locality: Kusslia, in the vicinity of Moskog; verbatimElevation: 50; decimalLatitude: 61.4347; decimalLongitude: 5.9583; **Event:** samplingProtocol: T; eventDate: 16/09/2001; habitat: Moss and vegetation on decaying trunk of elm tree
- **Type status:** Other material. **Occurrence:** recordNumber: 1 ♀, 1 DN; recordedBy: MG; **Location:** county: NOR-Sogn og Fjordane; locality: Hella; verbatimElevation: 100; decimalLatitude: 61.2083; decimalLongitude: 6.5975; **Event:** samplingProtocol: T; eventDate: 23/09/2001; habitat: Litter
- **Type status:** Other material. **Occurrence:** recordNumber: 1 AD; recordedBy: MG, SSM; **Location:** county: NOR-Sogn og Fjordane; locality: Svanøy; verbatimElevation: 100; decimalLatitude: 61.4828; decimalLongitude: 5.0972; **Event:** samplingProtocol: T; eventDate: 11/11/2001; habitat: Bark, decaying wood and humus from tree trunk
- **Type status:** Other material. **Occurrence:** recordNumber: 3 ♀; recordedBy: MG; **Location:** county: NOR-Sogn og Fjordane; locality: In the vicinity of Skjolden; verbatimElevation: 50; decimalLatitude: 61.4903; decimalLongitude: 7.5736; **Event:** samplingProtocol: T; eventDate: 08/05/2002; habitat: Litter, moss, rot from the inside of decaying tree trunk
- **Type status:** Other material. **Occurrence:** recordNumber: 4 DN; recordedBy: MG, GM; **Location:** county: NOR-Sogn og Fjordane; locality: Jostedalen, in the vicinity of Gaupne; verbatimElevation: 50; decimalLatitude: 61.4733; decimalLongitude: 7.2547; **Event:** samplingProtocol: T; eventDate: 15/05/2005; habitat: Soil, humus, at the base of alder tree
- **Type status:** Other material. **Occurrence:** recordNumber: 1 ♀; recordedBy: MG, JŁ; **Location:** county: NOR-Sogn og Fjordane; locality: In the vicinity of kjolden; verbatimElevation: 25; decimalLatitude: 61.4950; decimalLongitude: 7.5867; **Event:** samplingProtocol: T; eventDate: 20/08/2006; habitat: Litter, soil
- **Type status:** Other material. **Occurrence:** recordNumber: 1 DN; recordedBy: MG, JŁ; **Location:** county: NOR-Sogn og Fjordane; locality: Fortunsdalen, in the vicinity of Fortun; verbatimElevation: 150; decimalLatitude: 61.5222; decimalLongitude: 7.7128; **Event:** samplingProtocol: T; eventDate: 20/08/2006; habitat: Soil, humus
- **Type status:** Other material. **Occurrence:** recordNumber: 2 DN; recordedBy: MG, MW; **Location:** county: NOR-Sogn og Fjordane; locality: In the vicinity of Skjolden; verbatimElevation: 75; decimalLatitude: 61.4994; decimalLongitude: 7.6097; **Event:** samplingProtocol: T; eventDate: 09/12/2006; habitat: House garden, soil and litter on stones and under shrub
- **Type status:** Other material. **Occurrence:** recordNumber: 1 ♀; recordedBy: MG; **Location:** county: NOR-Sogn og Fjordane; locality: Leirdal, in the vicinity of Gaupne; verbatimElevation: 75; decimalLatitude: 61.4628; decimalLongitude: 7.2497; **Event:** samplingProtocol: T; eventDate: 25/12/2006; habitat: Decomposed wood, humus
- **Type status:** Other material. **Occurrence:** recordNumber: 3 ♀, 1 DN; recordedBy: JŁ; **Location:** county: NOR-Sogn og Fjordane; locality: In the vicinity of Skjolden; verbatimElevation: 75; decimalLatitude: 61.4992; decimalLongitude: 7.6094; **Event:** samplingProtocol: U; eventDate: 08/09/2007; habitat: Slope by the road, very moist grass, moss, soil
- **Type status:** Other material. **Occurrence:** recordNumber: 1 ♀; recordedBy: JŁ; **Location:** county: NOR-Sogn og Fjordane; locality: Skjolden; verbatimElevation: 25; decimalLatitude: 61.4919; decimalLongitude: 7.6058; **Event:** samplingProtocol: U; eventDate: 10/09/2007; habitat: Meadow, close to river and forest, relatively moist soil, grass and moss
- **Type status:** Other material. **Occurrence:** recordNumber: 1 ♀; recordedBy: JŁ; **Location:** county: NOR-Sogn og Fjordane; locality: Skjolden; verbatimElevation: 25; decimalLatitude: 61.4928; decimalLongitude: 7.6069; **Event:** samplingProtocol: U; eventDate: 17/09/2007; habitat: Larch forest close to road, moist litter with rot
- **Type status:** Other material. **Occurrence:** recordNumber: 1 ♀; recordedBy: JŁ; **Location:** county: NOR-Sogn og Fjordane; locality: In the vicinity of Skjolden; verbatimElevation: 75; decimalLatitude: 61.4992; decimalLongitude: 7.6094; **Event:** samplingProtocol: U; eventDate: 17/09/2007; habitat: Slope by the road, very moist grass, moss, soil
- **Type status:** Other material. **Occurrence:** recordNumber: 1 ♀; recordedBy: JŁ; **Location:** county: NOR-Sogn og Fjordane; locality: In the vicinity of Skjolden; verbatimElevation: 75; decimalLatitude: 61.4992; decimalLongitude: 7.6094; **Event:** samplingProtocol: U; eventDate: 05/10/2007; habitat: Slope by the road, very moist grass, moss, soil
- **Type status:** Other material. **Occurrence:** recordNumber: 3 DN; recordedBy: MG; **Location:** county: NOR-Vestfold; locality: Between Larvik and Kvelde; verbatimElevation: 25; decimalLatitude: 59.1525; decimalLongitude: 10.0244; **Event:** samplingProtocol: T; eventDate: 03/10/2002; habitat: Litter, soil
- **Type status:** Other material. **Occurrence:** recordNumber: 2 DN; recordedBy: MG; **Location:** county: NOR-Vestfold; locality: Rien, in the vicinity of Odberg; verbatimElevation: 50; decimalLatitude: 59.2708; decimalLongitude: 9.9300; **Event:** samplingProtocol: T; eventDate: 03/10/2002; habitat: At the base of tree trunk, soil, humus
- **Type status:** Other material. **Occurrence:** recordNumber: 1 PL; recordedBy: JS; **Location:** county: SWE-Stockholm; locality: Norrtälje, St. Dylanda; verbatimElevation: 35; decimalLatitude: 59.7078; decimalLongitude: 18.2332; **Event:** samplingProtocol: S; eventDate: 18/06/2011; habitat: At edge of forest, near forest stream and arable area
- **Type status:** Other material. **Occurrence:** recordNumber: 1 DN; recordedBy: PTL; **Location:** county: FIN-Southwest Finland; locality: Parainen, Lenholm; decimalLatitude: 60.2; decimalLongitude: 22.1; **Event:** eventDate: 26/02/1995; habitat: Oak forest with junipers and *Hepatica triloba*
- **Type status:** Other material. **Occurrence:** recordNumber: 1 ♀; recordedBy: JM; **Location:** county: FIN-Åland Islands; locality: Åland, Lemland, Bergö; decimalLatitude: 60.0; decimalLongitude: 19.8; **Event:** samplingProtocol: U; eventDate: 17/08/1997; habitat: Litter of rowan tree in the mixed forest

##### Distribution

Norway (Mąkol and Gulvik 2002) and new for Sweden and Finland.

#### Ettmuelleria sucida

(Trägårdh, 1910) [L]

##### Distribution

Sweden (Trägårdh 1910).

##### Notes

*Sucidothrombium sucidum* (L. Koch, 1879) and *Ettmuelleria sucida* (Trägårdh, 1910) represent distinct genera and species (Southcott 1994, Mąkol and Gabryś 2002).

#### Microtrombidium pusillum

(Hermann, 1804) [PL, L]

http://www.gbif.org/species/4540697

##### Materials

- **Type status:** Other material. **Occurrence:** recordNumber: 5 AD; recordedBy: PTL; **Location:** county: FIN-Southwest Finland; locality: Parainen, Sydmo, Ippos; decimalLatitude: 60.2; decimalLongitude: 22.1; **Event:** eventDate: 06/09/1966; habitat: Pine mire
- **Type status:** Other material. **Occurrence:** recordNumber: 2 AD, 1 DN; recordedBy: PTL; **Location:** county: FIN-Southwest Finland; locality: Parainen, Mustfinno; decimalLatitude: 60.2; decimalLongitude: 22.1; **Event:** eventDate: 10/10/1966

##### Distribution

Norway (Thor 1900b) and new for Finland.

#### Microtrombidium spiniferum

(Thor, 1900) [PL]

http://www.gbif.org/species/4540690

##### Distribution

Norway (Thor 1900b).

##### Notes

Only single record in literature (Thor 1900b, as *Ottonia spinifera*) and no recent occurrences since then. Identification questionable.

#### Microtrombidium strandi

(Thor, 1900) [PL]

http://www.gbif.org/species/4540699

##### Distribution

Norway (Thor 1900b).

##### Notes

Only single record in literature (Thor 1900b, as *Ottonia strandi*) and no recent occurrences since then. Identification questionable.

#### Platytrombidium fasciatum

(C.L. Koch, 1836) [PL, L]

http://www.gbif.org/species/4540649

##### Distribution

Norway (Berlese 1910).

##### Notes

Microtrombidium (Enemothrombium) quadrispinum Berlese, 1910 is a junior synonym (Gabryś et al. 2005). Only single record in literature (Berlese 1910) and no recent occurrences since then. Identification questionable.

#### Sucidothrombium sucidum

(L. Koch, 1879) [PL, L]

http://www.gbif.org/species/4540658

##### Materials

- **Type status:** Other material. **Occurrence:** recordNumber: 1 ♀, 1 AD, 5 DN,; recordedBy: MG; **Location:** county: NOR-Buskerud; locality: Bakkestølen; verbatimElevation: 800; decimalLatitude: 60.9000; decimalLongitude: 8.2875; **Event:** samplingProtocol: T; eventDate: 26/08/2000; habitat: Soil, humus, moss, undergrowth
- **Type status:** Other material. **Occurrence:** recordNumber: 1 ♀, 16 AD, 14 DN; recordedBy: MG; **Location:** county: NOR-Hordaland; locality: Trondsbu, Hardangervidda; verbatimElevation: 1 200; decimalLatitude: 60.2906; decimalLongitude: 7.5481; **Event:** samplingProtocol: T; eventDate: 22/08/1997; habitat: Moist soil, *Salix lapponum*
- **Type status:** Other material. **Occurrence:** recordNumber: 1 ♀, 7 DN; recordedBy: MG, AS; **Location:** county: NOR-Hordaland; locality: Vikafjellet; verbatimElevation: 700; decimalLatitude: 60.9106; decimalLongitude: 6.4517; **Event:** samplingProtocol: T; eventDate: 05/07/2004; habitat: Moss by the creek
- **Type status:** Other material. **Occurrence:** recordNumber: 1 DN; recordedBy: MG, AK; **Location:** county: NOR-Oppland; locality: Sognefjellet; verbatimElevation: 1 400; decimalLatitude: 61.5647; decimalLongitude: 7.9947; **Event:** samplingProtocol: T; eventDate: 22/06/2000; habitat: Soil, humus, moss, undergrowth
- **Type status:** Other material. **Occurrence:** recordNumber: 2 ♀, 3 AD; recordedBy: MG, GM; **Location:** county: NOR-Oppland; locality: Sognefjellet; verbatimElevation: 1 400; decimalLatitude: 61.5617; decimalLongitude: 7.9836; **Event:** samplingProtocol: T; eventDate: 10/08/2000; habitat: Soil, moss, undergrowth
- **Type status:** Other material. **Occurrence:** recordNumber: 3 DN; recordedBy: MG, GM; **Location:** county: NOR-Oppland; locality: Sognefjellet, in the vicinity of Krossbu; verbatimElevation: 1 450; decimalLatitude: 61.5667; decimalLongitude: 8.0183; **Event:** samplingProtocol: T; eventDate: 10/08/2000; habitat: Soil, moss, undergrowth
- **Type status:** Other material. **Occurrence:** recordNumber: 4 ♀, 25 AD, 22 DN; recordedBy: MG, GM; **Location:** county: NOR-Oppland; locality: Sognefjellet; verbatimElevation: 1 430; decimalLatitude: 61.5600; decimalLongitude: 8.0014; **Event:** samplingProtocol: T; eventDate: 02/09/2000; habitat: Soil, moss, undergrowth
- **Type status:** Other material. **Occurrence:** recordNumber: 1 AD, 5 L; recordedBy: MG, SS; **Location:** county: NOR-Oppland; locality: Sognefjellet; verbatimElevation: 1 400; decimalLatitude: 61.5600; decimalLongitude: 7.9628; **Event:** samplingProtocol: T; eventDate: 17/05/2003; habitat: Moss, soil
- **Type status:** Other material. **Occurrence:** recordNumber: 2 AD, 3 DN; recordedBy: MG, JŁ; **Location:** county: NOR-Oppland; locality: Sognefjellet; verbatimElevation: 1 300; decimalLatitude: 61.5600; decimalLongitude: 7.9681; **Event:** samplingProtocol: T; eventDate: 20/08/2006; habitat: Soil, moss, undergrowth
- **Type status:** Other material. **Occurrence:** recordNumber: 1 ♀; recordedBy: MG; **Location:** county: NOR-Sogn og Fjordane; locality: Galdane; verbatimElevation: 300; decimalLatitude: 61.0611; decimalLongitude: 7.7278; **Event:** samplingProtocol: T; eventDate: 07/06/2001; habitat: Soil, moss, undergrowth
- **Type status:** Other material. **Occurrence:** recordNumber: 2 AD, 1 DN; recordedBy: MG; **Location:** county: NOR-Sogn og Fjordane; locality: In the vicinity of Nystølen; verbatimElevation: 725; decimalLatitude: 61.3442; decimalLongitude: 6.4664; **Event:** samplingProtocol: T; eventDate: 16/09/2001; habitat: Moist humus
- **Type status:** Other material. **Occurrence:** recordNumber: 1 AD; recordedBy: MG; **Location:** county: NOR-Sogn og Fjordane; locality: Kusslia, in the vicinity of Moskog; verbatimElevation: 50; decimalLatitude: 61.4347; decimalLongitude: 5.9583; **Event:** samplingProtocol: T; eventDate: 16/09/2001; habitat: Moss and vegetation from elm tree trunk
- **Type status:** Other material. **Occurrence:** recordNumber: 2 DN; recordedBy: MG; **Location:** county: NOR-Sogn og Fjordane; locality: Vikafjellet; verbatimElevation: 980; decimalLatitude: 60.9964; decimalLongitude: 6.5461; **Event:** samplingProtocol: T; eventDate: 23/09/2001; habitat: Moss, soil, humus
- **Type status:** Other material. **Occurrence:** recordNumber: 4 DN; recordedBy: MG, PS, SS; **Location:** county: NOR-Sogn og Fjordane; locality: In the vicinity of Nystølen; verbatimElevation: 500; decimalLatitude: 61.3392; decimalLongitude: 6.3536; **Event:** samplingProtocol: T; eventDate: 04/07/2002; habitat: Moss from small peatbog
- **Type status:** Other material. **Occurrence:** recordNumber: 1 ♀, 2 DN; recordedBy: MG, PS, SS; **Location:** county: NOR-Sogn og Fjordane; locality: Nystølen; verbatimElevation: 700; decimalLatitude: 61.3436; decimalLongitude: 6.4564; **Event:** samplingProtocol: T; eventDate: 04/07/2002; habitat: Moss, soil
- **Type status:** Other material. **Occurrence:** recordNumber: 3 ♀, 6 AD, 23 DN; recordedBy: MG, MK; **Location:** county: NOR-Sogn og Fjordane; locality: Sognefjellet, in the vicinity of Turtagrø; verbatimElevation: 1 200; decimalLatitude: 61.5208; decimalLongitude: 7.8258; **Event:** samplingProtocol: T; eventDate: 04/08/2003; habitat: Soil, moss, undergrowth
- **Type status:** Other material. **Occurrence:** recordNumber: 6 PL; recordedBy: PTL; **Location:** county: FIN-Northern Ostrobothnia; locality: Oulunsalo, Varjakka; decimalLatitude: 65.0; decimalLongitude: 25.3; **Event:** eventDate: 13/06/1987; habitat: Seashore meadow

##### Distribution

Norway (Trägårdh 1904, Berlese 1910, Mąkol and Gabryś 2002, Mąkol and Gulvik 2002), Sweden (Trägårdh 1902) and new for Finland.

##### Notes

Microtrombidium pusillum var. pingue Berlese, 1910 and *Microtrombidium norvegicum* Berlese, 1910 are junior synonyms (Berlese 1912, Gabryś 1999).

#### Valgothrombiinae

Gabryś, 1999

#### Enemothrombium bifoliosum

(Canestrini, 1884) [PL, L]

http://www.gbif.org/species/4540602

##### Materials

- **Type status:** Other material. **Occurrence:** recordNumber: 1 DN; recordedBy: MG; **Location:** county: NOR-Hordaland; locality: Hola; verbatimElevation: 570; decimalLatitude: 60.9019; decimalLongitude: 6.4836; **Event:** samplingProtocol: T; eventDate: 25/06/2005; habitat: Litter, moss
- **Type status:** Other material. **Occurrence:** recordNumber: 1 DN; recordedBy: MG; **Location:** county: NOR-Sogn og Fjordane; locality: Anda; verbatimElevation: 100; decimalLatitude: 61.8461; decimalLongitude: 6.0817; **Event:** samplingProtocol: T; eventDate: 02/02/2001; habitat: Litter, soil
- **Type status:** Other material. **Occurrence:** recordNumber: 1 ♀, 1 DN; recordedBy: MG, PS, SS; **Location:** county: NOR-Sogn og Fjordane; locality: In the vicinity of Nystølen; verbatimElevation: 500; decimalLatitude: 61.3392; decimalLongitude: 6.3536; **Event:** samplingProtocol: T; eventDate: 04/07/2002; habitat: Moss from small peatbog
- **Type status:** Other material. **Occurrence:** recordNumber: 3 PL; recordedBy: PTL; **Location:** county: FIN-Southwest Finland; locality: Parainen, Sydmo, Ippos; decimalLatitude: 60.2; decimalLongitude: 22.1; **Event:** eventDate: 06/09/1966; habitat: Pine mire
- **Type status:** Other material. **Occurrence:** recordNumber: 1 PL; recordedBy: PTL; **Location:** county: FIN-Southwest Finland; locality: Nauvo, Sandö; decimalLatitude: 60.17; decimalLongitude: 22.09; **Event:** eventDate: 09/09/1992; habitat: Pondside mire with *Calla*, *Comarum*, *Carex* spp.

##### Distribution

Norway (Mąkol and Gulvik 2002) and new for Finland.

#### Valgothrombium alpinum

Willmann, 1940 [PL]

http://www.gbif.org/species/4540740

##### Materials

- **Type status:** Other material. **Occurrence:** recordNumber: 1 AD; recordedBy: MG; **Location:** county: NOR-Sogn og Fjordane; locality: Hafslo; verbatimElevation: 325; decimalLatitude: 61.3264; decimalLongitude: 7.2175; **Event:** samplingProtocol: T; eventDate: 08/08/2003; habitat: Soil, undergrowth
- **Type status:** Other material. **Occurrence:** recordNumber: 2 DN; recordedBy: MG; **Location:** county: NOR-Vestfold; locality: Larvik; verbatimElevation: 100; decimalLatitude: 59.0703; decimalLongitude: 10.0341; **Event:** samplingProtocol: T; eventDate: 03/10/2002; habitat: Moss, soil

##### Distribution

New for Norway.

#### Valgothrombium confusum

(Berlese, 1910) [PL]

http://www.gbif.org/species/4540741

##### Materials

- **Type status:** Other material. **Occurrence:** recordNumber: 1 AD; recordedBy: MG, GM; **Location:** county: NOR-Sogn og Fjordane; locality: Balestrand; verbatimElevation: 100; decimalLatitude: 61.2081; decimalLongitude: 6.5281; **Event:** samplingProtocol: T; eventDate: 31/07/2001; habitat: Rot from decaying tree trunk
- **Type status:** Other material. **Occurrence:** recordNumber: 1 DN; recordedBy: MG; **Location:** county: NOR-Vestfold; locality: In the vicinity of Berg; verbatimElevation: 50; decimalLatitude: 58.9886; decimalLongitude: 9.9003; **Event:** samplingProtocol: T; eventDate: 03/10/2002; habitat: Rot from decaying tree trunk
- **Type status:** Other material. **Occurrence:** recordNumber: 1 ♀, 1 AD; recordedBy: SK; **Location:** county: FIN-Southwest Finland; locality: Kaarina, Kuusisto; decimalLatitude: 60.3; decimalLongitude: 22.3; **Event:** samplingProtocol: P; eventDate: 15/07/1989 - 23/09/1989; habitat: Oak forest

##### Distribution

New for Norway and Finland.

#### Valgothrombium major

(Halbert, 1920) [PL]

http://www.gbif.org/species/4540734

##### Materials

- **Type status:** Other material. **Occurrence:** recordNumber: 2 AD; recordedBy: MG, PS, SS; **Location:** county: NOR-Sogn og Fjordane; locality: In the vicinity of Nystølen; verbatimElevation: 500; decimalLatitude: 61.3392; decimalLongitude: 6.3536; **Event:** samplingProtocol: T; eventDate: 04/07/2002; habitat: Moss from small peatbog

##### Distribution

Sweden (Willmann 1943) and new for Norway.

#### Valgothrombium mariae

Mąkol & Łaydanowicz, 2010 [L]

http://www.gbif.org/species/7426675

##### Materials

- **Type status:** Other material. **Occurrence:** recordNumber: 2 L; recordedBy: MG; **Location:** county: NOR-Sogn og Fjordane; locality: In the vicinity of Skjolden; verbatimElevation: 50; decimalLatitude: 61.4903; decimalLongitude: 7.5736; **Event:** samplingProtocol: T; eventDate: 08/05/2002; habitat: Rot from decaying tree trunk

##### Distribution

Norway (Mąkol and Łaydanowicz 2010).

#### Valgothrombium stuarti

Baker, 1999 [L]

http://www.gbif.org/species/4540736

##### Materials

- **Type status:** Other material. **Occurrence:** recordNumber: 1 L; recordedBy: SMTP; **Location:** county: SWE-Uppsala; locality: Biskops-Arnö (=TrapID 8); verbatimElevation: 10; decimalLatitude: 59.6721; decimalLongitude: 17.5009; **Event:** samplingProtocol: M; eventDate: 20/06/2005 - 18/07/2005 (=Coll.ID 1602); habitat: Northern beach, elm grove

##### Distribution

New for Sweden.

#### Valgothrombium valgum

(George, 1909) [PL, L]

http://www.gbif.org/species/4540748

##### Materials

- **Type status:** Other material. **Occurrence:** recordNumber: 1 AD, 3 DN; recordedBy: PTL; **Location:** county: FIN-Southwest Finland; locality: Parainen, Lapplahti, Kalastajakopinranta; decimalLatitude: 60.29; decimalLongitude: 22.09; **Event:** eventDate: 26/07/1982; habitat: Limestone with *Thymus*, *Sedum annum* etc.
- **Type status:** Other material. **Occurrence:** recordNumber: 1 ♀, 1 ♂, 1 AD, 1 DN; recordedBy: PTL; **Location:** county: FIN-Southwest Finland; locality: Nauvo, Sandö; decimalLatitude: 60.17; decimalLongitude: 22.09; **Event:** eventDate: 9/09/1992; habitat: Pondside mire with *Calla*, *Comarum*, *Carex* spp.

##### Distribution

New for Finland.

#### Podothrombiidae

Thor, 1935

#### Podothrombium bicolor

(Hermann, 1804) [PL]

http://www.gbif.org/species/4541020

##### Materials

- **Type status:** Other material. **Occurrence:** recordNumber: 1 DN; recordedBy: MG; **Location:** county: NOR-Buskerud; locality: Granheim, by the river Hemsil; verbatimElevation: 550; decimalLatitude: 60.7783; decimalLongitude: 8.7597; **Event:** samplingProtocol: T; eventDate: 26/08/2000; habitat: By the river, soil, moss, litter, undergrowth
- **Type status:** Other material. **Occurrence:** recordNumber: 1 DN; recordedBy: MG; **Location:** county: NOR-Buskerud; locality: Komnes; verbatimElevation: 80; decimalLatitude: 59.5078; decimalLongitude: 9.9167; **Event:** samplingProtocol: T; eventDate: 03/10/2002; habitat: Moss, litter
- **Type status:** Other material. **Occurrence:** recordNumber: 1 AD, 9 DN; recordedBy: MG; **Location:** county: NOR-Buskerud; locality: Efteløt; verbatimElevation: 70; decimalLatitude: 59.5517; decimalLongitude: 9.8042; **Event:** samplingProtocol: T; eventDate: 03/10/2002; habitat: Moss, soil, litter
- **Type status:** Other material. **Occurrence:** recordNumber: 1 DN; recordedBy: MG; **Location:** county: NOR-Buskerud; locality: Near Hostvet, at the level of Lande; verbatimElevation: 80; decimalLatitude: 59.6083; decimalLongitude: 9.7425; **Event:** samplingProtocol: T; eventDate: 31/09/2002; habitat: Soil, moss, litter
- **Type status:** Other material. **Occurrence:** recordNumber: 1 AD; recordedBy: MG; **Location:** county: NOR-Hordaland; locality: Skreidi, between Vaksdal and Fossmarki; verbatimElevation: 50; decimalLatitude: 60.4939; decimalLongitude: 5.7339; **Event:** samplingProtocol: T; eventDate: 06/07/2001; habitat: Moss, soil, humus
- **Type status:** Other material. **Occurrence:** recordNumber: 3 DN; recordedBy: MG; **Location:** county: NOR-Sogn og Fjordane; locality: Mo, in the vicinity of Moskog; verbatimElevation: 100; decimalLatitude: 61.4364; decimalLongitude: 5.9856; **Event:** samplingProtocol: T; eventDate: 11/10/2000; habitat: Moss, litter, soil, rot
- **Type status:** Other material. **Occurrence:** recordNumber: 1 DN; recordedBy: MG, JM; **Location:** county: NOR-Sogn og Fjordane; locality: Hafslo; verbatimElevation: 300; decimalLatitude: 61.3314; decimalLongitude: 7.2144; **Event:** samplingProtocol: T; eventDate: 25/05/2001; habitat: Soil, humus, undergrowth
- **Type status:** Other material. **Occurrence:** recordNumber: 1 DN; recordedBy: MG; **Location:** county: NOR-Sogn og Fjordane; locality: Vikafjellet; verbatimElevation: 950; decimalLatitude: 60.9917; decimalLongitude: 6.5403; **Event:** samplingProtocol: T; eventDate: 23/09/2001; habitat: Moss, soil, undergrowth
- **Type status:** Other material. **Occurrence:** recordNumber: 6 DN; recordedBy: MG; **Location:** county: NOR-Sogn og Fjordane; locality: Hella; verbatimElevation: 100; decimalLatitude: 61.2083; decimalLongitude: 6.5975; **Event:** samplingProtocol: T; eventDate: 23/09/2001; habitat: Moss, soil, litter
- **Type status:** Other material. **Occurrence:** recordNumber: 1 AD; recordedBy: MG, PS, SS; **Location:** county: NOR-Sogn og Fjordane; locality: Hov, in the vicinity of Vik; verbatimElevation: 400; decimalLatitude: 61.3231; decimalLongitude: 6.2603; **Event:** samplingProtocol: T; eventDate: 04/07/2002; habitat: Litter, moss, humus
- **Type status:** Other material. **Occurrence:** recordNumber: 5 DN; recordedBy: MG; **Location:** county: NOR-Sogn og Fjordane; locality: Hafslo; verbatimElevation: 325; decimalLatitude: 61.3264; decimalLongitude: 7.2175; **Event:** samplingProtocol: T; eventDate: 15/09/2002; habitat: Soil, undergrowth
- **Type status:** Other material. **Occurrence:** recordNumber: 1 AD; recordedBy: MG; **Location:** county: NOR-Sogn og Fjordane; locality: In the vicinity of Kroken; verbatimElevation: 50; decimalLatitude: 61.3553; decimalLongitude: 7.3942; **Event:** samplingProtocol: T; eventDate: 20/07/2003; habitat: Soil
- **Type status:** Other material. **Occurrence:** recordNumber: 2 DN; recordedBy: MG; **Location:** county: NOR-Sogn og Fjordane; locality: Between Skjolden and Fortun; verbatimElevation: 25; decimalLatitude: 61.4878; decimalLongitude: 7.6594; **Event:** samplingProtocol: T; eventDate: 25/11/2005; habitat: Moss, decaying bark and humus from tree trunk
- **Type status:** Other material. **Occurrence:** recordNumber: 1 ♀; recordedBy: MG, JŁ; **Location:** county: NOR-Sogn og Fjordane; locality: Between Skjolden and Luster; verbatimElevation: 25; decimalLatitude: 61.4642; decimalLongitude: 7.5303; **Event:** samplingProtocol: T; eventDate: 06/08/2006; habitat: Moss on stones close to stream
- **Type status:** Other material. **Occurrence:** recordNumber: 2 DN; recordedBy: MG, JŁ; **Location:** county: NOR-Sogn og Fjordane; locality: In the vicinity of Skjolden; verbatimElevation: 25; decimalLatitude: 61.4950; decimalLongitude: 7.5867; **Event:** samplingProtocol: T; eventDate: 20/08/2006; habitat: Litter and soil
- **Type status:** Other material. **Occurrence:** recordNumber: 2 DN; recordedBy: MG; **Location:** county: NOR-Sogn og Fjordane; locality: Skjolden; verbatimElevation: 25; decimalLatitude: 61.4914; decimalLongitude: 7.6050; **Event:** samplingProtocol: T; eventDate: 06/09/2006; habitat: Moss, soil, humus, litter at decaying trunk
- **Type status:** Other material. **Occurrence:** recordNumber: 1 DN; recordedBy: MG; **Location:** county: NOR-Sogn og Fjordane; locality: Kvål, in the vicinity of Grimeland; verbatimElevation: 150; decimalLatitude: 61.5122; decimalLongitude: 5.9925; **Event:** samplingProtocol: T; eventDate: 22/09/2006; habitat: Riverside with alder, decomposed wood from tree trunk
- **Type status:** Other material. **Occurrence:** recordNumber: 3 DN; recordedBy: MG; **Location:** county: NOR-Sogn og Fjordane; locality: Leirdal, in the vicinity of Gaupne; verbatimElevation: 75; decimalLatitude: 61.4628; decimalLongitude: 7.2497; **Event:** samplingProtocol: T; eventDate: 25/12/2006; habitat: Decomposed wood and bark
- **Type status:** Other material. **Occurrence:** recordNumber: 1 AD, 10 DN; recordedBy: MG; **Location:** county: NOR-Vestfold; locality: In the vicinity of Berg; verbatimElevation: 50; decimalLatitude: 58.9886; decimalLongitude: 9.9003; **Event:** samplingProtocol: T; eventDate: 03/10/2002; habitat: Soil, moss, litter, undergrowth
- **Type status:** Other material. **Occurrence:** recordNumber: 10 DN; recordedBy: MG; **Location:** county: NOR-Vestfold; locality: Larvik, Bøkeskogen; verbatimElevation: 50; decimalLatitude: 59.0592; decimalLongitude: 10.0200; **Event:** samplingProtocol: T; eventDate: 03/10/2002; habitat: Litter, soil
- **Type status:** Other material. **Occurrence:** recordNumber: 2 DN; recordedBy: MG; **Location:** county: NOR-Vestfold; locality: Rien, in the vicinity of Odberg; verbatimElevation: 50; decimalLatitude: 59.2708; decimalLongitude: 9.9300; **Event:** samplingProtocol: T; eventDate: 03/10/2002; habitat: Litter, soil

##### Distribution

Norway (Thor 1930, Mąkol and Gulvik 2002) and Finland (Nordberg 1936, Mąkol et al. 2006).

#### Podothrombium curtipalpe

(Thor, 1900) [PL]

http://www.gbif.org/species/4541023

##### Distribution

Norway (Thor 1900b), Sweden (Trägårdh 1904, Trägårdh 1910) and Finland (Mąkol et al. 2006).

##### Notes

Species listed by Trägårdh (1904) and Trägårdh (1910) as Trombidium bicolor var. curtipalpe.

#### Podothrombium filipes

(C.L. Koch, 1837) [PL, L]

http://www.gbif.org/species/4541012

##### Materials

- **Type status:** Other material. **Occurrence:** recordNumber: 1 ♀; recordedBy: MG; **Location:** county: NOR-Buskerud; locality: Søre, by the river Hallingdalselve; verbatimElevation: 180; decimalLatitude: 60.4667; decimalLongitude: 9.2456; **Event:** samplingProtocol: T; eventDate: 26/08/2000; habitat: By the river, litter at the base of birch trunk
- **Type status:** Other material. **Occurrence:** recordNumber: 1 DN; recordedBy: MG; **Location:** county: NOR-Buskerud; locality: Noresund; verbatimElevation: 150; decimalLatitude: 60.1800; decimalLongitude: 9.6211; **Event:** samplingProtocol: T; eventDate: 06/06/2001; habitat: Litter
- **Type status:** Other material. **Occurrence:** recordNumber: 3 DN; recordedBy: MG; **Location:** county: NOR-Buskerud; locality: Near Hostvet, at the level of Lande; verbatimElevation: 80; decimalLatitude: 59.6083; decimalLongitude: 9.7425; **Event:** samplingProtocol: T; eventDate: 30/09/2002; habitat: Soil, moss, litter
- **Type status:** Other material. **Occurrence:** recordNumber: 1 DN; recordedBy: MG; **Location:** county: NOR-Hordaland; locality: Trondsbu, Hardangervidda; verbatimElevation: 1 200; decimalLatitude: 60.2906; decimalLongitude: 7.5481; **Event:** samplingProtocol: T; eventDate: 22/08/1997; habitat: Moist soil
- **Type status:** Other material. **Occurrence:** recordNumber: 7 L; recordedBy: MG; **Location:** county: NOR-Hordaland; locality: In the vicinity of Kolstallen and Bergen; verbatimElevation: 50; decimalLatitude: 60.4411; decimalLongitude: 5.4511; **Event:** samplingProtocol: T; eventDate: 04/07/2001; habitat: Moss, soil, litter
- **Type status:** Other material. **Occurrence:** recordNumber: 6 L; recordedBy: MG; **Location:** county: NOR-Hordaland; locality: Blinde; verbatimElevation: 100; decimalLatitude: 60.4639; decimalLongitude: 5.3786; **Event:** samplingProtocol: T; eventDate: 04/07/2001; habitat: Moss, litter, soil, undergrowth
- **Type status:** Other material. **Occurrence:** recordNumber: 4 L; recordedBy: MG; **Location:** county: NOR-Hordaland; locality: Vaksdal; verbatimElevation: 75; decimalLatitude: 60.4708; decimalLongitude: 5.7458; **Event:** samplingProtocol: T; eventDate: 06/07/2001; habitat: Litter and soil from the bed of ravine
- **Type status:** Other material. **Occurrence:** recordNumber: 2 ♀; recordedBy: MG; **Location:** county: NOR-Hordaland; locality: Stalheimskleiva; verbatimElevation: 125; decimalLatitude: 60.8431; decimalLongitude: 6.7111; **Event:** samplingProtocol: T; eventDate: 04/10/2001; habitat: Litter, soil, moss and bark from decaying trunks
- **Type status:** Other material. **Occurrence:** recordNumber: 8 L; recordedBy: MG; **Location:** county: NOR-Hordaland; locality: Haukeland, in the vicinity of Bergen; verbatimElevation: 100; decimalLatitude: 60.3528; decimalLongitude: 5.4478; **Event:** samplingProtocol: T; eventDate: 05/07/2004; habitat: Moist litter
- **Type status:** Other material. **Occurrence:** recordNumber: 18 L; recordedBy: MG; **Location:** county: NOR-Hordaland; locality: Sandviki; verbatimElevation: 50; decimalLatitude: 60.4567; decimalLongitude: 5.6786; **Event:** samplingProtocol: T; eventDate: 05/07/2004; habitat: Moss, humus
- **Type status:** Other material. **Occurrence:** recordNumber: 4 L; recordedBy: MG; **Location:** county: NOR-Hordaland; locality: Between Fossmarki and Stanghelle; verbatimElevation: 50; decimalLatitude: 60.5308; decimalLongitude: 5.7247; **Event:** samplingProtocol: T; eventDate: 05/07/2004; habitat: Litter, humus, moss, lady fern, bird cherry
- **Type status:** Other material. **Occurrence:** recordNumber: 7 L; recordedBy: MG, AS; **Location:** county: NOR-Hordaland; locality: Evanger; verbatimElevation: 50; decimalLatitude: 60.6472; decimalLongitude: 6.1356; **Event:** samplingProtocol: T; eventDate: 05/07/2004; habitat: Overgrown arable area, grassy forest bed, humus, litter, moss
- **Type status:** Other material. **Occurrence:** recordNumber: 5 L; recordedBy: MG; **Location:** county: NOR-Hordaland; locality: In the vicinity of Bulken; verbatimElevation: 75; decimalLatitude: 60.6314; decimalLongitude: 6.2744; **Event:** samplingProtocol: T; eventDate: 25/06/2005; habitat: Rot from decaying tree trunk
- **Type status:** Other material. **Occurrence:** recordNumber: 4 DN; recordedBy: MG; **Location:** county: NOR-Hordaland; locality: In the vicinity of Bulken; verbatimElevation: 25; decimalLatitude: 60.6314; decimalLongitude: 6.2744; **Event:** samplingProtocol: T; eventDate: 25/11/2005; habitat: Moss
- **Type status:** Other material. **Occurrence:** recordNumber: 1 DN; recordedBy: MG; **Location:** county: NOR-Sogn og Fjordane; locality: Nigardsbreen; verbatimElevation: 350; decimalLatitude: 61.6764; decimalLongitude: 7.2083; **Event:** samplingProtocol: T; eventDate: 20/09/2000; habitat: Foots of the Nigardsbreen glacier, near the lake Nigardsbreevatnet, birch forest, rot, very moist
- **Type status:** Other material. **Occurrence:** recordNumber: 2 ♀, 3 DN; recordedBy: MG; **Location:** county: NOR-Sogn og Fjordane; locality: Mo, in the vicinity of Moskog; verbatimElevation: 100; decimalLatitude: 61.4364; decimalLongitude: 5.9856; **Event:** samplingProtocol: T; eventDate: 11/10/2000; habitat: Moss, soil, undergrowth
- **Type status:** Other material. **Occurrence:** recordNumber: 1 DN; recordedBy: MG; **Location:** county: NOR-Sogn og Fjordane; locality: Førde; verbatimElevation: 100; decimalLatitude: 61.4550; decimalLongitude: 5.8581; **Event:** samplingProtocol: T; eventDate: 11/10/2000; habitat: Soil, moss
- **Type status:** Other material. **Occurrence:** recordNumber: 1 DN; recordedBy: MG; **Location:** county: NOR-Sogn og Fjordane; locality: In the vicinity of Bøyabreen; verbatimElevation: 150; decimalLatitude: 61.4811; decimalLongitude: 6.7419; **Event:** samplingProtocol: T; eventDate: 11/10/2000; habitat: Litter
- **Type status:** Other material. **Occurrence:** recordNumber: 1 DN; recordedBy: MG; **Location:** county: NOR-Sogn og Fjordane; locality: Near Kjøsnesfjorden, in the vicinity of Kjøsnes; verbatimElevation: 250; decimalLatitude: 61.5419; decimalLongitude: 6.5378; **Event:** samplingProtocol: T; eventDate: 11/10/2000; habitat: Soil, undergrowth
- **Type status:** Other material. **Occurrence:** recordNumber: 2 DN; recordedBy: MG; **Location:** county: NOR-Sogn og Fjordane; locality: Near Kjøsnesfjorden, in the vicinity of Kjøsnes; verbatimElevation: 250; decimalLatitude: 61.5447; decimalLongitude: 6.5106; **Event:** samplingProtocol: T; eventDate: 11/10/2000; habitat: Litter
- **Type status:** Other material. **Occurrence:** recordNumber: 2 DN; recordedBy: MG; **Location:** county: NOR-Sogn og Fjordane; locality: Grinde; verbatimElevation: 120; decimalLatitude: 61.1847; decimalLongitude: 6.7406; **Event:** samplingProtocol: T; eventDate: 13/11/2000; habitat: Rot from tree trunk
- **Type status:** Other material. **Occurrence:** recordNumber: 1 DN; recordedBy: MG; **Location:** county: NOR-Sogn og Fjordane; locality: Anda; verbatimElevation: 100; decimalLatitude: 61.8461; decimalLongitude: 6.0817; **Event:** samplingProtocol: T; eventDate: 02/02/2001; habitat: Litter, soil
- **Type status:** Other material. **Occurrence:** recordNumber: 10 L; recordedBy: MG, GM; **Location:** county: NOR-Sogn og Fjordane; locality: Balestrand; verbatimElevation: 100; decimalLatitude: 61.2081; decimalLongitude: 6.5281; **Event:** samplingProtocol: T; eventDate: 31/07/2001; habitat: Soil, moss, undergrowth
- **Type status:** Other material. **Occurrence:** recordNumber: 6 L; recordedBy: MG, GM; **Location:** county: NOR-Sogn og Fjordane; locality: Ytredalen, in the vicinity of Vadheim; verbatimElevation: 100; decimalLatitude: 61.2336; decimalLongitude: 5.8225; **Event:** samplingProtocol: T; eventDate: 31/07/2001; habitat: Soil, moss, undergrowth
- **Type status:** Other material. **Occurrence:** recordNumber: 1 L; recordedBy: MG, GM; **Location:** county: NOR-Sogn og Fjordane; locality: Sande; verbatimElevation: 75; decimalLatitude: 61.3247; decimalLongitude: 5.7947; **Event:** samplingProtocol: T; eventDate: 31/07/2001; habitat: Moss, soil
- **Type status:** Other material. **Occurrence:** recordNumber: 2 L; recordedBy: MG, GM; **Location:** county: NOR-Sogn og Fjordane; locality: In the vicinity of Førde; verbatimElevation: 300; decimalLatitude: 61.4108; decimalLongitude: 5.8178; **Event:** samplingProtocol: T; eventDate: 31/07/2001; habitat: Moss, soil, undergrowth
- **Type status:** Other material. **Occurrence:** recordNumber: 1 ♀, 1 L; recordedBy: MG, GM; **Location:** county: NOR-Sogn og Fjordane; locality: In the vicinity of Urnes; verbatimElevation: 50; decimalLatitude: 61.3072; decimalLongitude: 7.3422; **Event:** samplingProtocol: T; eventDate: 19/08/2001; habitat: Moss, soil
- **Type status:** Other material. **Occurrence:** recordNumber: 1 DN; recordedBy: MG; **Location:** county: NOR-Sogn og Fjordane; locality: In the vicinity of Nystølen; verbatimElevation: 725; decimalLatitude: 61.3442; decimalLongitude: 6.4664; **Event:** samplingProtocol: T; eventDate: 16/09/2001; habitat: Soil, moss, undergrowth
- **Type status:** Other material. **Occurrence:** recordNumber: 1 ♀, 7 DN; recordedBy: MG; **Location:** county: NOR-Sogn og Fjordane; locality: Kusslia, in the vicinity of Moskog; verbatimElevation: 50; decimalLatitude: 61.4347; decimalLongitude: 5.9583; **Event:** samplingProtocol: T; eventDate: 16/09/2001; habitat: Soil, undergrowth
- **Type status:** Other material. **Occurrence:** recordNumber: 13 DN, 3 L; recordedBy: MG; **Location:** county: NOR-Sogn og Fjordane; locality: Astruptunet; verbatimElevation: 200; decimalLatitude: 61.5119; decimalLongitude: 6.3028; **Event:** samplingProtocol: T; eventDate: 16/09/2001; habitat: Moss, soil, undergrowth
- **Type status:** Other material. **Occurrence:** recordNumber: 1 DN; recordedBy: MG; **Location:** county: NOR-Sogn og Fjordane; locality: Vikafjellet; verbatimElevation: 950; decimalLatitude: 60.9917; decimalLongitude: 6.5403; **Event:** samplingProtocol: T; eventDate: 23/09/2001; habitat: Moss, soil, undergrowth
- **Type status:** Other material. **Occurrence:** recordNumber: 1 DN; recordedBy: MG; **Location:** county: NOR-Sogn og Fjordane; locality: Grinde; verbatimElevation: 120; decimalLatitude: 61.1847; decimalLongitude: 6.7406; **Event:** samplingProtocol: T; eventDate: 19/10/2001; habitat: Rot from elm tree trunk
- **Type status:** Other material. **Occurrence:** recordNumber: 1 ♀; recordedBy: MG; **Location:** county: NOR-Sogn og Fjordane; locality: Svanøy; verbatimElevation: 100; decimalLatitude: 61.4828; decimalLongitude: 5.0972; **Event:** samplingProtocol: T; eventDate: 11/11/2001; habitat: Moss, soil, undergrowth
- **Type status:** Other material. **Occurrence:** recordNumber: 2 L; recordedBy: MG, PS, SS; **Location:** county: NOR-Sogn og Fjordane; locality: In the vicinity of Nystølen; verbatimElevation: 500; decimalLatitude: 61.3392; decimalLongitude: 6.3536; **Event:** samplingProtocol: T; eventDate: 04/07/2002; habitat: Bark with moss from the edge of forest covering the upper part of moraine
- **Type status:** Other material. **Occurrence:** recordNumber: 1 AD, 14 DN; recordedBy: MG; **Location:** county: NOR-Sogn og Fjordane; locality: Hafslo; verbatimElevation: 325; decimalLatitude: 61.3264; decimalLongitude: 7.2175; **Event:** samplingProtocol: T; eventDate: 15/09/2002; habitat: Soil, undergrowth
- **Type status:** Other material. **Occurrence:** recordNumber: 4 DN; recordedBy: MG, GM; **Location:** county: NOR-Sogn og Fjordane; locality: Mørkri, in the vicinity of Skjolden; verbatimElevation: 150; decimalLatitude: 61.5567; decimalLongitude: 7.6208; **Event:** samplingProtocol: T; eventDate: 20/04/2003; habitat: Moss from decaying elm branch, soil, undergrowth
- **Type status:** Other material. **Occurrence:** recordNumber: 4 L; recordedBy: MG; **Location:** county: NOR-Sogn og Fjordane; locality: Urnes; verbatimElevation: 50; decimalLatitude: 61.3017; decimalLongitude: 7.3203; **Event:** samplingProtocol: T; eventDate: 20/07/2003; habitat: Moss, soil, undergrowth
- **Type status:** Other material. **Occurrence:** recordNumber: 9 L; recordedBy: MG; **Location:** county: NOR-Sogn og Fjordane; locality: Feigum; verbatimElevation: 25; decimalLatitude: 61.3839; decimalLongitude: 7.4239; **Event:** samplingProtocol: T; eventDate: 20/07/2003; habitat: Moss by the stream mouth
- **Type status:** Other material. **Occurrence:** recordNumber: 1 ♂; recordedBy: MG; **Location:** county: NOR-Sogn og Fjordane; locality: Hafslo; verbatimElevation: 325; decimalLatitude: 61.3264; decimalLongitude: 7.2175; **Event:** samplingProtocol: T; eventDate: 08/08/2003; habitat: Soil, undergrowth
- **Type status:** Other material. **Occurrence:** recordNumber: 1 DN; recordedBy: MG; **Location:** county: NOR-Sogn og Fjordane; locality: Hafslo; verbatimElevation: 325; decimalLatitude: 61.3264; decimalLongitude: 7.2175; **Event:** samplingProtocol: T; eventDate: 20/09/2003; habitat: Soil, undergrowth
- **Type status:** Other material. **Occurrence:** recordNumber: 1 DN; recordedBy: MG; **Location:** county: NOR-Sogn og Fjordane; locality: Hafslo; verbatimElevation: 325; decimalLatitude: 61.3264; decimalLongitude: 7.2175; **Event:** samplingProtocol: T; eventDate: 28/09/2003; habitat: Soil, undergrowth
- **Type status:** Other material. **Occurrence:** recordNumber: 1 AD; recordedBy: MG; **Location:** county: NOR-Sogn og Fjordane; locality: In the vicinity of Feigum; verbatimElevation: 25; decimalLatitude: 61.4006; decimalLongitude: 7.4697; **Event:** samplingProtocol: T; eventDate: 20/07/2004; habitat: Humus
- **Type status:** Other material. **Occurrence:** recordNumber: 2 DN; recordedBy: MG; **Location:** county: NOR-Sogn og Fjordane; locality: In the vicinity of Skjolden; verbatimElevation: 25; decimalLatitude: 61.4950; decimalLongitude: 7.5867; **Event:** samplingProtocol: T; eventDate: 23/03/2005; habitat: Humus, bark at the base of *Salix caprea*
- **Type status:** Other material. **Occurrence:** recordNumber: 1 DN; recordedBy: MG, GM; **Location:** county: NOR-Sogn og Fjordane; locality: Jostedalen, in the vicinity of Gaupne; verbatimElevation: 50; decimalLatitude: 61.4733; decimalLongitude: 7.2547; **Event:** samplingProtocol: T; eventDate: 15/05/2005; habitat: Soil, humus, at the base of alder trees
- **Type status:** Other material. **Occurrence:** recordNumber: 3 DN; recordedBy: MG, GM; **Location:** county: NOR-Sogn og Fjordane; locality: Jostedalen, in the vicinity of Gaupne; verbatimElevation: 50; decimalLatitude: 61.4917; decimalLongitude: 7.2681; **Event:** samplingProtocol: T; eventDate: 15/05/2005; habitat: Rot from alder tree trunk, humus
- **Type status:** Other material. **Occurrence:** recordNumber: 3 ♀, 1 AD, 8 DN; recordedBy: MG; **Location:** county: NOR-Sogn og Fjordane; locality: Mørkri, in the vicinity of Skjolden; verbatimElevation: 150; decimalLatitude: 61.5567; decimalLongitude: 7.6208; **Event:** samplingProtocol: T; eventDate: 30/08/2005; habitat: Meadow with trees, moss, litter, soil, rot
- **Type status:** Other material. **Occurrence:** recordNumber: 2 DN; recordedBy: MG; **Location:** county: NOR-Sogn og Fjordane; locality: Between Skjolden and Luster; verbatimElevation: 25; decimalLatitude: 61.4639; decimalLongitude: 7.5300; **Event:** samplingProtocol: T; eventDate: 05/11/2005; habitat: Moss, leaves, branches, soil
- **Type status:** Other material. **Occurrence:** recordNumber: 4 DN; recordedBy: MG; **Location:** county: NOR-Sogn og Fjordane; locality: Between Skjolden and Luster; verbatimElevation: 25; decimalLatitude: 61.4719; decimalLongitude: 7.5461; **Event:** samplingProtocol: T; eventDate: 05/11/2005; habitat: Forest edge, sandy-clayey soil with moss
- **Type status:** Other material. **Occurrence:** recordNumber: 1 DN; recordedBy: MG; **Location:** county: NOR-Sogn og Fjordane; locality: Between Skjolden and Fortun; verbatimElevation: 25; decimalLatitude: 61.4878; decimalLongitude: 7.6594; **Event:** samplingProtocol: T; eventDate: 25/11/2005; habitat: Moss from birch tree trunk
- **Type status:** Other material. **Occurrence:** recordNumber: 1 L; recordedBy: JŁ, MG; **Location:** county: NOR-Sogn og Fjordane; locality: In the vicinity of Opptun; verbatimElevation: 600; decimalLatitude: 61.4978; decimalLongitude: 7.7492; **Event:** samplingProtocol: C; eventDate: 01/07/2006; habitat: Bushes
- **Type status:** Other material. **Occurrence:** recordNumber: 7 L; recordedBy: JŁ, MG; **Location:** county: NOR-Sogn og Fjordane; locality: Opptun; verbatimElevation: 400; decimalLatitude: 61.4997; decimalLongitude: 7.7247; **Event:** samplingProtocol: T; eventDate: 07/08/2006; habitat: Humus and moss by the riverside
- **Type status:** Other material. **Occurrence:** recordNumber: 1 DN; recordedBy: MG; **Location:** county: NOR-Sogn og Fjordane; locality: Skjolden; verbatimElevation: 25; decimalLatitude: 61.4914; decimalLongitude: 7.6050; **Event:** samplingProtocol: T; eventDate: 06/09/2006; habitat: Moss and soil
- **Type status:** Other material. **Occurrence:** recordNumber: 1 ♀, 1 DN; recordedBy: MG; **Location:** county: NOR-Sogn og Fjordane; locality: Skjolden; verbatimElevation: 25; decimalLatitude: 61.4914; decimalLongitude: 7.6050; **Event:** samplingProtocol: T; eventDate: 06/09/2006; habitat: Moss, soil, humus
- **Type status:** Other material. **Occurrence:** recordNumber: 1 AD, 15 DN; recordedBy: MG; **Location:** county: NOR-Sogn og Fjordane; locality: Kvål, in the vicinity of Grimeland; verbatimElevation: 150; decimalLatitude: 61.5122; decimalLongitude: 5.9925; **Event:** samplingProtocol: T; eventDate: 22/09/2006; habitat: Moss, soil, humus, rot by the riverside, alder trees
- **Type status:** Other material. **Occurrence:** recordNumber: 1 ♀, 5 DN; recordedBy: MG; **Location:** county: NOR-Sogn og Fjordane; locality: In the vicinity of Skei; verbatimElevation: 200; decimalLatitude: 61.6483; decimalLongitude: 6.5200; **Event:** samplingProtocol: T; eventDate: 22/09/2006; habitat: Soil, moss and rot from surroundings of grey alder
- **Type status:** Other material. **Occurrence:** recordNumber: 1 DN; recordedBy: MG, MW; **Location:** county: NOR-Sogn og Fjordane; locality: In the vicinity of Skjolden; verbatimElevation: 75; decimalLatitude: 61.4994; decimalLongitude: 7.6097; **Event:** samplingProtocol: T; eventDate: 09/12/2006; habitat: Litter and soil at the base of birch
- **Type status:** Other material. **Occurrence:** recordNumber: 1 DN; recordedBy: MG, MW; **Location:** county: NOR-Sogn og Fjordane; locality: In the vicinity of Skjolden; verbatimElevation: 75; decimalLatitude: 61.4994; decimalLongitude: 7.6097; **Event:** samplingProtocol: T; eventDate: 09/12/2006; habitat: House garden, decaying raspberry shrubs
- **Type status:** Other material. **Occurrence:** recordNumber: 7 DN; recordedBy: MG; **Location:** county: NOR-Sogn og Fjordane; locality: Leirdal, in the vicinity of Gaupne; verbatimElevation: 75; decimalLatitude: 61.4628; decimalLongitude: 7.2497; **Event:** samplingProtocol: T; eventDate: 25/12/2006; habitat: Decomposed wood, humus, moss
- **Type status:** Other material. **Occurrence:** recordNumber: 2 ♀, 1 AD; recordedBy: JŁ; **Location:** county: NOR-Sogn og Fjordane; locality: In the vicinity of Skjolden; verbatimElevation: 75; decimalLatitude: 61.4992; decimalLongitude: 7.6094; **Event:** samplingProtocol: U; eventDate: 08/09/2007; habitat: Slope near road, very moist grass, moss, soil
- **Type status:** Other material. **Occurrence:** recordNumber: 1 ♀; recordedBy: JŁ; **Location:** county: NOR-Sogn og Fjordane; locality: Skjolden; verbatimElevation: 25; decimalLatitude: 61.4919; decimalLongitude: 7.6058; **Event:** samplingProtocol: U; eventDate: 10/09/2007; habitat: Moderately moist meadow, close to river and forest, grass, moss and soil
- **Type status:** Other material. **Occurrence:** recordNumber: 3 ♀; recordedBy: JŁ; **Location:** county: NOR-Sogn og Fjordane; locality: Skjolden; verbatimElevation: 25; decimalLatitude: 61.4911; decimalLongitude: 7.6056; **Event:** samplingProtocol: U; eventDate: 11/09/2007; habitat: River bank, grass, moss, in humic soil
- **Type status:** Other material. **Occurrence:** recordNumber: 1 ♀, 3 DN; recordedBy: JŁ; **Location:** county: NOR-Sogn og Fjordane; locality: In the vicinity of Skjolden; verbatimElevation: 75; decimalLatitude: 61.4992; decimalLongitude: 7.6094; **Event:** samplingProtocol: U; eventDate: 17/09/2007; habitat: Slope near road, very moist grass, moss, soil
- **Type status:** Other material. **Occurrence:** recordNumber: 1 ♀; recordedBy: MG; **Location:** county: NOR-Vestfold; locality: Larvik; verbatimElevation: 100; decimalLatitude: 59.0703; decimalLongitude: 10.0341; **Event:** samplingProtocol: T; eventDate: 03/10/2002; habitat: Moss, soil

##### Distribution

Norway Thor 1900b, Berlese 1910, Haitlinger 2000, Mąkol and Gulvik 2002), Sweden (Andersén 1863) and Finland (Mąkol 2000a, Mąkol et al. 2006).

##### Notes

*Podothrombium magnum* Berlese, 1910 and *Podothrombium roari* Haitlinger, 2000 are junior synonyms (Mąkol 2005).

#### Podothrombium kordulae

Haitlinger, 1995 [L]

http://www.gbif.org/species/4541002

##### Materials

- **Type status:** Other material. **Occurrence:** recordNumber: 3 L; recordedBy: MG; **Location:** county: NOR-Akershus; locality: Østmarka; verbatimElevation: 250; decimalLatitude: 59.8425; decimalLongitude: 11.0338; **Event:** samplingProtocol: T; eventDate: 07/06/2002; habitat: Decomposed wood from the inside of decaying tree trunk
- **Type status:** Other material. **Occurrence:** recordNumber: 2 L; recordedBy: MG; **Location:** county: NOR-Buskerud; locality: Gulsvik; verbatimElevation: 150; decimalLatitude: 60.3811; decimalLongitude: 9.6133; **Event:** samplingProtocol: T; eventDate: 07/06/2001; habitat: Litter, soil
- **Type status:** Other material. **Occurrence:** recordNumber: 6 L; recordedBy: MG; **Location:** county: NOR-Hordaland; locality: Hola; verbatimElevation: 570; decimalLatitude: 60.9019; decimalLongitude: 6.4836; **Event:** samplingProtocol: T; eventDate: 25/06/2005; habitat: Litter, moss
- **Type status:** Other material. **Occurrence:** recordNumber: 2 L; recordedBy: MG, GM; **Location:** county: NOR-Sogn og Fjordane; locality: In the vicinity of Førde; verbatimElevation: 300; decimalLatitude: 61.4108; decimalLongitude: 5.8178; **Event:** samplingProtocol: T; eventDate: 31/07/2001; habitat: Moss, soil, undergrowth
- **Type status:** Other material. **Occurrence:** recordNumber: 2 L; recordedBy: MG, PS, SS; **Location:** county: NOR-Sogn og Fjordane; locality: Hov, in the vicinity of Vik; verbatimElevation: 400; decimalLatitude: 61.3231; decimalLongitude: 6.2603; **Event:** samplingProtocol: T; eventDate: 04/07/2002; habitat: Litter, moss, humus

##### Distribution

New for Norway.

#### Podothrombium strandi

Berlese, 1910 [PL]

http://www.gbif.org/species/4541011

##### Materials

- **Type status:** Other material. **Occurrence:** recordNumber: 1 AD, 2 DN; recordedBy: MG, GM; **Location:** county: NOR-Oppland; locality: Sognefjellet; verbatimElevation: 1 400; decimalLatitude: 61.5617; decimalLongitude: 7.9836; **Event:** samplingProtocol: T; eventDate: 10/08/2000; habitat: Soil, moss, undergrowth
- **Type status:** Other material. **Occurrence:** recordNumber: 2 AD, 1 DN; recordedBy: MG, GM; **Location:** county: NOR-Oppland; locality: Sognefjellet, in the vicinity of Krossbu; verbatimElevation: 1 450; decimalLatitude: 61.5667; decimalLongitude: 8.0183; **Event:** samplingProtocol: T; eventDate: 10/08/2000; habitat: Soil, moss, undergrowth
- **Type status:** Other material. **Occurrence:** recordNumber: 4 AD, 4 DN; recordedBy: MG, GM; **Location:** county: NOR-Oppland; locality: Sognefjellet; verbatimElevation: 1 430; decimalLatitude: 61.5600; decimalLongitude: 8.0014; **Event:** samplingProtocol: T; eventDate: 02/09/2000; habitat: Soil, moss, undergrowth
- **Type status:** Other material. **Occurrence:** recordNumber: 1 DN; recordedBy: MG, JŁ; **Location:** county: NOR-Oppland; locality: Sognefjellet; verbatimElevation: 1 300; decimalLatitude: 61.5600; decimalLongitude: 7.9681; **Event:** samplingProtocol: T; eventDate: 20/08/2006; habitat: Moss, litter
- **Type status:** Other material. **Occurrence:** recordNumber: 1 ♀; recordedBy: MG; **Location:** county: NOR-Sogn og Fjordane; locality: Hegg; verbatimElevation: 400; decimalLatitude: 61.0808; decimalLongitude: 7.8981; **Event:** samplingProtocol: T; eventDate: 26/08/2000; habitat: Rot from decaying tree trunk, by the stream

##### Distribution

Norway (Berlese 1910) and Finland (Mąkol et al. 2006).

#### Podothrombium svalbardense

Oudemans, 1930 [L]

http://www.gbif.org/species/4541030

##### Materials

- **Type status:** Other material. **Occurrence:** recordNumber: 2 L; recordedBy: MG, GM; **Location:** county: NOR-Oppland; locality: Sognefjellet; verbatimElevation: 1 430; decimalLatitude: 61.5600; decimalLongitude: 8.0014; **Event:** samplingProtocol: T; eventDate: 02/09/2000; habitat: Soil, moss, undergrowth

##### Distribution

Norway (Oudemans 1930 in: Thor 1930).

#### Podothrombium sp.

##### Materials

- **Type status:** Other material. **Occurrence:** recordNumber: 4 L; recordedBy: MG; **Location:** county: NOR-Hordaland; locality: Trondsbu, Hardangervidda; verbatimElevation: 1 200; decimalLatitude: 60.2906; decimalLongitude: 7.5481; **Event:** samplingProtocol: T; eventDate: 22/08/1997; habitat: Moist soil, *Salix lapponum* trees

##### Notes

Potential new species for science from Norway. Morphologically near *Podothrombium piriforme* Robaux and Schiess, 1982.

#### Tanaupodidae

Thor, 1935

#### Rhinothrombium nemoricola

(Berlese, 1886) [PL]

http://www.gbif.org/species/4540909

##### Distribution

Norway (Mąkol and Gulvik 2002).

#### Tanaupodus bifurcatus

Carl, 1966 [PL]

https://www.gbif.org/species/4540907

##### Distribution

Norway (Carl 1966).

##### Notes

Only single record in literature (Carl 1966) and no recent occurrences since then. Identification questionable.

#### Tanaupodus passimpilosus

Berlese, 1910 [PL]

http://www.gbif.org/species/4540906

##### Distribution

Norway (Oudemans 1927).

##### Notes

Only single record in literature (Oudemans 1927) and no recent occurrences since then. Identification questionable.

#### Trombellidae

Thor, 1935

#### Trombellinae

Thor, 1935

#### Nothrotrombidium otiorum

(Berlese, 1902) [PL, L]

http://www.gbif.org/species/4540922

##### Distribution

Norway (Berlese 1912).

##### Notes

Only single record in literature (Berlese 1912) and no recent occurrences since then. Identification questionable.

#### Trombiculidae

Ewing, 1929

#### Trombiculinae

Ewing, 1929

#### Hirsutiella zachvatkini

(Schluger, 1948) [PL, L]

http://www.gbif.org/species/4541170

##### Materials

- **Type status:** Other material. **Occurrence:** recordNumber: 1 PL; recordedBy: PTL; **Location:** county: FIN-Southwest Finland; locality: Rymättylä, Alakylä, Isoluoto; decimalLatitude: 60.30; decimalLongitude: 21.97; **Event:** eventDate: 04/09/1997; habitat: Litter of alder and decaying reed on seashore

##### Distribution

Norway (Mehl 1979), Sweden (Edler 1969, Edler 1972) and new for Finland.

#### Leptotrombidium russicum

(Oudemans, 1902) [PL, L]

https://www.gbif.org/species/4541159

##### Materials

- **Type status:** Other material. **Occurrence:** recordNumber: 9 L; recordedBy: PTL, AKA; **Location:** county: FIN-Southwest Finland; locality: Raisio, Kaanaa; decimalLatitude: 60.46; decimalLongitude: 22.09; **Event:** samplingProtocol: U; eventDate: 28/08/1986; habitat: Ears of *Eptesicus nilssonii*

##### Distribution

New for Finland.

#### Neotrombicula autumnalis

(Shaw, 1790) [PL, L]

http://www.gbif.org/species/4541106

##### Materials

- **Type status:** Other material. **Occurrence:** recordNumber: 1 AD; recordedBy: PTL; **Location:** county: FIN-Southwest Finland; locality: Nauvo, Gullkrona village; decimalLatitude: 60.0; decimalLongitude: 22.0; **Event:** eventDate: 06/12/1982; habitat: N harbour, tussocks of grass with burrows of *Microtus agrestis*

##### Distribution

Norway (Stekolnikov and Daniel 2012), Sweden (Stekolnikov and Daniel 2012) and Finland (Stekolnikov and Daniel 2012).

##### Notes

We could not locate the published records in Fennoscandia, except for reference to the presence of *N. autumnalis* in Norway, Sweden and Finland, provided by Stekolnikov and Daniel (2012). Moreover, *N. autumnalis* has been reported in health care. One record, probably unpublished, from Öland, Sweden, in Lundqvist's collection (Zoological Museum in Lund).

#### Neotrombicula talmiensis

(Schluger, 1955) [PL, L]

http://www.gbif.org/species/4541104

##### Materials

- **Type status:** Other material. **Occurrence:** recordNumber: 1 DN; recordedBy: MG; **Location:** county: NOR-Sogn og Fjordane; locality: Between Skjolden and Luster; verbatimElevation: 25; decimalLatitude: 61.4719; decimalLongitude: 7.5461; **Event:** samplingProtocol: T; eventDate: 05/11/2005; habitat: Forest edge, sandy-clayey soil, moss

##### Distribution

New for Norway.

#### Neotrombicula vulgaris

(Schluger, 1955) [L]

https://www.gbif.org/species/4541093

##### Materials

- **Type status:** Other material. **Occurrence:** recordNumber: 3 L; recordedBy: MG; **Location:** county: NOR-Sogn og Fjordane; locality: Near Kjøsnesfjorden, in the vicinity of Kjøsnes; verbatimElevation: 250; decimalLatitude: 61.5419; decimalLongitude: 6.5378; **Event:** samplingProtocol: T; eventDate: 11/10/2000; habitat: Rot from decaying tree trunk
- **Type status:** Other material. **Occurrence:** recordNumber: 1 L; recordedBy: MG, GM; **Location:** county: NOR-Sogn og Fjordane; locality: In the vicinity of Urnes; verbatimElevation: 50; decimalLatitude: 61.3072; decimalLongitude: 7.3422; **Event:** samplingProtocol: T; eventDate: 19/08/2001; habitat: Soil
- **Type status:** Other material. **Occurrence:** recordNumber: 5 L; recordedBy: MG, GM; **Location:** county: NOR-Sogn og Fjordane; locality: Kroken; verbatimElevation: 50; decimalLatitude: 61.3403; decimalLongitude: 7.3967; **Event:** samplingProtocol: T; eventDate: 19/08/2001; habitat: Soil at the base of elm tree
- **Type status:** Other material. **Occurrence:** recordNumber: 1 L; recordedBy: MG; **Location:** county: NOR-Sogn og Fjordane; locality: Mørkri, in the vicinity of Skjolden; verbatimElevation: 150; decimalLatitude: 61.5567; decimalLongitude: 7.6208; **Event:** samplingProtocol: T; eventDate: 30/08/2005; habitat: Common nettle, cocksfoot, tufted hair grass, litter

##### Distribution

New for Norway.

#### Hannemania

Oudemans, 1911

##### Distribution

Sweden (Sellnick 1949).

##### Notes

Only nymph of *Hannemannia* recorded (Sellnick 1949) and no recent occurrences since then. Identification questionable.

#### Trombidiidae

Leach, 1815

#### Allothrombiinae

Thor, 1935

#### Allothrombium fuliginosum

(Hermann, 1804) [PL, L]

http://www.gbif.org/species/4540875

##### Materials

- **Type status:** Other material. **Occurrence:** recordNumber: 1 DN; recordedBy: MG; **Location:** county: NOR-Sogn og Fjordane; locality: Between Skjolden and Luster; verbatimElevation: 25; decimalLatitude: 61.4719; decimalLongitude: 7.5461; **Event:** samplingProtocol: T; eventDate: 05/11/2005; habitat: Soil
- **Type status:** Other material. **Occurrence:** recordNumber: 1 AD; recordedBy: JŁ; **Location:** county: NOR-Sogn og Fjordane; locality: Skjolden; verbatimElevation: 25; decimalLatitude: 61.4911; decimalLongitude: 7.6008; **Event:** samplingProtocol: U; eventDate: 05/09/2007; habitat: Stone wall covered with moss, very dry
- **Type status:** Other material. **Occurrence:** recordNumber: 10 ♂, 16 AD, 5 DN; recordedBy: JŁ; **Location:** county: NOR-Sogn og Fjordane; locality: Skjolden; verbatimElevation: 25; decimalLatitude: 61.4911; decimalLongitude: 7.6008; **Event:** samplingProtocol: U; eventDate: 10/09/2007; habitat: Stone wall covered with moss, very dry

##### Distribution

Norway (Thor 1900b, Haitlinger 2000), Sweden (Andersén 1863) and Finland (Krogerus 1960, Mąkol et al. 2006).

#### Paratrombiinae

Feider, 1959

#### Paratrombium insulare

(Berlese, 1910) [PL, L]

http://www.gbif.org/species/4540766

##### Distribution

Norway (Mąkol and Gulvik 2002) and Finland (Mąkol 2000b, Mąkol et al. 2006).

#### Trombidiinae

Leach, 1815

#### Trombidium brevimanum

(Berlese, 1910) [PL, L]

http://www.gbif.org/species/4540853

##### Distribution

Norway (Berlese 1912) and Finland (Nordberg 1936, Mąkol et al. 2006).

#### Trombidium geniculatum

(Feider, 1955) [PL, L]

http://www.gbif.org/species/4540844

##### Materials

- **Type status:** Other material. **Occurrence:** recordNumber: 1 ♂; recordedBy: MG; **Location:** county: NOR-Vestfold; locality: Larvik, Bøkeskogen; verbatimElevation: 50; decimalLatitude: 59.0592; decimalLongitude: 10.0200; **Event:** samplingProtocol: T; eventDate: 03/10/2002; habitat: Litter, soil
- **Type status:** Other material. **Occurrence:** recordNumber: 1 L; recordedBy: SMTP; **Location:** county: SWE-Uppsala; locality: Biskops-Arnö (=TrapID 8); verbatimElevation: 10; decimalLatitude: 59.6721; decimalLongitude: 17.5009; **Event:** samplingProtocol: M; eventDate: 20/06/2005 - 18/07/2005 (=Coll.ID 1602); habitat: Northern beach, elm grove

##### Distribution

Norway (Mąkol and Gulvik 2002) and new for Sweden.

#### Trombidium heterotrichum

(Berlese, 1910) [PL]

http://www.gbif.org/species/4540829

##### Distribution

Norway (Berlese 1910) and Finland (Mąkol 2003).

#### Trombidium holosericeum

(Linnaeus, 1758) [PL, L]

http://www.gbif.org/species/4540860

##### Materials

- **Type status:** Other material. **Occurrence:** recordNumber: 3 PL; recordedBy: JS, MF, RH; **Location:** county: SWE-Jönköping; locality: Jönköping, Shore of Lake Vättern; verbatimElevation: 100; decimalLatitude: 57.7933; decimalLongitude: 14.2565; **Event:** samplingProtocol: U; eventDate: 09/06/2013; habitat: Maple and birch copse on shore, sand, leaf, bryophytes
- **Type status:** Other material. **Occurrence:** recordNumber: 1 L; recordedBy: SMTP; **Location:** county: SWE-Uppsala; locality: Biskops-Arnö (=TrapID 8); verbatimElevation: 10; decimalLatitude: 59.6721; decimalLongitude: 17.5009; **Event:** samplingProtocol: M; eventDate: 20/06/2005 - 18/07/2005 (=Coll.ID 1602); habitat: Northern beach, elm grove

##### Distribution

Norway (Thor 1900b, Haitlinger 2000,Mąkol 2002,Mąkol and Gulvik 2002), Sweden (Linnaeus 1758, de Geer 1778, Sellnick 1958) and Finland (Nordberg 1936, Krogerus 1960, Mąkol and Wohltmann 2000, Mąkol et al. 2006).

##### Notes

*Acarus parasiticus* de Geer, 1778 is a junior synonym.

#### Trombidium latum

C.L. Koch, 1837 [PL, L]

http://www.gbif.org/species/4540816

##### Materials

- **Type status:** Other material. **Occurrence:** recordNumber: 1 ♀; recordedBy: MG; **Location:** county: NOR-Buskerud; locality: Ørgenvika; verbatimElevation: 150; decimalLatitude: 60.2858; decimalLongitude: 9.6878; **Event:** samplingProtocol: T; eventDate: 06/06/2001; habitat: Soil, litter
- **Type status:** Other material. **Occurrence:** recordNumber: 1 AD; recordedBy: MG; **Location:** county: NOR-Hordaland; locality: Skreidi, between Vaksdal and Fossmarki; verbatimElevation: 52; decimalLatitude: 60.4939; decimalLongitude: 5.7339; **Event:** samplingProtocol: T; eventDate: 06/07/2001; habitat: Humus
- **Type status:** Other material. **Occurrence:** recordNumber: 1 DN; recordedBy: MG; **Location:** county: NOR-Sogn og Fjordane; locality: Grinde; verbatimElevation: 120; decimalLatitude: 61.1847; decimalLongitude: 6.7406; **Event:** samplingProtocol: T; eventDate: 12/11/2000; habitat: Rot from tree trunk
- **Type status:** Other material. **Occurrence:** recordNumber: 1 ♂, 2 AD, 1 DN,; recordedBy: MG; **Location:** county: NOR-Sogn og Fjordane; locality: In the vicinity of Skjolden; verbatimElevation: 50; decimalLatitude: 61.4903; decimalLongitude: 7.5736; **Event:** samplingProtocol: T; eventDate: 08/05/2002; habitat: Litter, soil, moss
- **Type status:** Other material. **Occurrence:** recordNumber: 2 AD; recordedBy: MG; **Location:** county: NOR-Sogn og Fjordane; locality: Luster; verbatimElevation: 25; decimalLatitude: 61.4458; decimalLongitude: 7.4661; **Event:** samplingProtocol: U; eventDate: 13/05/2002; habitat: Litter
- **Type status:** Other material. **Occurrence:** recordNumber: 1 AD; recordedBy: MG; **Location:** county: NOR-Sogn og Fjordane; locality: In the vicinity of Opptun; verbatimElevation: 600; decimalLatitude: 61.4978; decimalLongitude: 7.7492; **Event:** samplingProtocol: U; eventDate: 26/04/2005; habitat: Litter
- **Type status:** Other material. **Occurrence:** recordNumber: 1 AD; recordedBy: MG; **Location:** county: NOR-Sogn og Fjordane; locality: Mørkri, in the vicinity of Skjolden; verbatimElevation: 150; decimalLatitude: 61.5567; decimalLongitude: 7.6208; **Event:** samplingProtocol: T; eventDate: 30/08/2005; habitat: Meadow with trees, rot from decaying elm tree trunk
- **Type status:** Other material. **Occurrence:** recordNumber: 1 AD, 1 DN; recordedBy: MG; **Location:** county: NOR-Sogn og Fjordane; locality: Between Opptun and Fortun; verbatimElevation: 600; decimalLatitude: 61.4978; decimalLongitude: 7.7492; **Event:** samplingProtocol: T; eventDate: 15/11/2005; habitat: Soil, moss, wet ground
- **Type status:** Other material. **Occurrence:** recordNumber: 1 DN; recordedBy: MG, MW; **Location:** county: NOR-Sogn og Fjordane; locality: In the vicinity of Skjolden; verbatimElevation: 75; decimalLatitude: 61.4994; decimalLongitude: 7.6097; **Event:** samplingProtocol: T; eventDate: 09/12/2006; habitat: House garden, decaying plants
- **Type status:** Other material. **Occurrence:** recordNumber: 1 DN; recordedBy: JŁ; **Location:** county: NOR-Sogn og Fjordane; locality: In the vicinity of Skjolden; verbatimElevation: 75; decimalLatitude: 61.4992; decimalLongitude: 7.6094; **Event:** samplingProtocol: U; eventDate: 08/09/2007; habitat: Slope near road, very moist grass, moss, soil
- **Type status:** Other material. **Occurrence:** recordNumber: 1 AD; recordedBy: JŁ; **Location:** county: NOR-Sogn og Fjordane; locality: Skjolden; verbatimElevation: 25; decimalLatitude: 61.4903; decimalLongitude: 7.6014; **Event:** samplingProtocol: U; eventDate: 11/09/2007; habitat: Alder trees by the river, grass, moss, damp soil
- **Type status:** Other material. **Occurrence:** recordNumber: 1 DN; recordedBy: JŁ; **Location:** county: NOR-Sogn og Fjordane; locality: Skjolden; verbatimElevation: 25; decimalLatitude: 61.4933; decimalLongitude: 7.6081; **Event:** samplingProtocol: U; eventDate: 17/09/2007; habitat: Birch and pine trees surrounded by buildings, soil
- **Type status:** Other material. **Occurrence:** recordNumber: 1 DN; recordedBy: MG; **Location:** county: NOR-Vestfold; locality: In the vicinity of Nalum; verbatimElevation: 50; decimalLatitude: 58.9894; decimalLongitude: 9.9853; **Event:** samplingProtocol: T; eventDate: 03/10/2002; habitat: Soil, litter
- **Type status:** Other material. **Occurrence:** recordNumber: 1 L; recordedBy: SMTP; **Location:** county: SWE-Södermanland; locality: Tullgarns näs, Rävsalaviken (=TrapID 30); verbatimElevation: 15; decimalLatitude: 58.9552; decimalLongitude: 17.6075; **Event:** samplingProtocol: M; eventDate: 03/07/2004 - 19/08/2004 (=Coll.ID 1055); habitat: Mixed forest next to pasture

##### Distribution

Norway (Mąkol 2002, Mąkol and Gulvik 2002), Sweden (Andersén 1863) and Finland (Mąkol et al. 2006).

#### Trombidium mediterraneum

(Berlese, 1910) [PL, L]

http://www.gbif.org/species/4540833

##### Materials

- **Type status:** Other material. **Occurrence:** recordNumber: 1 AD; recordedBy: MG; **Location:** county: NOR-Buskerud; locality: Morud; verbatimElevation: 200; decimalLatitude: 60.0603; decimalLongitude: 9.8594; **Event:** samplingProtocol: T; eventDate: 06/06/2001; habitat: Soil, undergrowth
- **Type status:** Other material. **Occurrence:** recordNumber: 10 L; recordedBy: MG; **Location:** county: NOR-Buskerud; locality: Morud; verbatimElevation: 200; decimalLatitude: 60.0603; decimalLongitude: 9.8594; **Event:** samplingProtocol: T; eventDate: 06/06/2001; habitat: Soil, litter, undergrowth
- **Type status:** Other material. **Occurrence:** recordNumber: 1 AD, 1 DN; recordedBy: MG; **Location:** county: NOR-Buskerud; locality: Ørgenvika; verbatimElevation: 150; decimalLatitude: 60.2858; decimalLongitude: 9.6878; **Event:** samplingProtocol: T; eventDate: 06/06/2001; habitat: Litter, soil
- **Type status:** Other material. **Occurrence:** recordNumber: 1 DN; recordedBy: MG; **Location:** county: NOR-Hordaland; locality: Skreidi, between Vaksdal and Fossmarki; verbatimElevation: 50; decimalLatitude: 60.4939; decimalLongitude: 5.7339; **Event:** samplingProtocol: T; eventDate: 06/07/2001; habitat: Moss, soil, humus

##### Distribution

Norway (Judson and Mąkol 2011).

#### Trombidium rimosum

C. L. Koch, 1837 [PL]

http://www.gbif.org/species/4540852

##### Distribution

Sweden (Sellnick 1949) and Finland (Mąkol et al. 2006).

##### Notes

*Sericothrombium meyeri* Krausse, 1916 is a junior synonym (Mąkol 2005).

## Discussion

Our study is part of the Swedish Taxonomy Initiative (STI) - one of the most ambitious All Taxa Biodiversity Inventories (ATBI) in the world (Ronquist and Gärdenfors 2003). One of the pillars in STI is to support taxonomic research on the most neglected taxonomic groups with the aim of raising the basic level of knowledge. For poorly known groups, the first step is an inventory resulting in a checklist of species. While doing so, it is also beneficial to make available and publish the primary data. There are many reasons for this: facilitate the re-use of biological data, facilitate integration with other datasets and increase the potential for interdisciplinary research, transparency and increased quality of science, citeability, credit and monitoring use of collection data (Chavan and Penev 2011, Penev et al. 2011). This is becoming increasingly recognised by funding agencies and national governmental organisations in many countries.

The higher diversity, 80 species, for Norway is likely a result of the comparatively larger effort (71% of all visited localities were in Norway) and more recent studies (Mąkol and Gulvik 2002). The Norwegian non-marine arthropod fauna is commonly a smaller subset of the Swedish fauna, lacking more southern species occurring in Sweden. For some groups, however, Norway may instead house more oceanic or arctic species. Apart from collecting effort, the different number of species-level taxa, as well as species composition observed in Sweden, Finland and Norway, may also be due to biogeographical factors, landscape architecture and actual variety of biotopes. Solhøy et al. (1975) pointed to the decreasing tendency observed in the number of species along the gradient between the eastern and north-eastern territories of Finland, covered with continental tundra and towards alpine tundra influenced by Atlantic climate in Norway. Finland often also lacks some southern species occurring in Sweden, but instead may have eastern fauna elements not occurring in either Sweden or Norway. Even in Norway, the geographical area visited was quite limited, with most effort being spent in the county Sogn og Fjordane. Due to the intense collecting effort in different microhabitats, many species were recorded. Although the fauna is expected to be still more diverse due to the unsampled counties, the majority of species are only known from either larvae or active postlarval forms, which can contribute to some overestimations. Nevertheless, with the current classification, we predict that, if the same effort as in Norway was made in Sweden, over 80 species could be recorded in Sweden alone. The most frequent species was *Calyptostoma velutinum* (Müller, 1776), which was collected 70 times. The high number of observations occurring throughout the season may be due to the specific biology and phenology of *Calyptostoma* sp. reflected in synchronous occurrence of active life instars or it may reflect the occurrence of a species complex as suggested by Wohltmann et al. (1999). A DNA Barcoding effort of terrestrial Parasitengona mites could significantly help charting the diversity in Fennoscandia, reveal overlooked species or identify species complexes.

## References

[B5204564] Andersén C. H. (1863). Bidrag till kännedomen om Nordiska Acarider. Öfversigt af Kongl. Vetenskaps-akademiens forhandlingar.

[B3516833] Bagge A. M., Bagge P. (2009). Finnish water mites (Acari: Hydrachnidia, Halacaroidea). Memoranda Societatis pro Fauna et Flora Fennica.

[B3219382] Berlese A. (1910). Brevi diagnosi di generi e specie nuovi di acari.. Redia.

[B3195193] Berlese A., van der Hammen L. (1912). Trombidiidae. Prospetto dei generi e delle specie finora noti. Redia, 8: 1-291.. Complete Acarological Works, Collected acarological papers previously published in Redia, 1903-1923.

[B3195058] Beron P. (2008). Acarorum Catalogus I. Acariformes: Calyptostomatoidea (Calyptostomatidae), Erythraeoidea (Smarididae, Erythraeidae)..

[B5206837] Buitendijk A. M. (1945). Voorloopig catalogus van de Acari in de collectie Oudemans. Zoologische Mededelingen.

[B3240488] Carl K. P. (1966). Eine neue europaische Trombidiide, *Tanaupodus
bifurcatus* n. sp. (Acari: Trombidiformes).. Revue d'Ecologie et Biologie du Sol.

[B3516843] Chavan Vishwas, Penev Lyubomir (2011). The data paper: a mechanism to incentivize data publishing in biodiversity science. BMC Bioinformatics.

[B3516687] Dabert Miroslawa, Proctor Heather, Dabert Jacek (2016). Higher-level molecular phylogeny of the water mites (Acariformes: Prostigmata: Parasitengonina: Hydrachnidiae). Molecular Phylogenetics and Evolution.

[B5201227] de Geer Charles (1778). Mémoires pour l’Histoire des Insectes. Des Mittes et des Faucheurs. Second Memoire.. Mémoires pour Servir a l’Histoire des Insectes.

[B5204574] Edler A. (1969). Ectoparasitic mites (Acarina) from small mammals in Central Sweden. Entomologisk Tidskrift.

[B5227671] Edler A. (1972). Ectoparasitic mites (Acarina) from small mammals in southern Sweden. Entomologisk Tidskrift.

[B5205439] Gabryś G. (1999). The World genera of Microtrombidiidae (Acari, Actinedida, Trombidioidea). Monographs of the Upper Silesian Museum.

[B5206739] Gabryś G., Wohltmann A., Mąkol J. (2005). A redescription of *Platytrombidium
fasciatum* (C. L. Koch, 1836) and *Atractothromiıum sylvaticum* (C. L. Koch, 1835) (Acari: Parasitengona: Microtrombidiidae) with notes on synonymy, biology and life cycle. Annales Zoologici.

[B3195169] Gabryś G., Roland E., Mąkol J., Lehtinen P. T. (2009). Erythraeoidea (Acari: Prostigmata: Parasitengona) of Finland – state of knowledge and new data.. Zeszyty Naukowe Uniwersytetu Przyrodniczego we Wrocławiu: Biologia i Hodowla Zwierząt.

[B5201396] Gabryś G. (2016). Commentaries on synonyms within Palaearctic Erythraeidae (Acari: Actinotrichida: Parasitengona). Rocznik Muzeum Górnośląskiego (Bytom), Przyroda.

[B3527083] Gärdenfors U., Hall R., Hallingbäck T., Hansson H. G., Hedström L. (2003). Djur, svampar och växter i Sverige 2003: förteckning över antal arter per familj.

[B5205148] Haitlinger R. (2000). Mites (Acari: Prostigmata: Erythraeidae, Trombidiidae) new to the fauna of Norway, Finland, Russia, Latvia and Lithuania, with a description of *Podothrombium
roari* n. sp. Entomologica Fennica, 11: 187–193.. Entomologica Fennica.

[B3195111] Haitlinger R. (2008). New records of mites (Acari: Prostigmata: Erythraeidae, Johnstonianidae, Trombidiidae) from West and North Europe, with the description of *Abrolphus
nymindegabicus* sp. n.. Zeszyty Naukowe Uniwersytetu Przyrodniczego we Wrocławiu, Biologia i Hodowla Zwierząt.

[B5205299] Hull J. E. (1918). Terrestrial Acari of the Tyne Province. Transactions of the Natural History Society of Northumberland, Durham and Newcastle-upon-Tyne, New Series.

[B5207156] Judson Mark L. I., Mąkol Joanna (2011). Pseudoscorpions (Chelonethi: Neobisiidae) parasitized by mites (Acari: Trombidiidae, Erythraeidae). Journal of Arachnology.

[B5210665] Karppinen E. (1958). Beobachtungen über das Vorkommen von Arten der Familie Erythraeidae (Acar.) in Finnland sowie Veränderungen in deren Nomenklatur. Annales Entomologici Fennici.

[B5210695] Krogerus R. (1960). Ökologische Studien über nordische Moorarthropoden. Societas Scientiarum Fennica, Commentationes Biologicae.

[B5201608] Łaydanowicz Joanna, Mąkol Joanna (2008). Species diversity of Parasitengona Terrestria (Acari: Actinotrichida: Prostigmata) in a Habitat Influenced by Anthropopressure. Annales Zoologici.

[B5201537] Linnaeus C. (1758). Systema naturae per regna tria naturae, secundum classes, ordines, genera, species, cum caracteribus, differentis, synonymis, locis. 10 th Edition. Impensis Direct.

[B3516748] Lundblad O. (1927). Die Hydracarinen Schwedens I. Beitrag zur Systematik, Embryologie, Ökologie und Verbreitungsgeschichte der schwedischen Arten.

[B3516757] Lundblad O. (1962). Die Hydracarinen Schwedens II. Arkiv för Zoologi, band 14.

[B3516766] Lundblad O. (1968). Die Hydracarinen Schwedens III. Arkiv för Zoologi, band 21.

[B5210685] Mąkol J. (2000). Description of larva of *Podothrombium
filipes* (C.L. Koch 1837) (Acari: Actinotrichida, Trombidiidae) with notes on variability, anomaly and their implications for classification of *Podothrombium* larvae. Annales Zoologici.

[B5210705] Mąkol J. (2000). Description of larva and deutonymph of *Paratrombium
insulare* (Berlese, 1910) (Acari: Actinotrichida: Trombidioidea) with characteristics of adult instar and remarks on other members of the genus. Annales Zoologici.

[B5210735] Mąkol J., Wohltmann A. (2000). A redescription of *Trombidium
holosericeum* (Linnaeus, 1758) (Acari: Actinotrichida: Trombidioidea) with characteristics of all active instars and notes on taxonomy and biology. Annales Zoologici.

[B5205158] Mąkol J. (2002). A redescription of *Trombidium
latum* (C.L. Koch, 1837) (Acari: Actinotrichida,Trombidioidea) with characteristics of all active instars. Annales Zoologici.

[B5201372] Mąkol J., Gabryś G. (2002). A redescription of *Sucidothrombium
sucidum* (L. Koch 1879) (Acari: Actinotrichida, Microtrombidiidae) with characteristics of all active instars. Annales Zoologici.

[B3240510] Mąkol J., Gulvik M. E., Ignatowicz S. (2002). Parasitengona terrestria (Acari: Actinotrichida) of Sogn og Fjordane (Norway).. Postępy polskiej akarologii. Wydawnictwo SGGW..

[B5210715] Mąkol J. (2003). A redescription of *Trombidium
heterotrichum* (Berlese, 1910) (Acari: Actinotrichida, Trombidioidea) from Berlese Acaroteca. Redia.

[B5205290] Mąkol J. (2005). Trombidiidae (Acari: Actinotrichida: Trombidioidea) of Poland.

[B3531545] Mąkol J, Lehtinen PT, Rinne V, Gabryś G, Ignatowicz S (2006). A contribution to the knowledge of Trombidiidae (Acari, Actinotrichida, Trombidioidea) of Finland. Advances in Polish Acarology.

[B5207146] Mąkol J., Łaydanowicz J. (2010). A new species of *Valgothrombium* Willmann, 1940, with additional taxonomic data for Valgothrombiinae genera known as larvae (Acari: Prostigmata: Microtrombidiidae). Zootaxa.

[B5206847] Mąkol J., Gabryś G., Łaydanowicz J. (2011). *Leptus
phalangii* (De Geer, 1778) (Acari: Actinotrichida: Prostigmata) – redescription, ecology and taxonomic notes on its relatives. Annales Zoologici.

[B3195018] Mąkol Joanna, Wohltmann Andreas (2012). An annotated checklist of terrestrial Parasitengona (Actinotrichida: Prostigmata) of the World, Excluding Trombiculidae and Walchiidae. Annales Zoologici.

[B3195028] Mąkol Joanna, Wohltmann Andreas (2013). Corrections and additions to the checklist of terrestrial Parasitengona (Actinotrichida: Prostigmata) of the World, Excluding Trombiculidae and Walchiidae. Annales Zoologici.

[B5168772] Mąkol J., Korniluk M. (2017). *Blankaartia
acuscutellaris* (Walch, 1922) (Actinotrichida: Trombiculidae) collected from the great snipe *Gallinago
media* (Latham, 1787) (Charadriformes: Scolopacidae) in Poland – new host and country record for chigger mite genus and species.. Acarologia.

[B3240625] Mehl R. (1979). Checklist of Norwegian ticks and mites (Acari).. Fauna Norvegica Ser. B.

[B5206729] Moniuszko H., Mąkol J. (2014). Chigger mites (Actinotrichida: Parasitengona, Trombiculidae) of Poland. An updated distribution and hosts. Annals of Parasitology.

[B5210675] Nordberg S. (1936). Biologisch-ökologische Untersuchungen über die Vogelnidicolen. Acta Zoologica Fennica.

[B5207406] Oudemans A. C. (1903). Acarologische Aanteekeningen VII. Entomologische Berichten (Amst.).

[B5204359] Oudemans A. C. (1912). Die bis jetzt bekannten Larven von Thrombidiidae und Erythraeidae mit besonderer Beruecksichtigung der feuer den Menschen schadlichen Arten. Zoologische Jahrbücher.

[B3219335] Oudemans A. C. (1927). Acarologische Aanteekeningen. LXXXVIII.. Entomologische Berrichten Amsterdam.

[B5205375] Oudemans A. C. (1929). Kritisch historisch overzicht der Acarologie. Tweede gedeelte, 1759 – 1804. Tijdschrift voor Entomologie, Suppl..

[B5218086] Oudemans A. C. (1937). Kritisch Historisch Overzicht der Acarologie.

[B3532122] Penev Lyubomir, Mietchen Daniel, Chavan Vishwas, Hagedorn Gregor, Remsen David, Smith Vincent, Shotton David (2011). Pensoft data publishing policies and guidelines for biodiversity data. Pensoft Publishers.

[B3531535] Roland E, Gabryś G (2013). Description of deutonymphs of *Kamertonia
polonica* Gabryś ,2000 (Acari: Actinotrichida: Erythraeidae). Biological Letters.

[B3516677] Ronquist Fredrik, Gärdenfors Ulf (2003). Taxonomy and biodiversity inventories: time to deliver. Trends in Ecology & Evolution.

[B3195067] Sellnick M. (1949). Milben von der Küste Schwedens.. Entomologisk Tidskrift.

[B3195077] Sellnick M. (1958). Milben aus landwirtschaftlichen Betrieben Nordschwedens.. Meddelanden Statens Växtskyddsanstalt.

[B5207175] Solhøy T., Østbye E., Kauri H., Hagen A., Lien L., Skar H. J., Wielgolaski F. E. (1975). Faunal structure of Hardangervidda, Norway. Fennoscandian tundra ecosystems.

[B5201487] Sömermaa K. (1973). Versuche mit chemischer Bekampfung von *Javesella
pellucida* (F.) (Hem., Delphacidae) und Beobachtungen uber Raubmilben (Acar., Prostigmata und Gamasina). Statens Vaxtskyddsanstalt Meddelanden.

[B5201183] Southcott RV (1991). A further revision of *Charletonia* (Acarina: Erythraeidae) based on larvae, protonymphs and deutonymphs.. Invertebrate Taxonomy.

[B3219345] Southcott R. V. (1992). Revision of the larvae of *Leptus* Latreille (Acarina, Erythraeidae) of Europe and North America, with descriptions of post-larval instars.. Zoological Journal of the Linnean Society.

[B5201362] Southcott R. V. (1994). Revision of the larvae of the Microtrombidiinae (Acarina: Microtrombidiidae), with notes on life histories. Zoologica.

[B5227653] Stålstedt Jeanette, Bergsten Johannes, Ronquist Fredrik (2013). "Forms" of water mites (Acari: Hydrachnidia): intraspecific variation or valid species?. Ecology and evolution.

[B5166861] Stålstedt Jeanette, Wohltmann Andreas, Bergsten Johannes, Mąkol Joanna (2016). Towards resolving the double classification in *Erythraeus* (Actinotrichida: Erythraeidae): matching larvae with adults using 28S sequence data and experimental rearing. Organisms Diversity & Evolution.

[B5166871] Stålstedt Jeanette (2017). Phylogeny, taxonomy and species delimitation of water mites and velvet mites..

[B5214668] Stekolnikov A., Daniel M. (2012). Chigger mites (Acari: Trombiculidae) of Turkey. Zootaxa.

[B3516803] Thor S. (1897). Bidrag til kundskaben om Norges Hydrachnider. Archiv for Mathematik og Naturvidenskab.

[B3516793] Thor S. (1897). Andet bidrag til kundskaben om Norges Hydrachnider. Archiv for Mathematik og Naturvidenskab.

[B3516813] Thor S. (1899). Tredie bidrag til kundskaben om Norges hydrachnider. Archiv for Mathematik og Naturvidenskab.

[B3219325] Thor S. (1900). Første undersøgelse af Norges Rhyncholophidae. Christiania.

[B5205138] Thor S. (1900). Første undersøgelse af Norges Trombidiidae. Christiania.

[B3516823] Thor S. (1901). Fjerde bidrag til kundskaben om Norges hydrachnider. Archiv for Mathematik og Naturvidenskab.

[B5206776] Thor S. (1930). Beiträge zur Kenntnis der Invertebraten Fauna von Svalbard. Scrifter om Svalbard og Ishavet.

[B5204534] Trägårdh I. (1902). Beiträge zur kenntnis der Schwdischen Acaridefauna I. Lappländische Trombiiden und Oribatiden. Bihang till Kongliga Svenska Vetenskaps-Akademiens Handlingar.

[B5201270] Trägårdh I. (1904). Monographie der arktischen Acariden. Fauna Arctica.

[B5198190] Trägårdh Ivar (1910). Acariden aus dem Sarekgebirge.. Naturwissenschaftlichen Untersuchungen des Sarekgebirges in Swedisch – Lappland geleitet von Dr. Axel Hamberg.

[B3516706] Welbourn W. C., Dusbábek F., Bukva V. (1991). Phylogenetic studies of the terrestrial Parasitengona. Modern Acarology.

[B5198180] Willmann C. (1943). Terrestrische Milben aus Schwedisch-Lappland. Archiv für Hydrobiologie.

[B3516734] Witte H., Dusbábek F., Bukva V. (1991). The phylogenetic relationships within the Parasitengonae. Modern Acarology.

[B5207131] Wohltmann A., Wendt F. E., Witte H., Eggers A., Needham G. R., Mitchell R., Horn D. J., Welbourn W. C. (1999). The evolutionary change in the life history patterns in hygrobiontic Parasitengonae (Acari: Prostigmata). Acarology IX.

[B3527063] Wohltmann A., Mąkol J., Gabryś G. (2004). A revision of European Johnstonianinae Thor, 1935 (Acari: Prostigmata: Parasitengona: Trombidioidea). Annales Zoologici, Museum and Institute of Zoology, Polish Academy of Sciences.

